# Novel Synthesis of Substituted 2-Trifluoromethyl and 2-Perfluoroalkyl *N*-Arylpyridinium Compounds—Mechanistic Insights

**DOI:** 10.3390/molecules24122328

**Published:** 2019-06-25

**Authors:** Salem El Kharrat, Philippe Laurent, Laurent Boiteau, Hubert Blancou

**Affiliations:** 1 Laboratoire de Recherche en Chimie Organique et Pharmaceutique, Faculté de Pharmacie et Faculté de Médecine, Université Saint-Joseph (USJ), Rue de Damas, Beirut 11-5076, Lebanon; 2 Institut des Biomolécules Max Mousseron, IBMM, UMR 5247, CNRS, Université de Montpellier, ENSCM, c.c.1706, Place E. Bataillon, CEDEX 5, 34095 Montpellier, France; philippe.laurent@umontpellier.fr (P.L.); laurent.boiteau@umontpellier.fr (L.B.); hubert.blancou@univ-montp2.fr (H.B.)

**Keywords:** *N*-arylpyridinium, Parkinsonism, IEDDA, aza-Robinson, *N*-aryl-tetrahydro-quinoliniums, *N*-aryl-tetrahydro-5*H*-cyclohepta[*b*]pyridiniums, *bis*-anilino-tetrahydropyridine, multicomponent reaction

## Abstract

We report a new one-pot synthesis of 2-trifluoromethylated/2-perfluoroalkylated *N*-aryl-substituted pyridiniums, 5,6,7,8-tetrahydroquinoliniums and 6,7,8,9-tetrahydro-5*H*-cyclohepta[*b*]-pyridinium compounds starting from an activated β-dicarbonyl analogue (here a perfluoro-alkylated gem-iodoacetoxy derivative), an aromatic amine and a (cyclic or acyclic) ketone. The key step of this multicomponent reaction, involves the formation of a 3-perfluoroalkyl-*N*,*N’*-diaryl-1,5-diazapentadiene intermediate, various examples of which were isolated and characterized for the first time, together with investigation of their reactivity. We propose a mechanism involving a concurrent inverse electron demand Diels-Alder or Aza-Robinson cascade cyclisation, followed by a bis-de-anilino-elimination. Noteworthy, a *meta*-methoxy substituent on the aniline directs the reaction towards a 2-perfluoroalkyl-7-methoxyquinoline, resulting from the direct cyclization of the diazapentadiene intermediate, instead of pyridinium formation. This is the first evidence of synthesis of pyridinium derivatives from activated β-dicarbonyls, ketones, and an aromatic amine, the structures of which (both reactants and products) being analogous to species involved in biological systems, especially upon neurodegenerative diseases such as Parkinson’s. Beyond suggesting chemical/biochemical analogies, we thus hope to outline new research directions for understanding the mechanism of in vivo formation of pyridiniums, hence possible pharmaceutical strategies to better monitor, control or prevent it.

## 1. Introduction

Compounds containing a pyridinium moiety are important in natural product chemistry [1,2,3,4] and in organic synthesis [5,6,7]. The presence of various substituents, either on the pyridine ring or at the nitrogen atom of the ring, make these important scaffolds versatile compounds used in various areas, ranging from pharmaceutical to industrial chemical applications. 

Pyridinium compounds are generally employed as acylating agents [8], phase transfer catalysts [9], dyes [1] and cationic surfactants [9]. 1-Alkylpyridinium derivatives which are liquid at rt., so-called ionic liquids, are potential new solvents for synthesis and catalysis [5]. Pyridinium compounds have been also used to achieve asymmetric and regioselective synthesis by additions of Grignard reagent [10]. Moreover, pyridinium compounds are widely applied as synthetic building blocks to obtain substituted pyridines, dihydropyridines or piperidines [11]. Other *N*-methyl-pyridinium derivatives have been investigated as new materials, for example, to allow ionic bonding necessary for molecular packing in self-organized solids [12]. 

On the other hand, quaternary pyridinium derivatives are unsaturated heterocyclic surfactants, generally known for their germicidal properties with a wide range of antimicrobial activity [13,14,15], as well as against some pathogenic species of fungi and protozoa [16]. 

Also, it was reported that quaternary ammonium compounds react with cell walls and have a direct or indirect lethal effect on the cell [17]. Related quaternary pyridinium derivatives have been tested for anticancer [18], and anti-malarial activity [19,20], and as cholinesterase inhibitors for the treatment of Alzheimer’s disease [21,22,23]. 

Moreover, pyridinium ions have been incorporated in drug candidates to improve their water solubility [24,25,26]. Otherwise, the use of pyridinium ions bearing lipophilic groups has been shown to improve their accumulation in mitochondria to scavenge or detect radicals generated there [27,28].

Recently, pyridinium amphiphiles were shown to generate promising transfection systems for gene therapy [29,30,31,32]. Several supramolecular pyridinium containing complexes proved to be extremely efficient nucleic acid delivery systems, displaying excellent serum stability and tissue penetration [30,32]. 

**Scheme 1:**
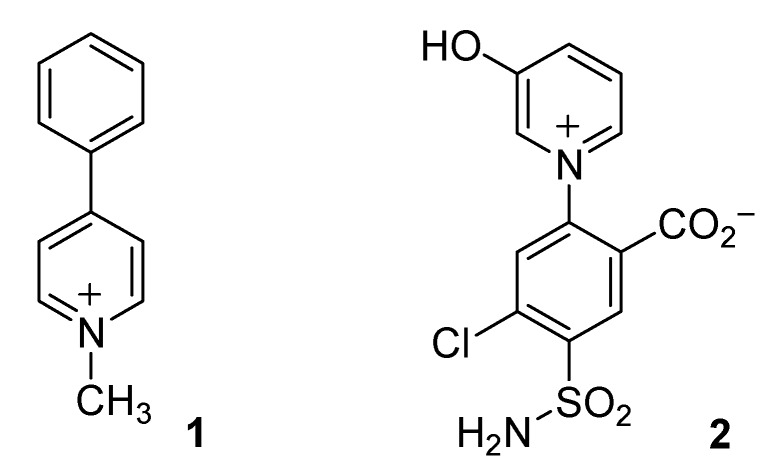
Structure of 1-methyl-4-phenylpyridinium (MPP+) **1** and pyridinium furosemide **2** correlated to neurodegeneration.

The association of pyridinium derivatives with the appearance of Parkinson’s disease, led to a search for pyridinium analogs as possible endogenous or exogenous neurotoxins critical to this neurodegeneration [33]. For example, 1-methyl-4-phenylpyridinium (MPP+) **1** is the most popular molecule used to induce Parkinsonism in vivo (Scheme 1) [34,35,36]. More recently, pyridinium furosemide (PF) **2**, has been also used as a model in helping to identify specific events of Parkinson’s disease [37]. 

Besides, pyridinium containing compounds are used as herbicides [38,39], drugs [13,14,15,16,17,18,19,20,21,22,23,24,25,26,27,28,29,30,31,32], and as intermediates in the synthesis of many heterocyclic compounds [1,2,3,4,5,6,7,11] and are considered as possible exogenous neurotoxins [40,41,42]. However, several lines of evidence suggest that pyridinium derivatives may be formed under endogenous conditions [43], for example during the Maillard reaction between proteins and carbonyl compounds [43,44,45]. 

### Synthesis of Pyridinium Compounds—A Short Literature Survey

**Scheme 2:**
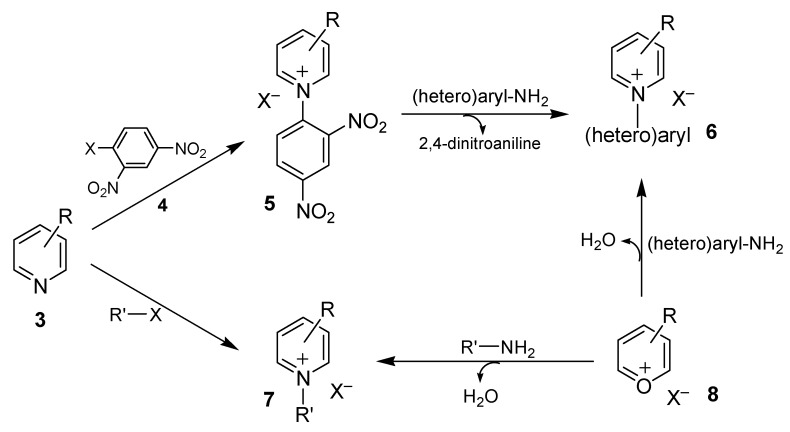
Synthetic routes to *N*-(hetero)arylpyridinium salts **6** and *N*-alkylpyridinium salts **7** starting from pyridines **3** or pyryliums **8** [46,47,48,49,50,51,52,53,54]. R’: alkyl, arylakyl; X: halides, sulfonates; R: electron-donor substituents, X: halides.

The best-known method for the synthesis of *N*-(hetero)arylpyridinium salts is the Zincke amine exchange reaction [46,47,48,49,50], which requires a two-step procedure (Scheme 2): First a pyridine **3** is reacted with 1-chloro-2,4-dinitrobenzene (**4**, X = Cl) to give an *N*-(2,4-dinitrophenyl)pyridinium chloride **5**. This highly electrophilic compound **5** is then reacted with an (hetero)arylamine to give a new *N*-(hetero)arylpyridinium salt **6** with elimination of 2,4-dinitroaniline [46,47,48,49,50]. This reaction will work best if R is an electron-donor substituent. 

Besides, several synthetic routes to *N*-alkylpyridinium compounds **7** are known, but the most commonly used method is the Menschutkin reaction, an SN_2_ reaction of a pyridine derivative **3** with an alkyl halide or sulfonate (R’-X) (Scheme 2) [51,52]. This reaction is favored by electron-donor substituents R on the pyridine ring. Moreover, pyridinium salts **6** and **7** are also synthesized from the ring transformation reaction of pyrylium derivatives **8** with primary alkyl or (hetero)arylamines (Scheme 2) [53,54].

**Scheme 3:**
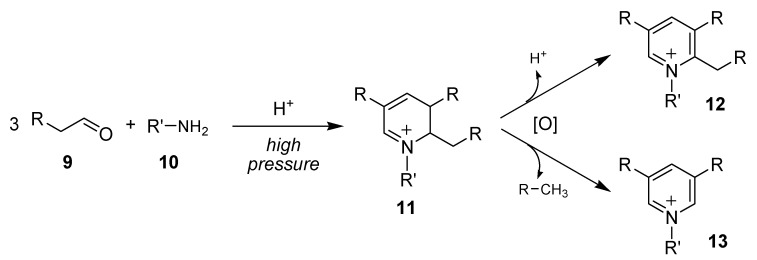
Pyridinium synthetic routes through the Chichibabine reaction [55,56,57,58].

Pyridinium compounds may also be synthesized by the Chichibabine reaction [55,56,57,58] based on the [2+2+1+1] approach, involving an acid-mediated reaction between three equivalents of an enolisable aldehyde **9** and one equivalent of amine **10**. Typically, three products can be isolated from this reaction (Scheme 3); a major product, 1,2,3,5-tetrasubstituted pyridinium **12**, which is formed via the auto-oxidation of the product 1,2,3,5-dihydropyridinium **11**, and a minor product 1,3,5-trisubstituted pyridinium derivative **13**. However, the Chichibabine reaction requires harsh conditions, give low yields with numerous difficult to separate side products [57,58]. 

In previous articles [59,60,61,62,63,64,65] we have reported the synthesis of various perfluoroalkylated quinoline derivatives, by reacting perfluoroalkylated *gem*-iodoacyloxy derivatives **14** with substituted anilines **15**. Later we observed that the presence of a ketone in the medium leads to the formation of arylpyridiniums instead of quinolines; what suggested an interesting alternative to published synthetic methods, and led us to design a new and efficient synthesis of substituted 2-trifluoromethyl and 2-perfluoroalkyl-*N*-arylpyridinium derivatives **17**–**19** under mild reaction conditions and with very good yields. Mechanistic studies addressing possible intermolecular cycloaddition reactions are detailed, underlying possible chemical/biochemical mechanistic analogies in relation to the formation of pyridinium derivatives under biological conditions.

## 2. Results and Discussion

### 2.1. Synthesis

**Scheme 4:**
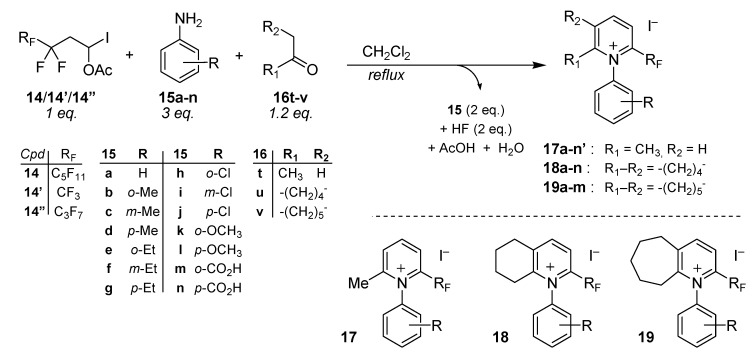
Synthesis (optimized stoichiometries) of trifluoromethylated and perfluoroalkylated 6-methyl-*N*-arylpyridiniums **17a**–**n’**, *N*-aryl-5,6,7,8-tetrahydroquinoliniums **18a–n** and *N*-aryl-6,7,8,9-tetrahydro-5*H*-cyclohepta[*b*]pyridiniums **19a**–**m**, examples in Table 1, Table 2 and Table 3 respectively (bottom right: structures of compound series **17**–**19**).

We report the reaction of 1-acetoxy-2-(perfluoroalkyl)-1-iodo-ethane **14**–**14”** and various anilines 15a-n in the presence of acetone 16t (R_1_ = CH_3_, R_2_ = H), leading to the formation of 2-trifluoromethyl and 2-perfluoroalkyl-6-methyl-*N*-arylpyridinium derivatives **17a**–**n’** in fair to excellent yields (50–90%) (Scheme 4, Table 1). This reaction was then exemplified using two cyclic ketones, namely cyclohexanone **16u** and cycloheptanone **16v**, both of which reacted smoothly to give the corresponding 2-trifluoromethyl-/2-perfluoroalkyl-*N*-(R-phenyl)-5,6,7,8-tetrahydroquinoliniums **18a**–**n** (R_1_-R_2_ = -(CH_2_)_4_-; Table 2) in good to excellent yields (75–90%) and 2-trifluoromethyl-/2-perfluoroalkyl-*N*-(R-phenyl)-6,7,8,9-tetrahydro-5*H*-cyclohepta[*b*]pyridiniums **19a**–**m** (R_1_–R_2_ = -(CH_2_)_5_-; Table 3), respectively. All reactions proceeded with good to very good yields (70–90%).

The numbering scheme for the investigated examples in compound series **17**–**19**, is the following: (i) the numbers **17**–**19** denote the compound series depending on the reacting ketone **16t**–**v**; (ii) the letter **a**–**n** denotes the aryl substituent inherited from the aniline substrate **15**; (iii) the appended **’** or **”** denotes the perfluoroalkyl chain R_F_ from perfluoroalkyl substrate **14**: R_F_ = C_5_F_11_ (**14**), CF_3_ (**14’**) or C_3_F_7_ (**14”**) respectively. For instance, **17a** is obtained from **15a** and **14**, or **17n’** is obtained from **15n** and **14’**.

Thorough screening of reactants stoichiometries and reaction conditions (monitoring by TLC and ^19^F-NMR spectroscopy), allowed us to identify optimized conditions giving the best overall yields: namely one equivalent of *gem*-iodoacetate **14**–**14”**, three equivalents of arylamine **15a–o** and 1.2 equivalents of ketone **16t**–**v** in refluxing anhydrous dichloromethane. Reactions were typically completed within 4–12 h. A straightforward work-up allowed the isolation of pyridinium products **17**–**19**, through: (i) filtering off the excess anilinium salts that precipitated at the end of the reaction, then (ii) precipitating the product from the filtrate by addition of an appropriate solvent. Further purification of **17**–**19** was carried out by final precipitation from dichloromethane-ether mixture, pure 2-perfluoroalkyl-*N*-arylpyridinium iodides **17**–**19** being isolated as amorphous solids. 

### 2.2. Structure Determination of N-Arylpyridiniums ***17***–***19***

**Figure 1:**
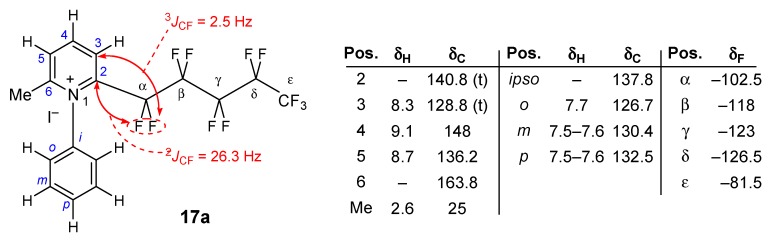
Structure of 6-methyl-2-perfluoropentyl-1-phenylpyridinium **17a** and NMR resonance assignments.

Structural assignments were accomplished without ambiguity by ^1^H-, ^13^C- and ^19^F-NMR as well as 2D ^1^H-^1^H (COSY) and ^1^H-^13^C (HETCOR) NMR correlations. As an example, the NMR spectra of 6-methyl-2-perfluoropentyl-1-phenylpyridinium **17a** (R = R_2_ = H, R_1_ = CH_3_, R_F_ = C_5_F_11_) (Figure 1) are typical and will be described in details: the ^1^H- and ^13^C-NMR spectra contain six and ten distinct resonances, respectively (see 1D ^1^H and ^13^C-NMR in Supplementary Materials).

In its ^1^H-NMR spectrum, the singlet at δ_H_ 2.6 ppm (3H) is readily assigned to the methyl group which show a cross peak with the ^13^C resonance at δ_C_ 25 ppm in the ^1^H-^13^C HETCOR chart. Noticeably, upon shaking the samples dissolved in CDCl_3_ with one droplet of D_2_O, the methyl resonance of **17a** readily disappears from the ^1^H spectrum, denoting a rapid H/D exchange which is explained by the acidic character of the protons on alkyl substituents of the pyridinium ring.

^1^H resonances corresponding to the *N*-phenyl moiety, are divided into two groups: first a 3H multiplet is observed at δ_H_ 7.5–7.6 ppm corresponding to the H_m_ and H_p_ on the *meta*- and *para*- positions. Then, a slightly deshielded doublet resonance at δ_H_ 7.7 ppm corresponds to the two H_o_ protons at *ortho*- positions. On the HETCOR chart, the *meta*- and *para*-hydrogens resonances show cross peaks with ^13^C resonances at δ_C_ 130.5 and 132.5 ppm, while the ortho- proton resonance also show a clear cross peak with the ^13^C resonance at δ_C_ 126.7 ppm. 

Besides, the most deshielded resonances at δ_H_ 8.3, 8.7 and 9.1 ppm, were assigned to the three vicinal hydrogen atoms on the pyridinium nucleus (H3, H4 and H5) (Figure 1 and 1D ^1^H-NMR in Supporting Information). This can be confirmed in the ^1^H-^1^H COSY chart where clear cross peaks between those three resonances at δ_H_ 8.3, 8.7 and 9.1 ppm are observed. Moreover, those resonances exhibit *^3^J_H–H_* couplings constants of ca. 8–8.1 Hz characteristic of aromatic hydrogens. 

Among them, the most deshielded resonance (δ_H_ 9.1 ppm) is a triplet signal, thus corresponding to H4, due to vicinal couplings with two neighboring protons H3/H5 the resonances of which indeed appear as two doublets at δ_H_ 8.3 and 8.7 ppm, which correlate with ^13^C resonances at δ_C_ 128.8 and 136.2 ppm respectively on the HETCOR chart. The final assignment between H3 and H5 was made on basis of the presence of a 2.5 Hz triplet coupling on the 128.8-ppm ^13^C resonance explained as a *^3^J_CF_*coupling with fluorine nuclei on the vicinal CF_2_ group; thus both signals δ_H_ 8.3 ppm and δ_C_ 128.8 ppm belong to the H3/C3 position of the pyridinium ring, while the δ_H_ 8.7 ppm and δ_C_ 136.2 ppm corresponds to H5/C5.

Three additional ^13^C resonances in the aromatic shift range, correspond to quaternary carbons (invisible on DEPT spectra): the signal at δ_C_ 140.8 ppm appearing as a large triplet denotes a strong coupling with fluorine nuclei of vicinal CF_2_ group (*^2^J_CF_* = 26.3 Hz), thus can nonambiguously be attributed to carbon C2. Besides, on basis of a higher deshielding by the pyridinium ring, the two other resonances at δ_C_ 163.8 and 137.8 ppm, were assigned to the carbon C6 and the ipso carbon of the *N*-phenyl, respectively (Figure 1). 

Interestingly, we also observed a large deshielding of the ^19^F-NMR resonance of the α-CF_2_ directly attached to the pyridinium ring, probably because of the high electron-withdrawing effect of the adjacent positively charged nitrogen atom. This was similarly observed on the whole series of perfluoroalkyl-substituted pyridiniums **17**–**19**.

### 2.3. The m-Anisidine Case

**Scheme 5:**
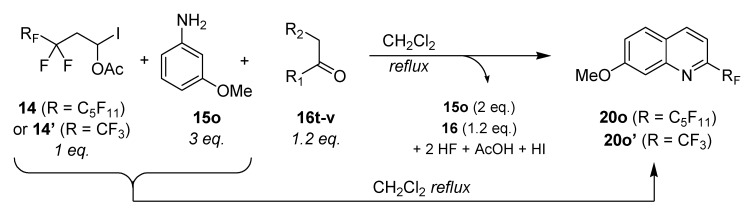
Formation of 2-perfluoroalkyl-7-methoxyquinolines **20o**–**o’** in the case of aniline **15o** (R = *m*-OMe), in the presence or absence of ketone **16**; examples in Table 4.

A notable exception to the above synthesis results, was observed however with a *meta*-methoxy substitution on substrate **15o** (*m*-anisidine) where the only observed and isolated product was the corresponding 2-perfluoroalkyl- or 2-trifluoromethyl-7-methoxyquinolines **20o** (R_F_ = C_5_F_11_) or **20o’** (R_F_ = CF_3_) in 80–90% yields (Scheme 5 and Table 4); while the ketone 16 was recovered at the end of the reaction (isolated and identified by NMR and mass spectrometry). In the absence of ketone 16 (namely upon reacting 14/14’ with three equivalents of **15o** under identical conditions) the same result was observed with similar yields of **20**, what is actually consistent with our previous investigations on such reactions [64]. 

### 2.4. Reactivity Investigation—Intermediate Isolation

Based on the observed reaction extents and required time for completion according to the substituents (Table 1, Table 2 and Table 3), this series of investigated examples provided preliminary insights on the reactivity of the system, where a primary factor of influence is likely the substitution of aniline **15**. For instance, the presence of an electron-withdrawing group on the aromatic ring of **15** resulted in longer reaction times being required to reach completion, as observed e.g., with R = Cl or CO_2_H at any *o*-, *m*-, *p*- position (Table 1, entries 12–14, 17–20; Table 2 entries 7, 9, 10; Table 3, entries 6–7, 10). An *ortho*-substituted aniline **15** also resulted in an extended reaction time, whenever the *o*-substituent was electron-donating or electron-withdrawing (R = *o*-Me, *o*-Et, *o*-OMe, *o*-Cl and *o*-CO_2_H; Table 1, entries 4, 8, 12, 15, 17–18; Table 2, entries 3, 9; Table 3, entries 8, 10).

In order to better understand the mechanism of this reaction, its evolution was monitored by ^19^F-NMR spectroscopy, carried out on aliquots of the reaction medium diluted in CDCl_3_ or DMSO-*d*_6_, then comparing the spectra of reaction mixtures with NMR data of compounds already investigated and characterized in the course of our previous studies on *gem*-iodoacetoxy related systems [59,60,61,62,63,64,65].

**Scheme 6:**
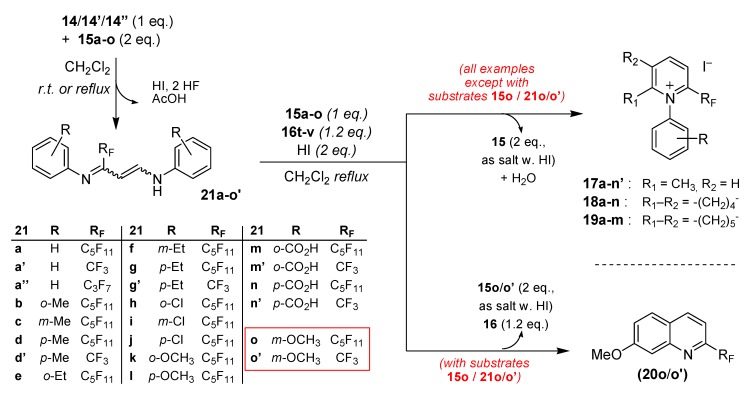
Synthesis of *N*,*N’*-diaryl-2-(perfluoroalkyl)-1,5-diazapentadienes intermediates **21a**–**o’**, and further formation of 2-trifluoromethyl and 2-perfluoroalkyl-*N*-arylpyridinium derivatives **17**–**19** or 2-perfluoroalkyl-7-methoxyquinoline **20**.

We thus observed that the consumption of the starting 1-acetoxy-1-iodo-2-(perfluoroalkyl)-ethanes **14**–**14”** was accompanied by the formation of an intermediate compound, the latter further disappeared while the final 2-perfluoroalkyl-*N*-arylpyridinium products **17**–**19** were gradually formed. Comparison with our previous NMR data provided fair assumption that such intermediate might be an *N*,*N’*-diaryl-2-(perfluoroalkyl)-1,5-diazapenta-1,3-diene **21** (Scheme 6), a few examples of which had previously been described by us [59,63,64]. 

In the absence of ketone **16** as reactant, and upon changing the other reactant stoichiometries to those stated in our previous works [64] namely 1 equiv. **14**–**14”** and 2 equiv. **15** (in DCM at rt: **21a**–**l,o**–**o’**; or refluxing: **21m–n’**), the main reaction product was the intermediate **21**, the latter (**21a**–**o’**) have been isolated in fair yields then characterized by NMR and mass spectrometry (Scheme 6). All *N*,*N’*-diaryl-2-(perfluoroalkyl)-1,5-diazapenta-1,3-dienes **21a**–**o’** were actually isolated as mixtures of *E/Z*-stereoisomers, as in our previous works [59,64,65]. 

### 2.5. Reactivity of Diazapentadienes ***21***

We then investigated the reactivity of diazapentadienes intermediates **21a**–**o’** in the presence of ketones **16a**–**c**, where we noticed that the presence of an additional amount of aniline **15** is necessary to achieve pyridinium **17**–**19** formation. Thus, upon reacting isolated intermediates **21** with 1.2 equivalents of ketone **16** and one equivalent of arylamine **15** (the same substitution as used in the formation of **21**) in the presence of hydrogen iodide in refluxing dichloromethane, the consumption of **21** (monitored by TLC and ^19^F-NMR spectroscopy) was accompanied with the formation of the corresponding 2-perfluoroalkyl-*N*-arylpyridiniums **17**–**19**. Noteworthy from a reactivity/mechanistic viewpoint, to our knowledge this is the first experimental evidence of the formation of arylpyridinium compounds from an *N*,*N’*-diaryl-1,5-diazapenta-1,3-diene intermediate.

In the *m*-methoxy case (substrate **15o**), while the derived diazapentadiene intermediate **21o**/**o’** was formed and isolated under identical conditions and with same yields as other examples a-n, the subsequent reaction of isolated **21o**/**o’** in the presence of ketone **16** under the same conditions (one equiv. **15o** under acidic conditions), formed the 2-perfluoroalkyl-7-methoxyquinolines **20o**/**o’** as the only products (Scheme 6, lower path), a result actually identical to the reaction of **21o**/**o’** with **15** in the absence of ketone [64]. 

**Scheme 7:**
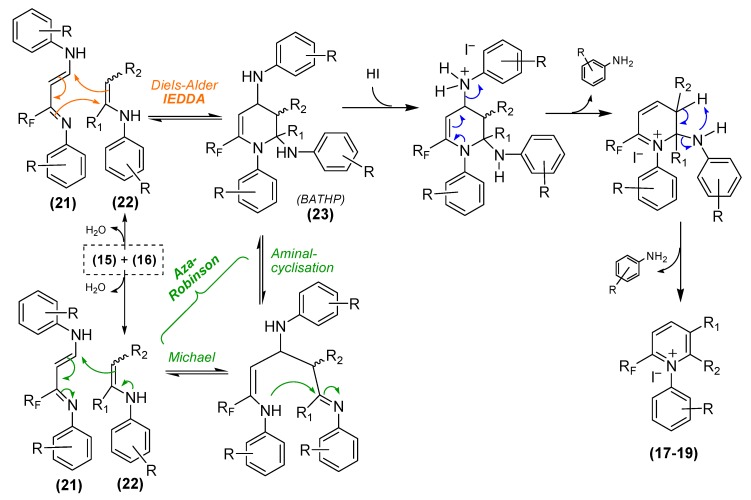
Diels-Alder/Aza-Robinson bis-de-anilino-elimination cascade mechanism hypotheses of formation 2-trifluoromethyl and 2-perfluoroalkyl-*N*-arylpyridiniums **17**–**19**. BATHP: bis-anilino-tetrahydropyridine.

Based on required reactant stoichiometry, the mechanism of the reactions may be rationalized as follows (Scheme 7). In a first step, the *gem*-iodoacetoxy substrate **14**/**14”** reacts with two equivalents of arylamine **15** to form the corresponding diazapentadiene **21**, according to our previously reported investigations [64]. Meanwhile, another equivalent of arylamine **15** may condense with ketone **16** through an acid catalyzed, addition-elimination mechanism to give a tautomeric *N*-phenyl-imino/enamine intermediate **22** (Scheme 7). Then, the resulting intermediates **21** and **22** may react together through two probably competing cyclization cascade mechanisms [66,67,68,69,70]: either: (*i*) an inverse electron demand Diels-Alder (IEDDA) reaction involving the cycloaddition of the diene **21** and the electron-rich dienophile **22**, or (*ii*) an aza-Robinson annulation-type reaction involving a Michaël addition of enamine **22** to diazapentadienes **21** then subsequent aminal-cyclisation. Both mechanisms could give a *bis*-anilinotetrahydropyridine (BATHP) intermediate **23**. The latter then undergoes a double de-anilino-elimination, thus yielding the corresponding *N*-arylpyridinium iodides **17**–**19** (Scheme 7).

### 2.6. Formation of 2-Perfluoroalkyl-7-Methoxyanilines ***20o–o’***

**Scheme 8:**
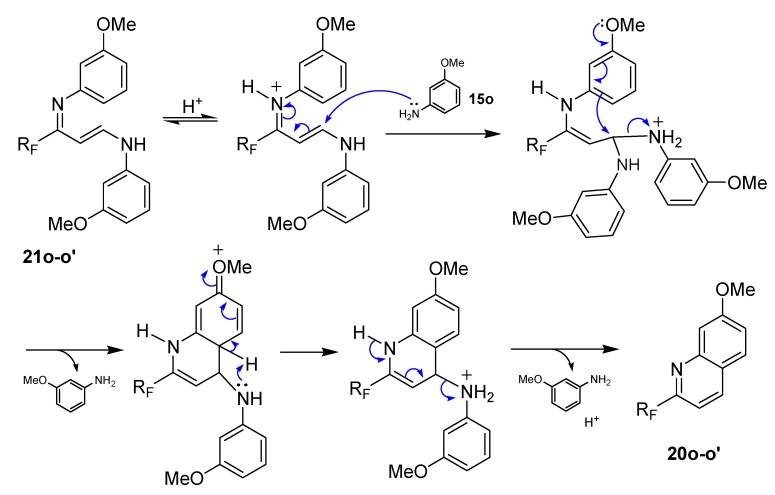
Proposed mechanism of formation of **20o**/**o’** from **21o**/**o’** [59,64].

The key step of this mechanism is the protonation of **21o** and the formation of a conjugated iminium intermediate (Scheme 8). Then Michael addition of *m*-methoxyaniline **15o** gives a cationic bis-anilino intermediate which undergo an intramolecular electrophilic aromatic cyclisation assisted by the electron-donating methoxyl group with subsequent elimination of a *m*-methoxyaniline. Then a prototropy induced by anilino-group followed by the loss of a proton and a second *m*-methoxyaniline result in the generation of an aromatic ring and the formation of 2-perfluoroalkyl-7-methoxyanilines **20o**–**o’**.

Conversely in the case of an *m*-methoxy substituted arylamine, the electron-donation ability of the methoxy- substituent on the *N*,*N’*-(3-methoxyphenyl)-2-(perfluoroalkyl)-1,5-diazapentadienes **21o**–**o’,** may promote their direct intramolecular cyclization to **20** (Scheme 8) [59,64], the latter route also taking advantage of an entropically favorable (unimolecular) cyclization over a bimolecular addition of **21o**–**o’** with **22o**.

Indeed, the literature [71] states that electronic and steric effects of substituents have very pronounced influence on both intermolecular Michaël 1,4-additions or Diels-Alder cycloadditions. Many studies show that stereoelectronic requirements for certain intermolecular reaction and the resulting increase in the potential energy for the corresponding transition states, could be so high that intramolecular cyclisation could take place instead [71]. In both cases anyway (either cycloaddition or cyclization), the subsequent elimination of two aniline molecules, may ensure irreversibility, again because of entropic effects.

## 3. Materials and Methods 

### 3.1. General Methods

All reaction solvents were purchased from commercial suppliers and distilled before use. All synthetic reactions were performed in oven-dried glassware, and their progress was monitored by thin layer chromatography (TLC) using silica gel plates, and by ^19^F-NMR spectroscopy of aliquots. Chromatographic column purifications were performed on silica gel (40–63 μm). ^1^H, ^13^C, and ^19^F-NMR spectra were recorded in either CDCl_3_ or DMSO-*d*_6_ solution on an Avance 300 (300 MHz) or an Avance 400 (400 MHz) spectrometer (Bruker, Billerica, MA, USA). Chemical shifts are reported in ppm, using the solvent signal chemical shift as a reference. Abbreviations used in the description of NMR spectra: s: singlet, d: doublet, t: triplet, q: quadruplet, bs: broad signal. Coupling constants (*J*) are given in Hertz. Mass spectra (either low- or high-resolution) were recorded on a SX102 mass spectrometer (JEOL, Tokyo, Japan) in FAB+ mode, using 3-nitrobenzyl alcohol matrix. Elemental analyses were carried out on a Flash 2000 elemental analyzer (Thermo Finnigan, Waltham, MA, USA).

### 3.2. General Procedure for the Synthesis 2-Trifluoromethylated and 2-Perfluoroalkylated 6-Methyl-N-(R-Phenyl)Pyridinium Iodides (***17a***–***l***), N-(R-Phenyl)-5,6,7,8-Tetrahydroquinolinium Iodides (***18a***–***l***) and N-(R-Phenyl)-6,7,8,9-Tetrahydro-5H-Cyclohepta[B]Pyridinium Iodides (***19a***–***l***) (All examples except R = CO_2_H or R = M-Ome)

To a stirred solution of 1-acetoxy-1-iodo-perfluoroalkylethane compounds **14** (1 equiv.) in anhydrous dichloromethane (10 mL DCM for 1g of **14**), was added 3 equiv. of the corresponding substituted aniline **15** and 1.2 equiv. of ketone **16**. The mixture was stirred under reflux for the desired time (4–12 h, Table 1, Table 2 and Table 3) until complete consumption of **14** (monitored by TLC eluent petroleum ether/ethyl acetate: 80/20 *v*/*v* and ^19^F-NMR spectroscopy of aliquots). When the reaction was completed, the mixture was allowed to cool to r.t. then the brown precipitate accumulated during the reaction was separated by vacuum filtration (it was subsequently identified as anilinium salts by NMR and MS). Then ethyl ether was added to the filtrate and the corresponding pyridinium iodides **17a**–**l, 18a**–**l**, and **19a**–**l** precipitate instantly and were isolated by vacuum filtration as amorphous solids.

*2-Perfluoropentyl-6-methyl-N-phenylpyridinium iodide* (**17a**). Aniline **15a** (5.24 g, 5.63 × 10^−2^ mole) and acetone (**16t**, 1.65 mL, 1.3 g, 2.25 × 10^−2^ mole) were added to a solution of **14** (R_F_ = C5F11, 10 g, 1.87 × 10^−2^ mole) in dry dichloromethane (100 mL). The mixture was stirred for 2 h at reflux to afford 9.55 g of the title product **17a**, total yield 90%. ^1^H-NMR (400.13 MHz, CDCl_3_) *δ* 2.6 (s, 3H, C*H*_3_), 7.5–7.6 (m, 3H, Ph-*H*), 7.7 (d, *J* = 7.1 Hz, 2H, Ph-*H*), 8.3 (d, *J* = 8 Hz, 1H, Py-*H*), 8.7 (d, *J* = 8 Hz, 1H, Py-*H*), 9.1 (t, *J* = 8.1 Hz, 1H, Py-*H*); ^1^H-NMR (400.13 MHz, CDCl_3_+D_2_O) *δ* 7.5–7.6 (m, 3H, Ph-*H*), 7.7 (d, *J* = 7.2 Hz, 2H, Ph-*H*), 8.3 (d, *J* = 8 Hz, 1H, Py-*H*), 8.7 (d, *J* = 8.1 Hz, 1H, Py-*H*), 9.1 (t, *J* = 8.1 Hz, 1H, Py-*H*); ^13^C-NMR (100.6 MHz, CDCl_3_) *δ* 25 (s, *C*H_3_), 126.7, 128.8 (t, *^3^J_CF_* = 2.5 Hz), 130.4, 132.5, 136.2, 137.8, 140.8 (t, *^2^J_CF_* = 26.3 Hz), 148, 163.8; ^19^F-NMR (235.3 MHz, CDCl_3_) *δ −*126.5 (m 2F, CF_2_-CF_2_-CF_2_-CF_2_-CF_3_), −123 (m 2F, CF_2_-CF_2_-CF_2_-CF_2_-CF_3_), −118 (m 2F, CF_2_-CF_2_-CF_2_-CF_2_-CF_3_), −102.5 (m 2F, CF_2_-CF_2_-CF_2_-CF_2_-CF_3_), −81.5 (m 3F, CF_2_-CF_2_-CF_2_-CF_2_-CF_3_). MS (*m*/*z*): 438 ([M − I]^+^, 100). HRMS calcd. for C_17_H_11_F_11_N^+^ 438.0716, found 438.0720. Anal. Calcd. for C_17_H_11_F_11_NI: C, 36.13; H, 1.96; N, 2.48. Found: C, 36.15; H, 1.95; N, 2.46.

*2-Trifluoromethyl-6-methyl-N-phenylpyridinium iodide* (**17a’**). Aniline **15a** (8.4 g, 9 × 10^−2^ mol) and acetone (**16t**, 2.7 mL, 2 g, 3.61 × 10^−2^ mol) were added to a solution of **14’** (R_F_ = CF3, 10 g, 3 × 10^−2^ mol) in dry dichloromethane (100 mL). The mixture was stirred for 2 h at reflux to give 9.9 g of the title product **17a’**, total yield 90%. Spectral data: ^1^H-NMR (300.13 MHz, CDCl_3_) *δ* 2.6 (s, 3H, CH_3_), 7.5–7.6 (m, 3H), 7.8 (d, *J* = 9 Hz, 2H), 8.5 (dd, *J* = 9 and 3 Hz, 1H), 8.6 (dd, *J* = 9 and 3 Hz, 1H), 9 (t, *J* = 9 Hz, 1H); ^13^C-NMR (75.46 MHz, CDCl_3_) *δ* 24.1 (s, CH_3_), 118.6 (q, CF_3_, *^1^J_CF_* = 276.9 Hz), 125.6 (q, *^3^J_CF_* = 5.2 Hz), 125.7, 130.1, 132.3, 135.1, 136.7, 140.9 (q, C-CF_3_, *^2^J_CF_* = 35.4 Hz), 148, 162.5; ^19^F-NMR (282.4 MHz, CDCl_3_) *δ* −59.8 (s 3F, CF_3_). MS (*m*/*z*): 238 ([M − I]^+^, 100). HRMS calcd. for C_13_H_11_F_3_N^+^: 238.0844, found 238.0848. Anal. Calcd. for C_13_H_11_F_3_NI: C, 42.76; H, 3.04; N, 3.84. Found: C, 42.78; H, 3.05; N, 3.82.

*2-Perfluoropropyl-6-methyl-N-phenylpyridinium iodide (***17a’’**). Aniline **15a** (6.45 g, 6.94 × 10^−2^ mol) and acetone (**16t**, 1.6 mL, 2 g, 2.77 × 10^−2^ mol) were added to a solution of **14’’** (R_F_ = C3F7, 10 g, 2.31 × 10^−2^ mol) in dry dichloromethane (100 mL). The mixture was stirred for 2 h at reflux, affording 9.69 g of the title product **17a’’**, total yield 91%. Spectral data: ^1^H-NMR (400.13 MHz, CDCl_3_) *δ* 2.6 (s, 3H, CH_3_), 7.5–7.6 (m, 3H), 7.7 (d, *J* = 7 Hz, 2H), 8.3 (d, *J* = 8.1 Hz, 1H), 8.8 (d, *J* = 8 Hz, 1H), 9 (t, *J* = 8 Hz, 1H); ^13^C-NMR (100.6 MHz, CDCl_3_) *δ* 25.1 (s, CH_3_), 126.5, 128.7 (t, *^3^J_CF_* = 2.5 Hz), 130.4, 132.2, 136.1, 137.7, 140.9 (t, *^2^J_CF_* = 26.5 Hz), 148, 163.7; ^19^F-NMR (235.3 MHz, CDCl_3_) *δ* −127 (m 2F, CF_2_), −102 (m 2F, CF_2_), −80.5 (m 3F, CF_3_). MS (*m*/*z*): 338 ([M − I]^+^, 100). HRMS calcd. for C_15_H_11_F_7_N^+^: 338.0780, found 338.0782. Anal. Calcd. for C_15_H_11_F_7_NI: C, 38.73; H, 2.38; N, 3.01. Found: C, 38.75; H, 2.39; N, 3.04. 

*2-Perfluoropentyl-6-methyl-N-(2-methylphenyl)pyridinium iodide* (**17b**). 2-Methylaniline (**15b,** 6 g, 5.63 × 10^−2^ mol) and acetone (**16t**, 1.66 mL, 1.3 g, 2.25 × 10^−2^ mol) were added to a solution of **14** (R_F_ = C_5_F_11,_ 10 g, 1.87 × 10^−2^ mol) in dry dichloromethane (100 mL). The mixture was stirred for 4 h at reflux, to give 8.7 g of the title product **17b**, total yield 80%. Spectral data: ^1^H-NMR (400.13 MHz, CDCl_3_) *δ* 2.5 (s, 3H, CH_3_), 2.6 (s, 3H, CH_3_), 7.4–7.6 (m, 4H), 7.9 (t, *J* = 7.2 Hz, 1H), 8.2 (d, *J* = 8.1 Hz, 1H), 8.9 (d, *J* = 8.2 Hz, 1H); ^13^C-NMR (100.6 MHz, CDCl_3_) *δ* 20.1 (s, CH_3_), 25 (s, CH_3_), 127, 128.5 (t, *^3^J_CF_* = 2 Hz), 130.5, 131.9, 136.1, 138, 141.1 (t, *^2^J_CF_* = 26 Hz), 148, 163.5; ^19^F-NMR (282.4 MHz, CDCl_3_) *δ* −125.7 (m 2F, CF_2_), −122.1 (m 2F, CF_2_), −118.2 (AB system, *^2^J_FF_* = 338.8 Hz, 1F, CF_2_-CF_2_-CF_2_), −116 (AB system, *^2^J_FF_* = 338.8 Hz, 1F, CF_2_-CF_2_-CF_2_), −108.5 (AB system, *^2^J_FF_* = 282.5 Hz, 1F, CF_2_-CF_2_-CF_2_), −98 (AB system, *^2^J_FF_* = 282.5 Hz, 1F, CF_2_-CF_2_-CF_2_), −80.6 (m 3F, CF_3_). MS (*m*/*z*): 452 ([M − I]^+^, 100). HRMS calcd. for C_18_H_13_F_11_N^+^: 452.0872, found 452.0877. Anal. Calcd. for C_18_H_13_F_11_NI: C, 37.33; H, 2.26; N, 2.42. Found: C, 37.35; H, 2.30; N, 2.40.

*2-Perfluoropentyl-6-methyl-N-(3-methylphenyl)pyridinium iodide* (**17c**). 3-Methylaniline (**15c**, 6.34 g, 5.92 × 10^−2^ mol) and acetone (**16t**, 1.75 mL, 1.37 g, 2.36 × 10^−2^ mol) were added to a solution of **14** (R_F_ = C5F11, 0.5 g, 1.97 × 10^−2^ mol) in dry dichloromethane (105 mL). The mixture was stirred for 4 h at reflux to give 9.7 g of the title product **17c**, total yield 85%. Spectral data: ^1^H-NMR (400.13 MHz, CDCl_3_) *δ* 2.6 (s, 3H, CH_3_), 2.7 (s, 3H, CH_3_), 7.4–7.6 (m, 4H), 8.3 (d, *J* = 7.2 Hz, 1H), 8.8 (d, *J* = 8.1 Hz, 1H), 9.2 (t, *J* = 8.1 Hz, 1H); ^13^C-NMR (100.6 MHz, CDCl_3_) *δ* 22 (s, CH_3_), 26 (s, CH_3_), 121, 123, 128 (t, *^3^J_CF_* = 2 Hz), 130.5, 132, 136.1, 137, 139 (t, *^2^J_CF_* = 25 Hz), 145, 148, 161; ^19^F-NMR (282.4 MHz, CDCl_3_) *δ* −126 (s 2F, CF_2_), −122.4 (s 2F, CF_2_), −117.2 (s 2F, CF_2_), −103.5 (AB system, *^2^J_FF_* = 310.6 Hz, 1F, CF_2_-CF_2_), −101.1 (AB system, *^2^J_FF_* = 310.6 Hz, 1F, CF_2_-CF_2_), −80.7 (m 3F, CF_3_). MS (*m*/*z*): 452 ([M − I]^+^, 95). HRMS calcd. for C_18_H_13_F_11_N^+^: 452.0872, found 452.0875. Anal. Calcd. for C_18_H_13_F_11_NI: C, 37.33; H, 2.26; N, 2.42. Found: C, 37.36; H, 2.28; N, 2.40.

*2-Perfluoropentyl-6-methyl-N-(4-methylphenyl)pyridinium iodide* (**17d**). 4-Methylaniline (**15d**, 5.74 g, 5.35 × 10^−2^ mol) and acetone (**16t,** 1.58 mL, 1.24 g, 2.14 × 10^−2^ mol) were added to a solution of **14** (R_F_ = C5F11, 9.5 g, 1.78 × 10^−2^ mol) in dry dichloromethane (95 mL). The mixture was stirred for 4 h at reflux to afford 8.79 g of the title product **17d**, total yield 85%. Spectral data: ^1^H-NMR (400.13 MHz, CDCl_3_) *δ* 2.65 (s, 3H, CH_3_), 2.7 (s, 3H, CH_3_), 7.4 (d, *J* = 8 Hz, 2H), 7.7 (d, *J* = 8 Hz, 2H), 8.3 (d, *J* = 8.2 Hz, 1H), 8.8 (d, *J* = 8.3 Hz, 1H), 9 (t, *J* = 8.2 Hz, 1H); ^13^C-NMR (100.6 MHz, CDCl_3_) *δ* 25 (s, CH_3_), 26.1 (s, CH_3_), 122.7, 126.5, 127.7 (t, *^3^J_CF_* = 1.5 Hz), 129.9, 130, 135.1, 137.8 (t, *^2^J_CF_* = 27.2 Hz), 142.5, 146.6, 150.2, 167.5; ^19^F-NMR (282.4 MHz, CDCl_3_) *δ −*126 (s 2F, CF_2_), −122.4 (s 2F, CF_2_), −117.2 (s 2F, CF_2_), −101.6 (s, 2F, CF_2_-CF_2_), −80.7 (m 3F, CF_3_). MS (*m*/*z*): 452 ([M − I]^+^, 100). HRMS calcd. for C_18_H_13_F_11_N^+^: 452.0872, found 452.0878. Anal. Calcd. for C_18_H_13_F_11_NI: C, 37.33; H, 2.26; N, 2.42. Found: C, 37.37; H, 2.26; N, 2.39. 

*2-Trifluoromethyl-6-methyl-N-(4-methylphenyl)pyridinium iodide* (**17d’**). 4-Methylaniline (**15d**, 8.81 g, 8.22 × 10^−2^ mol) and acetone (**16t**, 2.43 mL, 1.9 g, 3.28 × 10^−2^ mol) were added to a solution of **14’** (R_F_ = CF3, 9.1 g, 2.74 × 10^−2^ mol) in dry dichloromethane (91 mL). The mixture was stirred for 4 h at reflux to yield 9.14 g of the title product **17d’**, total yield 88%. Spectral data: ^1^H-NMR (400.13 MHz, CDCl_3_) *δ* 2.6 (s, 3H, CH_3_), 2.7 (s, 3H, CH_3_), 7.4 (d, *J* = 8.1 Hz, 2H), 7.7 (d, *J* = 8 Hz, 2H), 8.3 (d, *J* = 8 Hz, 1H), 8.7 (d, *J* = 8.1 Hz, 1H), 9.2 (t, *J* = 8.1 Hz, 1H); ^13^C-NMR (75.46 MHz, CDCl_3_) *δ* 24.2 (s, CH_3_), 25 (s, CH_3_), 118.4 (q, CF_3_, *^1^J_CF_* = 276.8 Hz), 124.8 (q, *^3^J_CF_* = 5 Hz), 122.5, 125.6, 130, 132.3, 140.9 (q, C-CF_3_, *^2^J_CF_* = 36 Hz), 147.2, 150, 162.5; ^19^F-NMR (282.4 MHz, CDCl_3_) *δ* −59.9 (s 3F, CF_3_). MS (*m*/*z*): 252 ([M − I]^+^, 95). HRMS calcd. for C_14_H_13_F_3_N^+^: 252.100, found 252.1010. Anal. Calcd. for C_14_H_13_F_3_NI: C, 44.35; H, 3.46; N, 3.69. Found: C, 44.38; H, 3.47; N, 3.70. 

*2-Perfluoropentyl-6-methyl-N-(2-ethylphenyl)pyridinium iodide* (**17e**). 2-Ethylaniline (**15e**, 7 g, 5.8 × 10^−2^ mol) and acetone (**16t**, 1.7 mL, 1.34 g, 2.32 × 10^−2^ mol) were added to a solution of **14** (R_F_ = C_5_F_11_, 10.3 g, 1.93 × 10^−2^ mol) in dry dichloromethane (103 mL). The mixture was stirred for 6 h at reflux to produce 8 g of the title product **17e**, total yield 70%. Spectral data: ^1^H-NMR (400.13 MHz, CDCl_3_) *δ* 1.3 (t, *J* = 8 Hz, 3H, CH_2_-CH_3_), 2.6 (q, *J* = 8 Hz, 2H, CH_2_-CH_3_), 2.8 (s, 3H, CH_3_), 7.4–7.6 (m, 3H), 8 (s, 1H), 8.2 (d, *J* = 8 Hz, 1H), 8.9 (d, *J* = 8.1 Hz, 1H); 9.2 (t, *J* = 8.1 Hz, 1H); ^13^C-NMR (100.6 MHz, CDCl_3_) *δ* 13.2 (s, CH_2_-CH_3_), 24.3 (s, CH_2_-CH_3_), 27.1 (s, CH_3_), 124.2, 125.9, 127.8 (t, *^3^J_CF_* = 2.1 Hz), 129.6, 131, 136.1, 137.5 (t, *^2^J_CF_* = 25 Hz), 146.5, 150.1, 167.7; ^19^F-NMR (282.4 MHz, CDCl_3_) *δ −*126 (m 2F, CF_2_), −122.9 (AB system, *^2^J_FF_* = 289.6 Hz, 1F, CF_2_-CF_2_-CF_2_-CF_2_-CF_3_), −121.8 (AB system, *^2^J_FF_* = 289.6 Hz, 1F, CF_2_-CF_2_-CF_2_-CF_2_-CF_3_), −118.7 (AB system, *^2^J_FF_* = 300.9 Hz, 1F, CF_2_-CF_2_-CF_2_-CF_2_-CF_3_), −115.9 (AB system, *^2^J_FF_* = 300.9 Hz, 1F, CF_2_-CF_2_-CF_2_-CF_2_-CF_3_), −108.5 (AB system, *^2^J_FF_* = 289.6 Hz, 1F, CF_2_-CF_2_-CF_2_-CF_2_-CF_3_), −98.8 (AB system, *^2^J_FF_* = 289.6 Hz, 1F, CF_2_-CF_2_-CF_2_-CF_2_-CF_3_), −80.7 (m 3F, CF_3_). MS (*m*/*z*): 466 ([M − I]^+^, 95). HRMS calcd. for C_19_H_15_F_11_N^+^: 466.1029, found 466.1033. Anal. Calcd. for C_19_H_15_F_11_NI: C, 38.47; H, 2.55; N, 2.36. Found: C, 38.48; H, 2.56; N, 2.40. 

*2-Perfluoropentyl-6-methyl-N-(3-ethylphenyl)pyridinium iodide* (**17f**). 3-Ethylaniline (**15f**, 7.17 g, 5.92 × 10^−2^ mol) and acetone (**16t**, 1.75 mL, 1.37 g, 2.36 × 10^−2^ mol) were added to a solution of **14** (R_F_ = C5F11, 10.5 g, 1.97 × 10^−2^ mol) in dry dichloromethane (105 mL). The mixture was stirred for 4 h at reflux to obtain. 9.36 g of the title product **17f**, total yield 80%. Spectral data: ^1^H-NMR (300.13 MHz, CDCl_3_) *δ* 1.3 (t, *J* = 8 Hz, 3H, CH_2_-CH_3_), 2.7 (s, 3H, CH_3_), 2.8 (q, *J* = 8 Hz, 2H, CH_2_-CH_3_), 7.4–7.7 (m, 4H), 8.4 (d, *J* = 6.7 Hz, 1H), 8.9 (d, *J* = 7.1 Hz, 1H), 9.2 (t, *J* = 7.9 Hz, 1H); ^13^C-NMR (75.46 MHz, CDCl_3_) *δ* 13 (s, CH_2_-CH_3_), 23 (s, CH_2_-CH_3_), 27 (s, CH_3_), 121.5, 123.5, 127 (t), 128, 130, 134, 135, 139 (t, *^2^J_CF_* = 25 Hz), 145, 146, 162; ^19^F-NMR (282.4 MHz, CDCl_3_) *δ* −126 (s 2F, CF_2_), −122.4 (s 2F, CF_2_), −117.2 (s 2F, CF_2_), −102.5 (AB system, *^2^J_FF_* = 315.5 Hz, 1F, CF_2_-CF_2_), −101.5 (AB system, *^2^J_FF_* = 315.5 Hz, 1F, CF_2_-CF_2_), −80.7 (m 3F, CF_3_). MS (*m*/*z*): 466 ([M − I]^+^, 100). HRMS calcd. for C_19_H_15_F_11_N^+^: 466.1029, found 466.1035. Anal. Calcd. for C_19_H_15_F_11_NI: C, 38.47; H, 2.55; N, 2.36. Found: C, 38.49; H, 2.56; N, 2.33. 

*2-Perfluoropentyl-6-methyl-N-(4-ethylphenyl)pyridinium iodide* (**17g**). 6.9 g of 4-ethylaniline **15g** (5.69 × 10^−2^ mol) and 1.68 mL or 1.32 g (2.27 × 10^−2^ mol) of acetone **16t** were added to a solution of 10.1 g (1.89 × 10^−2^ mol) of **14** (R_F_ = C_5_F_11_) in 101 mL of dry dichloromethane. The mixture was stirred for 4 h at reflux. 9.34 g of the title product **17g** were obtained, total yield 83%. Spectral data: ^1^H-NMR (300.13 MHz, CDCl_3_) *δ* 1.3 (t, *J* = 8 Hz, 3H, CH_2_-CH_3_), 2.7 (s, 3H, CH_3_), 2.8 (q, *J* = 8 Hz, 2H, CH_2_-CH_3_), 7.1 (d, *J* = 8.4 Hz, 2H), 7.7 (d, *J* = 8.3 Hz, 2H), 8.4 (d, *J* = 7.5 Hz, 1H), 8.8 (d, *J* = 7.8 Hz, 1H), 9.1 (t, *J* = 7.8 Hz, 1H); ^13^C-NMR (75.46 MHz, CDCl_3_) *δ* 13 (s, CH_2_-CH_3_), 24 (s, CH_2_-CH_3_), 26.8 (s, CH_3_), 113.5, 126.5, 127.5 (t, *^3^J_CF_* = 2 Hz), 129.5, 130, 135, 139 (t, *^2^J_CF_* = 25 Hz), 147, 159, 162; ^19^F-NMR (282.4 MHz, CDCl_3_) *δ −*126.9 (s 2F, CF_2_), −123.3 (s 2F, CF_2_), −117.3 (s 2F, CF_2_), −101.8 (s, 2F, CF_2_-CF_2_), −80.7 (m 3F, CF_3_). MS (*m*/*z*): 466 ([M − I]^+^, 100). HRMS calcd. for C_19_H_15_F_11_N^+^: 466.1029, found 466.1030. Anal. Calcd. for C_19_H_15_F_11_NI: C, 38.47; H, 2.55; N, 2.36. Found: C, 38.48; H, 2.56; N, 2.35. 

*2-Trifluoromethyl-6-methyl-N-(4-ethylphenyl)pyridinium iodide (***17g’***).* 9.85 g of 4-ethylaniline **15g** (8.13 × 10^−2^ mol) and 2.4 mL or 1.88 g (3.25 × 10^−2^ mol) of acetone **16t** were added to a solution of 9 g (2.71 × 10^−2^ mol) of **14’** (R_F_ = CF_3_) in 90 mL of dry dichloromethane. The mixture was stirred for 4 h at reflux. 9 g of the title product **17g’** were obtained, total yield 85%. Spectral data: ^1^H-NMR (400.13 MHz, CDCl_3_) *δ* 1.3 (t, *J* = 8 Hz, 3H, CH_2_-CH_3_), 2.7 (s, 3H, CH_3_), 2.8 (q, *J* = 8 Hz, 2H, CH_2_-CH_3_), 7.1 (d, *J* = 8 Hz, 2H), 7.7 (d, *J* = 8 Hz, 2H), 8.2 (d, *J* = 8 Hz, 1H), 8.7 (d, *J* = 8 Hz, 1H), 9 (t, *J* = 8.1 Hz, 1H); ^13^C-NMR (75.46 MHz, CDCl_3_) *δ* 13.2 (s, CH_2_-CH_3_), 23.9 (s, CH_2_-CH_3_), 27 (s, CH_3_), 118.1 (q, CF_3_, *^1^J_CF_* = 277.6 Hz), 125 (q, *^3^J_CF_* = 4.8 Hz), 122.4, 125.7, 130.2, 132, 147.2 (q, C-CF_3_, *^2^J_CF_* = 35.5 Hz), 149.9, 162; ^19^F-NMR (282.4 MHz, CDCl_3_) *δ −*58.9 (s 3F, CF_3_). MS (*m*/*z*): 266 ([M − I]^+^, 100). HRMS calcd. for C_15_H_15_F_3_N^+^: 266.1157, found 266.1160. Anal. Calcd. for C_15_H_15_F_3_NI: C, 45.82; H, 3.85; N, 3.56. Found: C, 45.85; H, 3.86; N, 3.52. 

*2-Perfluoropentyl-6-methyl-N-(2-chlorophenyl)pyridinium iodide (***17h***)*. 7.4 g of 2-chloroaniline **15h** (5.8 × 10^−2^ mol) and 1.7 mL or 1.34 g (2.32 × 10^−2^ mol) of acetone **16t** were added to a solution of 10.3 g (1.93 × 10^−2^ mol) of **14** (R_F_ = C_5_F_11_) in 103 mL of dry dichloromethane. The mixture was stirred for 12 h at reflux. 6.96 g of the title product **17h** were obtained, total yield 60%. Spectral data: ^1^H-NMR (400.13 MHz, CDCl_3_) *δ* 2.8 (s, 3H, CH_3_), 7.7–7.9 (m, 3H), 8.4 (m, 1H), 8.5 (d, *J* = 8 Hz, 1H), 8.9 (d, *J* = 8.1 Hz, 1H); 9.4 (t, *J* = 8 Hz, 1H); ^13^C-NMR (100.6 MHz, CDCl_3_) *δ* 24.2 (s, CH_3_), 128.5, 129.1, 130.1, 130.8, 133.8, 135, 136.2, 140.5 (t, *^2^J_CF_* = 24.1 Hz), 148.8, 163.8; ^19^F-NMR (282.4 MHz, CDCl_3_) *δ −*126.5 (AB system, *^2^J_FF_* = 289 Hz, 1F, CF_2_-CF_2_-CF_2_-CF_2_-CF_3_), −125.7 (AB system, *^2^J_FF_* = 289 Hz, 1F, CF_2_-CF_2_-CF_2_-CF_2_-CF_3_), −122.9 (AB system, *^2^J_FF_* = 289.6 Hz, 1F, CF_2_-CF_2_-CF_2_-CF_2_-CF_3_), −121.6 (AB system, *^2^J_FF_* = 289.6 Hz, 1F, CF_2_-CF_2_-CF_2_-CF_2_-CF_3_), −118.7 (AB system, *^2^J_FF_* = 299.5 Hz, 1F, CF_2_-CF_2_-CF_2_-CF_2_-CF_3_), −115.9 (AB system, *^2^J_FF_* = 299.5 Hz, 1F, CF_2_-CF_2_-CF_2_-CF_2_-CF_3_), −108.2 (AB system, *^2^J_FF_* = 288.6 Hz, 1F, CF_2_-CF_2_-CF_2_-CF_2_-CF_3_), −98.8 (AB system, *^2^J_FF_* = 288.6 Hz, 1F, CF_2_-CF_2_-CF_2_-CF_2_-CF_3_), −80.7 (m 3F, CF_3_). MS (*m*/*z*): 472 ([M − I]^+^, 90). HRMS calcd. for C_17_H_10_ClF_11_N^+^: 472.0326, found 472.0329. Anal. Calcd. for C_17_H_10_ClF_11_NI: C, 34.05; H, 1.68; N, 2.34. Found: C, 34.08; H, 1.67; N, 2.33. 

*2-Perfluoropentyl-6-methyl-N-(3-chlorophenyl)pyridinium iodide (***17i***).* 7.55 g of 3-chloroaniline **15i** (5.92 × 10^−2^ mol) and 1.75 mL or 1.37 g (2.36 × 10^−2^ mol) of acetone **16t** were added to a solution of 10.5 g (1.97 × 10^−2^ mol) of **14** (R_F_ = C_5_F_11_) in 105 mL of dry dichloromethane. The mixture was stirred for 6 h at reflux. 8.28 g of the title product **17i** were obtained, total yield 70%. Spectral data: ^1^H-NMR (400.13 MHz, CDCl_3_) *δ* 2.7 (s, 3H, CH_3_), 7.3 (s, 1H), 7.4–7.6 (m, 2H), 7.7 (m, 1H), 8.3 (d, *J* = 8 Hz, 1H), 8.5 (d, *J* = 8 Hz, 1H), 8.8 (t, *J* = 8 Hz, 1H); ^13^C-NMR (100.6 MHz, CDCl_3_) *δ* 27.3 (s, CH_3_), 127.9, 129.6, 131.4, 134.1, 135.5, 138.5 (t, *^2^J_CF_* = 25 Hz), 141.1, 150.6, 166.4; ^19^F-NMR (282.4 MHz, CDCl_3_) *δ −*121.5 (s 2F, CF_2_), −117.5 (s 2F, CF_2_), −112.7 (s 2F, CF_2_), −97.4 (AB system, *^2^J_FF_* = 105.3 Hz, 1F, CF_2_-CF_2_), −97.1 (AB system, *^2^J_FF_* = 105.3 Hz, 1F, CF_2_-CF_2_), −75.9 (m 3F, CF_3_). MS (*m*/*z*): 472 ([M − I]^+^, 90). HRMS calcd. for C_17_H_10_ClF_11_N^+^: 472.0326, found 472.0330. Anal. Calcd. for C_17_H_10_ClF_11_NI: C, 34.05; H, 1.68; N, 2.34. Found: C, 34.11; H, 1.65; N, 2.36.

*2-Perfluoropentyl-6-methyl-N-(4-chlorophenyl)pyridinium iodide (***17j***).* 7.19 g of 4-chloroaniline **15j** (5.63 × 10^−2^ mol) and 1.66 mL or 1.3 g (2.25 × 10^−2^ mol) of acetone **16t** were added to a solution of 10 g (1.87 × 10^−2^ mol) of **14** (R_F_ = C_5_F_11_) in 100 mL of dry dichloromethane. The mixture was stirred for 6 h at reflux. 9.58 g of the title product **17j** were obtained, total yield 85%. Spectral data: ^1^H-NMR (300.13 MHz, CDCl_3_) *δ* 2.5 (s, 3H, CH_3_), 7.9 (d, *J* = 8 Hz, 2H), 8.2 (d, *J* = 8.1 Hz, 2H), 8.6 (d, *J* = 8 Hz, 1H), 8.8 (d, *J* = 7.9 Hz, 1H), 9 (t, *J* = 7.9 Hz, 1H); ^13^C-NMR (75.46 MHz, CDCl_3_) *δ* 26 (s, CH_3_), 113.2, 126, 127.4 (t, *^3^J_CF_* = 2.2 Hz), 130.2, 136, 139.2 (t, *^2^J_CF_* = 24.8 Hz), 147.2, 158, 162.3; ^19^F-NMR (282.4 MHz, CDCl_3_) *δ −*125.8 (s 2F, CF_2_), −122.3 (s 2F, CF_2_), −117.7 (s 2F, CF_2_), −101.8 (s, 2F, CF_2_-CF_2_), −80.2 (m 3F, CF_3_); MS (*m*/*z*): 472 ([M − I]^+^, 90). HRMS calcd. for C_17_H_10_ClF_11_N^+^: 472.0326, found 472.0324. Anal. Calcd. for C_17_H_10_ClF_11_NI: C, 34.05; H, 1.68; N, 2.34. Found: C, 34.10; H, 1.68; N, 2.35.

*2-Perfluoropentyl-6-methyl-N-(2-methoxyphenyl)pyridinium iodide (***17k***).* 6.8 g of 2-methoxyaniline **15k** (5.52 × 10^−2^ mol) and 1.63 mL or 1.28 g (2.21 × 10^−2^ mol) of acetone **16t** were added to a solution of 9.8 g (1.84 × 10^−2^ mol) of **14** (R_F_ = C_5_F_11_) in 98 mL of dry dichloromethane. The mixture was stirred for 6 h at reflux. 7.67 g of the title product **17k** were obtained, total yield 70%. Spectral data: ^1^H-NMR (300.13 MHz, CDCl_3_) *δ* 2.8 (s, 3H, CH_3_), 3.7 (s, 3H, OCH_3_), 7.1 (d, *J* = 8.3 Hz, 1H), 7.2 (t, *J* = 7.6 Hz, 1H), 7.7 (t, *J* = 8.8 Hz, 1H), 8 (m, 1H), 8.3 (d, *J* = 8.1 Hz, 1H), 8.8 (d, *J* = 8.2 Hz, 1H), 9 (d, *J* = 8.2 Hz, 1H); ^13^C-NMR (75.46 MHz, CDCl_3_) *δ* 22.2 (s, CH_3_), 54 (s, OCH_3_), 113.1, 128.5, 129.1, 130.8, 133.7, 136.2, 139.9 (t, *^2^J_CF_* = 25 Hz), 147, 160, 162.2; ^19^F-NMR (282.4 MHz, CDCl_3_) *δ −*126.5 (AB system, *^2^J_FF_* = 295 Hz, 1F, CF_2_-CF_2_-CF_2_-CF_2_-CF_3_), −125.5 (AB system, *^2^J_FF_* = 295 Hz, 1F, CF_2_-CF_2_-CF_2_-CF_2_-CF_3_), −122.9 (AB system, *^2^J_FF_* = 289 Hz, 1F, CF_2_-CF_2_-CF_2_-CF_2_-CF_3_), −121.5 (AB system, *^2^J_FF_* = 289 Hz, 1F, CF_2_-CF_2_-CF_2_-CF_2_-CF_3_), −119 (AB system, *^2^J_FF_* = 288 Hz, 1F, CF_2_-CF_2_-CF_2_-CF_2_-CF_3_), −116.5 (AB system, *^2^J_FF_* = 288 Hz, 1F, CF_2_-CF_2_-CF_2_-CF_2_-CF_3_), −107 (AB system, *^2^J_FF_* = 282.5 Hz, 1F, CF_2_-CF_2_-CF_2_-CF_2_-CF_3_), −99.5 (AB system, *^2^J_FF_* = 282.5 Hz, 1F, CF_2_-CF_2_-CF_2_-CF_2_-CF_3_), −80.69 (m 3F, CF_3_). MS (*m*/*z*): 468 ([M − I]^+^, 100). HRMS calcd. for C_18_H_13_F_11_NO^+^: 468.0821, found 468.0827. Anal. Calcd. for C_18_H_13_F_11_INO: C, 36.32; H, 2.20; N, 2.35. Found: C, 36.35; H, 2.21; N, 2.34.

*2-Perfluoropentyl-6-methyl-N-(4-methoxyphenyl)pyridinium iodide (***17l***).* 6.94 g of 4-methoxyaniline **15l** (5.63 × 10^−2^ mol) and 1.66 mL or 1.3 g (2.25 × 10^−2^ mol) of acetone **16t** were added to a solution of 10 g (1.87 × 10^−2^ mol) of **14** (R_F_ = C_5_F_11_) in 100 mL of dry dichloromethane. The mixture was stirred for 4 h at reflux. 9.62 g of the title product **17l** were obtained, total yield 86%. Spectral data: ^1^H-NMR (300.13 MHz, CDCl_3_) *δ* 2.7 (s, 3H, CH_3_), 3.9 (s, 3H, OC*H*_3_), 7.2 (d, *J* = 8.5 Hz, 2H), 7.7 (d, *J* = 8.4 Hz, 2H), 8.4 (d, *J* = 7.5 Hz, 1H), 8.8 (d, *J* = 7.8 Hz, 1H), 9.1 (t, *J* = 7.8 Hz, 1H); ^13^C-NMR (75.46 MHz, CDCl_3_) *δ* 22.3 (s, CH_3_), 54.2 (s, OCH_3_), 113, 128.7 (t, *^3^J_CF_* = 2 Hz), 129.4, 130.5, 133, 136.1, 140 (t, *^2^J_CF_* = 25 Hz), 147, 160.2, 162.5; ^19^F-NMR (282.4 MHz, CDCl_3_) *δ −*126.1 (s 2F, CF_2_), −122.3 (s 2F, CF_2_), −117.1 (s 2F, CF_2_), −101.8 (s, 2F, CF_2_-CF_2_), −80.7 (m 3F, CF_3_). MS (*m*/*z*): 468 ([M − I]^+^, 100). HRMS calcd. for C_18_H_13_F_11_NO^+^: 468.0821, found 468.0830. Anal. Calcd. for C_18_H_13_F_11_INO: C, 36.32; H, 2.20; N, 2.35. Found: C, 36.39; H, 2.21; N, 2.38.

*2-Perfluoropentyl-N-(phenyl)-5,6,7,8-tetrahydroquinolinium iodide (***18a***).* 5.24 g of aniline **15a** (5.63 × 10^−2^ mole) and 2.21 g (2.25 × 10^−2^ mole) of cyclohexanone **16u** were added to a solution of 10 g (1.87 × 10^−2^ mole) of **14** (R_F_ = C_5_F_11_) in 50 mL of dry dichloromethane. The mixture was stirred for 4 h at reflux. 10 g of the title product **18a** were obtained, total yield 88%. Spectral data: ^1^H-NMR (300.13 MHz, CDCl_3_) *δ* 2 (m, 4H), 2.7 (m, 2H), 3.3 (m, 2H), 7.5–7.6 (m, 3H), 7.7 (d, *J* = 7.1 Hz, 2H), 8.2 (d, *J* = 8.1 Hz, 1H), 8.7 (d, *J* = 8.2 Hz, 1H); ^13^C-NMR (75.46MHz, CDCl_3_) *δ* 19.5, 21, 29.5, 31.2, 115, 125.1, 127, 128.1, 129.2, 139 (t, *^2^J_CF_* = 27 Hz), 146, 148.1, 163.1; ^19^F-NMR (235.3 MHz, CDCl_3_) *δ −*126.2 (m 2F, CF_2_), −122.4 (m 2F, CF_2_), −117.3 (m 2F, CF_2_), −101.8 (m 2F, CF_2_), −80.7 (m 3F, CF_3_). MS (*m*/*z*): 478 ([M − I]^+^, 95). HRMS calcd. for C_20_H_15_F_11_N^+^ 478.1029, found 478.1033. Anal. Calcd. for C_20_H_15_F_11_NI: C, 39.69; H, 2.50; N, 2.31. Found: C, 39.72; H, 2.51; N, 2.29. 

*2-Trifluoromethyl-N-(phenyl)-5,6,7,8-tetrahydroquinolinium iodide (***18a’***).* 7.14 g of aniline **15a** (7.68 × 10^−2^ mol) and 3 g (3.07 × 10^−2^ mol) of cyclohexanone **16u** were added to a solution of 8.5 g (2.56 × 10^−2^ mol) of **14’** (R_F_ = CF_3_) in 85 mL of dry dichloromethane. The mixture was stirred for 4 h at reflux. 9.23 g of the title product **18a’** were obtained, total yield 89%. ^1^H-NMR (300.13 MHz, CDCl_3_) *δ* 1.9 (m, 4H, CH_2_, cyclohexyl group), 2.6 (m, 2H, CH_2_, cyclohexyl group), 3.3 (m, 2H, CH_2_, cyclohexyl group), 7.5–7.6 (m, 3H, Ph-*H*), 7.8 (d, *J* = 7.5 Hz, 2H, Ph-*H*), 8.2 (d, *J* = 8 Hz, 1H, Py-*H*), 8.8 (d, *J* = 8.1 Hz, 1H, Py-*H*); ^13^C-NMR (75.46 MHz, CDCl_3_) *δ* 19.8, 21, 29.6, 32.2, 115, 118.6 (q, *C*F_3_, *^1^J_CF_* = 282.5 Hz), 125.2, 127.1, 128.8, 129.2, 139.5, 146.4, 148.5 (q, *C*-CF_3_, *^2^J_CF_* = 34.5 Hz), 163; ^19^F-NMR (282.4 MHz, DMSO-*d*_6_) *δ −*59.8 (s 3F, CF_3_). MS (*m*/*z*): 278 ([M − I]^+^, 95). HRMS calcd. for C_16_H_15_F_3_N^+^: 278.1157, found 278.1160. Anal. Calcd. for C_16_H_15_F_3_NI: C, 47.43; H, 3.73; N, 3.46. Found: C, 47.45; H, 3.74; N, 3.44.

*2-Perfluoropentyl-N-(2-methylphenyl)-5,6,7,8-tetrahydroquinolinium iodide (***18b***).* 5.86 g of 2-methylaniline **15b** (5.46 × 10^−2^ mol) and 2.14 g (2.18 × 10^−2^ mol) of cyclohexanone **16u** were added to a solution of 9.7 g (1.82 × 10^−2^ mol) of **14** (R_F_ = C_5_F_11_) in 97 mL of dry dichloromethane. The mixture was stirred for 6 h at reflux. 8.12 g of the title product **18b** were obtained, total yield 72%. Spectral data: ^1^H-NMR (300.13 MHz, CDCl_3_) *δ* 1.8 (bs, 2H), 1.9 (s, 3H, CH_3_), 2.1 (m, 3H), 2.9 (m, 1H), 3.3 (m, 1H), 3.4 (m, 1H), 7.3–7.5 (m, 3 H), 8 (t, *J* = 7.2 Hz, 1H), 8.2 (d, *J* = 8.1 Hz, 1H), 8.8 (d, *J* = 8.1 Hz, 1H); ^13^C-NMR (75.46MHz, CDCl_3_) *δ* 19.9, 21, 26, 29.5, 32.1, 115.2, 126.5, 127.8, 128, 129.5, 136.5, 138 (t, *^2^J_CF_* = 24.2 Hz), 148.1, 150.5, 161.1, 163; ^19^F-NMR (282.4 MHz, DMSO-*d*_6_) *δ −*126.3 (AB system, *^2^J_FF_* = 285.6 Hz, 1F, CF_2_-CF_2_-CF_2_-CF_2_-CF_3_), −125.4 (AB system, *^2^J_FF_* = 285.6 Hz, 1F, CF_2_-CF_2_-CF_2_-CF_2_-CF_3_), −123.1 (AB system, *^2^J_FF_* = 310 Hz, 1F, CF_2_-CF_2_-CF_2_-CF_2_-CF_3_), −121.7 (AB system, *^2^J_FF_* = 310 Hz, 1F, CF_2_-CF_2_-CF_2_-CF_2_-CF_3_), −118.7 (AB system, *^2^J_FF_* = 299.6 Hz, 1F, CF_2_-CF_2_-CF_2_-CF_2_-CF_3_), −117.6 (AB system, *^2^J_FF_* = 299.6 Hz, 1F, CF_2_-CF_2_-CF_2_-CF_2_-CF_3_), −107.1 (AB system, *^2^J_FF_* = 290.5 Hz, 1F, CF_2_-CF_2_-CF_2_-CF_2_-CF_3_), −98.2 (AB system, *^2^J_FF_* = 290.5 Hz, 1F, CF_2_-CF_2_-CF_2_-CF_2_-CF_3_), −80.2 (m 3F, CF_3_). MS (*m*/*z*): 492 ([M − I]^+^, 100). HRMS calcd. for C_21_H_17_F_11_N^+^: 492.1185, found 492.1190. Anal. Calcd. for C_21_H_17_F_11_NI: C, 40.73; H, 2.77; N, 2.26. Found: C, 40.75; H, 2.76; N, 2.25.

*2-Perfluoropentyl-N-(3-methylphenyl)-5,6,7,8-tetrahydroquinolinium iodide (***18c***).* 6.4 g of 3-methylaniline **15c** (5.97 × 10^−2^ mol) and 2.34 g (2.39 × 10^−2^ mol) of cyclohexanone **16u** were added to a solution of 10.6 g (1.99 × 10^−2^ mol) of **14** (R_F_ = C_5_F_11_) in 53 mL of dry dichloromethane. The mixture was stirred for 4 h at reflux. 10.2 g of the title product **18c** were obtained, total yield 83%. Spectral data: ^1^H-NMR (300.13 MHz, CDCl_3_) *δ* 1.7 (m, 2H), 1.9 (m, 2H), 2.1 (s, 3H, CH_3_), 2.9 (m, 2H), 3.5 (m, 2H), 7.5 (s, 1H), 7.6 (m, 3H), 8.2 (d, *J* = 8.1 Hz, 1H), 8.7 (d, *J* = 8.1 Hz, 1H); ^13^C-NMR (75.46MHz, CDCl_3_) *δ* 19.8, 21.1, 26.3, 29.5, 31, 117.2, 126.1, 127.8, 128.1, 129.2, 136.8, 138.5 (t, *^2^J_CF_* = 25.8 Hz), 148.2, 150.5, 163.1; ^19^F-NMR (235.3 MHz, CDCl_3_) *δ −*126.1 (m 2F, CF_2_), −122 (m 2F, CF_2_), −117 (m 2F, CF_2_), −103.1 (AB system, *^2^J_FF_* = 275.6 Hz, 1F, CF_2_-CF_2_), −101.2 (AB system, *^2^J_FF_* = 275.6 Hz, 1F, CF_2_-CF_2_), −80.1 (m 3F, CF_3_). MS (*m*/*z*): 492 ([M − I]^+^, 100). HRMS calcd. for C_21_H_17_F_11_N^+^: 492.1185, found 492.1187. Anal. Calcd. for C_21_H_17_F_11_NI: C, 40.73; H, 2.77; N, 2.26. Found: C, 40.76; H, 2.77; N, 2.27.

*2-Perfluoropentyl-N-(4-methylphenyl)-5,6,7,8-tetrahydroquinolinium iodide (***18d***).* 5.74 g of 4-methylaniline **15d** (5.35 × 10^−2^ mol) and 2.1 g (2.14 × 10^−2^ mol) of cyclohexanone **16u** were added to a solution of 9.5 g (1.78 × 10^−2^ mol) of **14** (R_F_ = C_5_F_11_) in 95 mL of dry dichloromethane. The mixture was stirred for 4 h at reflux. 8.84 g of the title product **18d** were obtained, total yield 80%. Spectral data: ^1^H-NMR (300.13 MHz, CDCl_3_) *δ* 1.9 (m, 4H), 2 (s, 3H, CH_3_), 2.5 (m, 2H), 3.4 (m, 2H), 7.4 (d, *J* = 8 Hz, 2H), 7.7 (d, *J* = 8.1 Hz, 2H), 8.2 (d, *J* = 8.1 Hz, 1H), 8.7 (d, *J* = 8.1 Hz, 1H); ^13^C-NMR (75.46MHz, CDCl_3_) *δ* 19.5, 21, 26.2, 29.5, 31.2, 116.2, 125.1, 127, 128.1, 129.2, 135.1, 137.8 (t, *^2^J_CF_* = 26 Hz), 146, 148.1, 150, 163.1; ^19^F-NMR (235.3 MHz, CDCl_3_) *δ −*126 (m 2F, CF_2_), −122.5 (m 2F, CF_2_), −117.5 (m 2F, CF_2_), −101.2 (m 2F, CF_2_), −80.6 (m 3F, CF_3_). MS (*m*/*z*): 492 ([M − I]^+^, 100). HRMS calcd. for C_21_H_17_F_11_N^+^: 492.1185, found 492.1189. Anal. Calcd. for C_21_H_17_F_11_NI: C, 40.73; H, 2.77; N, 2.26. Found: C, 40.75; H, 2.78; N, 2.25. 

*2-Perfluoropentyl-N-(4-ethylphenyl)-5,6,7,8-tetrahydroquinolinium iodide (***18g***).* 7.24 g of 4-ethylaniline **15g** (5.97 × 10^−2^ mol) and 2.34 g (2.39 × 10^−2^ mol) of cyclohexanone **16u** were added to a solution of 10.6 g (1.99 × 10^−2^ mol) of **14** (R_F_ = C_5_F_11_) in 106 mL of dry dichloromethane. The mixture was stirred for 4 h at reflux. 10.72 g of the title product **18g** were obtained, total yield 85%. Spectral data: ^1^H-NMR (300.13 MHz, CDCl_3_) *δ* 1.3 (t, *J* = 8 Hz, 3H, CH_2_-CH_3_), 1.9 (m, 4H), 2.6 (m, 2H), 2.8 (q, *J* = 8 Hz, 2H, CH_2_-CH_3_), 3.3 (m, 2H), 7.1 (d, *J* = 8.2 Hz, 2H), 7.7 (d, *J* = 8.3 Hz, 2H), 8.2 (d, *J* = 8.1 Hz, 1H), 8.6 (d, *J* = 8.1 Hz, 1H); ^13^C-NMR (75.46MHz, CDCl_3_) *δ* 13.1, 19.5, 21, 24.1, 29.5, 31.2, 113.2, 126.1, 127, 129.6, 129.8, 135.1, 138.8 (t, *^2^J_CF_* = 26.2 Hz), 148.1, 152, 162.9; ^19^F-NMR (235.3 MHz, CDCl_3_) *δ −*126.9 (m 2F, CF_2_), −123.5 (m 2F, CF_2_), −117.5 (m 2F, CF_2_), −101.8 (m 2F, CF_2_), −80.6 (m 3F, CF_3_). MS (*m*/*z*): 506 ([M − I]^+^, 95). HRMS calcd. for C_22_H_19_F_11_N^+^: 506.1342, found 506.1346. Anal. Calcd. for C_22_H_19_F_11_NI: C, 41.72; H, 3.02; N, 2.21. Found: C, 41.75; H, 3.04; N, 2.19. 

*2-Perfluoropentyl-N-(3-chlorophenyl)-5,6,7,8-tetrahydroquinolinium iodide (***18i***).* 7.33 g of 3-chloroaniline **15i** (5.75 × 10^−2^ mol) and 2.25 g (2.3 × 10^−2^ mol) of cyclohexanone **16u** were added to a solution of 10.2 g (1.91 × 10^−2^ mol) of **14** (R_F_ = C_5_F_11_) in 102 mL of dry dichloromethane. The mixture was stirred for 4 h at reflux. 9.32 g of the title product **18i** were obtained, total yield 76%. Spectral data: ^1^H-NMR (300.13 MHz, CDCl_3_) *δ* 1.7 (m, 2H), 1.9 (m, 2H), 2.9 (m, 2H), 3.5 (m, 2H), 7.4 (s, 1H), 7.5–7.6 (m, 2H), 7.7 (m, 1H), 8.2 (d, *J* = 8 Hz, 1H), 8.7 (d, *J* = 8 Hz, 1H); ^13^C-NMR (75.46MHz, CDCl_3_) *δ* 19.6, 21, 26, 29.5, 31.5, 118.2, 127.1, 127.8, 128.1, 130.1, 137.8, 138.8 (t, *^2^J_CF_* = 24.8 Hz), 147.2, 150.6, 165.6; ^19^F-NMR (235.3 MHz, CDCl_3_) *δ −*125.6 (m 2F, CF_2_), −117.6 (m 2F, CF_2_), −113.5 (m 2F, CF_2_), −97.9 (AB system, *^2^J_FF_* = 150.2 Hz, 1F, CF_2_-CF_2_), −98.2 (AB system, *^2^J_FF_* = 150.2 Hz, 1F, CF_2_-CF_2_), −79.1 (m 3F, CF_3_). MS (*m*/*z*): 512 ([M − I]^+^, 90). HRMS calcd. for C_20_H_14_F_11_NCl^+^: 512.0639, found 512.0643. Anal. Calcd. for C_20_H_14_F_11_NICl: C, 37.55; H, 2.21; N, 2.19. Found: C, 37.58; H, 2.22; N, 2.17. 

*2-Perfluoropentyl-N-(4-methoxyphenyl)-5,6,7,8-tetrahydroquinolinium iodide (***18l***).* 7.36 g of 4-anisidine **15l** (5.97 × 10^−2^ mol) and 2.34 g (2.39 × 10^−2^ mol) of cyclohexanone **16u** were added to a solution of 10.6 g (1.99 × 10^−2^ mol) of **14** (R_F_ = C_5_F_11_) in 106 mL of dry dichloromethane. The mixture was stirred for 4 h at reflux. 11.39 g of the title product **18l** were obtained, total yield 90%. Spectral data: ^1^H-NMR (300.13 MHz, CDCl_3_) *δ* 2 (m, 4H), 2.7 (m, 2H), 3.3 (m, 2H), 3.9 (s, 3H, OCH_3_), 7.1 (d, *J* = 8.8 Hz, 2H), 7.7 (d, *J* = 8.6 Hz, 2H), 8.2 (d, *J* = 8.1 Hz, 1H), 8.8 (d, *J* = 8.2 Hz, 1H); ^13^C-NMR (75.46MHz, CDCl_3_) *δ* 20.1, 22, 29, 31.1, 56, 117, 125, 127, 128.2, 129.9, 139.5 (t, *^2^J_CF_* = 27.2 Hz), 146.2, 148.1, 162, 164.5; ^19^F-NMR (235.3 MHz, CDCl_3_) *δ −*126.9 (m 2F, CF_2_), −123.5 (m 2F, CF_2_), −117.5 (m 2F, CF_2_), −101.7 (m 2F, CF_2_), −80.75 (m 3F, CF_3_). MS (*m*/*z*): 508 ([M − I]^+^, 100). HRMS calcd. for C_21_H_17_F_11_NO^+^: 508.1134, found 508.1138. Anal. Calcd. for C_21_H_17_F_11_NIO: C, 39.70; H, 2.70; N, 2.20. Found: C, 39.72; H, 2.71; N, 2.19. 

*2-Perfluoropentyl-N-(phenyl)-6,7,8,9-tetrahydro-5H-cyclohepta[b]pyridinium (***19a***).* 5.24 g of aniline **15a** (5.63 × 10^−2^ mole) and 2.52 g (2.25 × 10^−2^ mole) of cycloheptanone **16v** were added to a solution of 10 g (1.87 × 10^−2^ mole) of **14** (R_F_ = C_5_F_11_) in 100 mL of dry dichloromethane. The mixture was stirred for 4 h at reflux. 9.54 g of the title product **19a** were obtained, total yield 82%. ^1^H-NMR (300.13 MHz, CDCl_3_) *δ* 1.7 (m, 2H, CH_2_, cycloheptyl group), 1.9 (m, 4H, CH_2_, cycloheptyl group), 3.1 (m, 2H, CH_2_, cycloheptyl group), 3.4 (m, 2H, CH_2_, cycloheptyl group), 7.5–7.7 (m, 5H, Ph-*H*), 8.2 (d, *J* = 8.2 Hz, 1H, Py-*H*), 8.9 (d, *J* = 8 Hz, 1H, Py-*H*); ^13^C-NMR (75.46MHz, CDCl_3_) *δ* 24, 25.3, 30.7, 33.9, 35.4, 124.2, 125.9, 127.7, 129.6, 131.5, 137.7 (t, *^2^J_CF_* = 27.1 Hz), 146.8, 150.3, 167.4; ^19^F-NMR (282.4 MHz, CDCl_3_) *δ −*126 (m 2F, CF_2_-CF_2_ CF_2_ CF_2_-CF_3_), −122.4 (m 2F, CF_2_-CF_2_-CF_2_-CF_2_-CF_3_), −117.3 (m 2F, CF_2_-CF_2_-CF_2_-CF_2_-CF_3_), −101.6 (m 2F, CF_2_-CF_2_-CF_2_-CF_2_-CF_3_), −80.7 (m 3F, CF_2_-CF_2_-CF_2_ CF_2_-CF_3_). MS (*m*/*z*): 492 ([M − I]^+^, 95). HRMS calcd. for C_21_H_17_F_11_N^+^ 492.1185, found 492.1190. Anal. Calcd. for C_21_H_17_F_11_NI: C, 40.73; H, 2.77; N, 2.26. Found: C, 40.75; H, 2.78; N, 2.25.

*2-Perfluoropentyl-N-(3-methylphenyl)-6,7,8,9-tetrahydro-5H-cyclohepta[b]pyridinium (***19c***).* 5.92 g of 3-methylaniline **15c** (5.52 × 10^−2^ mol) and 2.47 g (2.21 × 10^−2^ mol) of cycloheptanone **16v** were added to a solution of 9.8 g (1.84 × 10^−2^ mol) of **14** (R_F_ = C_5_F_11_) in 98 mL of dry dichloromethane. The mixture was stirred for 4 h at reflux. 9.68 g of the title product **19c** were obtained, total yield 83%. Spectral data: ^1^H-NMR (300.13 MHz, CDCl_3_) *δ* 1.7 (m, 2H), 1.9 (m, 4H), 2.45 (s, 3H, CH_3_), 3 (d, *J* = 8 Hz, 2H), 3.4 (m, 2H), 7.4 (m, 2H), 7.6 (m, 2H), 8.2 (d, *J* = 8.4 Hz, 1H), 8.9 (d, *J* = 8 Hz, 1H); ^13^C-NMR (75.46MHz, CDCl_3_) *δ* 20.7, 21.5, 24.3, 25.3, 30.7, 33.9, 35.7, 122.5, 126.2, 127.7, 129.5, 131, 137.6 (t, *^2^J_CF_* = 27 Hz), 146.6, 150.2, 167.8; ^19^F-NMR (235.3 MHz, CDCl_3_) *δ −*126 (m 2F, CF_2_), −122.5 (m 2F, CF_2_), −117 (m 2F, CF_2_), −101.7 (m 2F, CF_2_), −80.7 (m 3F, CF_3_). MS (*m*/*z*): 506 ([M − I]^+^, 95). HRMS calcd. for C_22_H_19_F_11_N^+^: 506.1342, found 506.1346. Anal. Calcd. for C_22_H_19_F_11_NI: C, 41.72; H, 3.02; N, 2.21. Found: C, 41.75; H, 3.06; N, 2.19.

*2-Perfluoropentyl-N-(4-methylphenyl)-6,7,8,9-tetrahydro-5H-cyclohepta[b]pyridinium (***19d***).* 6.34 g of 4-methylaniline **15d** (5.92 × 10^−2^ mol) and 2.65 g (2.36 × 10^−2^ mol) of cycloheptanone **16v** were added to a solution of 10.5 g (1.97 × 10^−2^ mol) of **14** (R_F_ = C_5_F_11_) in 105 mL of dry dichloromethane. The mixture was stirred for 4 h at reflux. 10.62 g of the title product **19d** were obtained, total yield 85%. Spectral data: ^1^H-NMR (300.13 MHz, CDCl_3_) *δ* 1.6 (m, 2H), 1.9 (m, 4H), 2.4 (s, 3H, CH_3_), 3 (m, 2H), 3.3 (m, 2H), 7.3 (d, *J* = 8 Hz, 2H), 7.6 (d, *J* = 8 Hz, 2H), 8.2 (d, *J* = 8.4 Hz, 1H), 8.8 (d, *J* = 8.4 Hz, 1H); ^13^C-NMR (75.46MHz, CDCl_3_) *δ* 20.8, 21.3, 24.2, 25.3, 30.6, 33.9, 35.4, 122.7, 126.6, 127.7, 129.9, 130.2, 135.1, 137.8 (t, *^2^J_CF_* = 27.1 Hz), 142.5, 146.6, 150.2, 167.7; ^19^F-NMR (235.3 MHz, CDCl_3_) *δ −*126 (m 2F, CF_2_), −122.4 (m 2F, CF_2_), −117.2 (m 2F, CF_2_), −101.6 (m 2F, CF_2_), −80.7 (m 3F, CF_3_). MS (*m*/*z*): 506 ([M − I]^+^, 95). HRMS calcd. for C_22_H_19_F_11_N^+^: 506.1342, found 506.1348. Anal. Calcd. for C_22_H_19_F_11_NI: C, 41.72; H, 3.02; N, 2.21. Found: C, 41.76; H, 3.05; N, 2.23. 

*2-Perfluoropentyl-N-(3-ethylphenyl)-6,7,8,9-tetrahydro-5H-cyclohepta[b]pyridinium (***19f***).* 6.69 g of 3-ethylaniline **15f** (5.52 × 10^−2^ mol) and 2.47 g (2.21 × 10^−2^ mol) of cycloheptanone **16v** were added to a solution of 9.8 g (1.84 × 10^−2^ mol) of **14** (R_F_ = C_5_F_11_) in 98 mL of dry dichloromethane. The mixture was stirred for 4 h at reflux. 10.5 g of the title product **19f** were obtained, total yield 88%. Spectral data: ^1^H-NMR (300.13 MHz, CDCl_3_) *δ* 1.2 (t, *J* = 8 Hz, 3H, CH_2_-CH_3_), 1.6 (s, 2H), 1.9 (m, 4H), 2.7 (q, *J* = 8 Hz, 2H, CH_2_-CH_3_), 3 (m, 2H), 3.3 (m, 2H), 7.5 (m, 3 H), 7.6 (m, 1H), 8.2 (d, *J* = 8.4 Hz, 1H), 8.9 (d, *J* = 8 Hz, 1H); ^13^C-NMR (75.46MHz, CDCl_3_) *δ* 15.1, 24.3, 25.4, 28.4, 30.6, 33.9, 35.4, 124.1, 125.9, 127.7, 129.6, 131.5, 137.6 (t, *^2^J_CF_* = 26.7 Hz), 146.7, 150.2, 167.4; ^19^F-NMR (235.3 MHz, CDCl_3_) *δ −*126 (m 2F, CF_2_), −122.4 (m 2F, CF_2_), −117.2 (m 2F, CF_2_), −102.2 (AB system, *^2^J_FF_* = 263.3 Hz, 1F, CF_2_-CF_2_-CF_2_-CF_2_-CF_3_), −101.1 (AB system, *^2^J_FF_* = 263.3 Hz, 1F, CF_2_-CF_2_-CF_2_-CF_2_-CF_3_), −80.7 (m 3F, CF_3_). MS (*m*/*z*): 520 ([M − I]^+^, 100). HRMS calcd. for C_23_H_21_F_11_N^+^: 520.1498, found 520.1501. Anal. Calcd. for C_23_H_21_F_11_NI: C, 42.68; H, 3.27; N, 2.16. Found: C, 42.70; H, 3.28; N, 2.14.

*2-Perfluoropentyl-N-(4-ethylphenyl)-6,7,8,9-tetrahydro-5H-cyclohepta[b]pyridinium (***19g***).* 6.96 g of 4-ethylaniline **15g** (5.75 × 10^−2^ mol) and 2.58 g (2.3 × 10^−2^ mol) of cycloheptanone **16v** were added to a solution of 10.2 g (1.91 × 10^−2^ mol) of **14** (R_F_ = C_5_F_11_) in 102 mL of dry dichloromethane. The mixture was stirred for 4 h at reflux. 10.3 g of the title product **19g** were obtained, total yield 83%. Spectral data: ^1^H-NMR (300.13 MHz, CDCl_3_) *δ* 1.3 (t, *J* = 7.6 Hz, 3H, CH_2_-CH_3_), 1.6 (s, 2H), 1.8 (m, 4H), 2.71 (q, *J* = 7.6 Hz, 2H, CH_2_-CH_3_), 3 (m, 2H), 3.4 (m, 2H), 7.3 (d, *J* = 8.4 Hz, 2H), 7.6 (d, *J* = 8.4 Hz, 2H), 8.1 (d, *J* = 8 Hz, 1H), 8.8 (d, *J* = 8 Hz, 1H); ^13^C-NMR (75.46MHz, CDCl_3_) *δ* 14.9, 24.2, 25.3, 28.5, 30.6, 33.9, 35.4, 126.7, 127.7, 128.9, 135.2, 137.8 (t, *^2^J_CF_* = 27.1 Hz), 146.6, 148.6, 150.2, 167.7; ^19^F-NMR (235.3 MHz, CDCl_3_) *δ −*126.1 (m 2F, CF_2_), −122.5 (m 2F, CF_2_), −117.2 (m 2F, CF_2_), −101.6 (m 2F, CF_2_), −80.8 (m 3F, CF_3_). MS (*m*/*z*): 520 ([M − I]^+^, 100). HRMS calcd. for C_23_H_21_F_11_N^+^: 520.1498, found 520.1499. Anal. Calcd. for C_23_H_21_F_11_NI: C, 42.68; H, 3.27; N, 2.16. Found: C, 42.69; H, 3.29; N, 2.15. 

*2-Perfluoropentyl-N-(3-chlorophenyl)-6,7,8,9-tetrahydro-5H-cyclohepta[b]pyridinium (***19i***).* 6.4 g of 3-chloroaniline **15i** (5.01 × 10^−2^ mol) and 2.25 g (2.10^−2^ mol) of cycloheptanone **16v** were added to a solution of 8.9 g (1.67 × 10^−2^ mol) of **14** (R_F_ = C_5_F_11_) in 89 mL of dry dichloromethane. The mixture was stirred for 4 h at reflux. 7.65 g of the title product **19i** were obtained, total yield 70%. Spectral data: ^1^H-NMR (300.13 MHz, CDCl_3_) *δ* 1.6–2.2 (m, 6H), 3.1 (m, 2H), 3.4 (s, 2H), 7.6 (t, *J* = 8.1 Hz, 1H), 7.7 (d, *J* = 8.2 Hz, 1H), 7.9 (s, 1H), 8.1 (d, *J* = 7.9 Hz, 1H), 8.3 (d, *J* = 8.1 Hz, 1H), 8.9 (d, *J* = 8.2 Hz, 1H); ^13^C-NMR (75.46MHz, CDCl_3_) *δ* 24.1, 25.2, 30.7, 34, 34.3, 35.5, 127.9, 128.5, 129.8, 136.1, 137.7 (t, *^2^J_CF_* = 26.8 Hz), 138.5, 146.8, 150.5, 167.7; ^19^F-NMR (235.3 MHz, CDCl_3_) *δ −*126 (m 2F, CF_2_), −122.4 (m 2F, CF_2_), −117.2 (m 2F, CF_2_), −101.5 (m 2F, CF_2_), −80.7 (m 3F, CF_3_). MS (*m*/*z*): 526 ([M − I]^+^, 100). HRMS calcd. for C_21_H_16_F_11_NCl^+^: 526.0796, found 526.0799. Anal. Calcd. for C_21_H_16_F_11_NICl: C, 38.58; H, 2.47; N, 2.14. Found: C, 38.61; H, 2.48; N, 2.14.

*2-Perfluoropentyl-N-(4-chlorophenyl)-6,7,8,9-tetrahydro-5H-cyclohepta[b]pyridinium (***19j***).* 7.26 g of 4-chloroaniline **15j** (5.69 × 10^−2^ mol) and 2.55 g (2.27 × 10^−2^ mol) of cycloheptanone **16v** were added to a solution of 10.1 g (1.89 × 10^−2^ mol) of **14** (R_F_ = C_5_F_11_) in 101 mL of dry dichloromethane. The mixture was stirred for 4 h at reflux. 9.68 g of the title product **19j** were obtained, total yield 78%. Spectral data: ^1^H-NMR (300.13 MHz, CDCl_3_) *δ* 1.7 (m, 2H), 1.9 (m, 4H), 3 (m, 2H), 3.3 (m, 2H), 7.5 (d, *J* = 8.8 Hz, 2H), 7.8 (d, *J* = 8.8 Hz, 2H), 8.2 (d, *J* = 8 Hz, 1H), 8.8 (d, *J* = 8.3 Hz, 1H); ^13^C-NMR (75.46MHz, CDCl_3_) *δ* 24, 25.2, 30.7, 33.9, 34.2, 35.4, 127.9, 128.4, 129.9, 136.1, 137.5 (t, *^2^J_CF_* = 26.8 Hz), 138.3, 146.8, 150.5, 167.6; ^19^F-NMR (235.3 MHz, CDCl_3_) *δ −*126 (m 2F, CF_2_), −122.3 (m 2F, CF_2_), −117.3 (m 2F, CF_2_), −101.5 (m 2F, CF_2_), −80.7 (m 3F, CF_3_). MS (*m*/*z*): 526 ([M − I]^+^, 100). HRMS calcd. for C_21_H_16_F_11_NCl^+^: 526.0796, found 526.0797. Anal. Calcd. for C_21_H_16_F_11_NICl: C, 38.58; H, 2.47; N, 2.14. Found: C, 38.60; H, 2.47; N, 2.13. 

*2-Perfluoropentyl-N-(2-methoxyphenyl)-6,7,8,9-tetrahydro-5H-cyclohepta[b]pyridinium (***19k***).* 6.18 g of 2-anisidine **15k** (5.01 × 10^−2^ mol) and 2.25 g (2 × 10^−2^ mol) of cycloheptanone **16v** were added to a solution of 8.9 g (1.67 × 10^−2^ mol) of **14** (R_F_ = C_5_F_11_) in 45 mL of dry dichloromethane. The mixture was stirred for 6 h at reflux. 7.6 g of the title product **19k** were obtained, total yield 70%. Spectral data: ^1^H-NMR (300.13 MHz, CDCl_3_) *δ* 1.5 (m, 1H), 1.7–2 (m, 4H), 2.1 (m, 1H), 2.9 (m,1H), 3.2–3.5 (m, 3H), 3.8 (s, 3H, OCH_3_), 7.2 (d, *J* = 8.3 Hz, 1H), 7.3 (t, *J* = 7.6 Hz, 1H), 7.7 (t, *J* = 8.8 Hz, 1H), 8 (s, 1H), 8.3 (d, *J* = 8.1 Hz, 1H), 9 (d, *J* = 8.2 Hz, 1H); ^13^C-NMR (75.46MHz, CDCl_3_) *δ* 24, 25.1, 30.7, 34.4, 34.3, 35.6, 127.8, 128.5, 130, 136.1, 137.8 (t, *^2^J_CF_* = 26.9 Hz), 138.8, 147, 151.5, 167.5; ^19^F-NMR (282.4 MHz, DMSO-*d*_6_) *δ −*126.5 (AB system, *^2^J_FF_* = 284.5 Hz, 1F, CF_2_-CF_2_-CF_2_-CF_2_-CF_3_), −125.5 (AB system, *^2^J_FF_* = 284.5 Hz, 1F, CF_2_-CF_2_-CF_2_-CF_2_-CF_3_), −123 (AB system, *^2^J_FF_* = 355.2 Hz, 1F, CF_2_-CF_2_-CF_2_-CF_2_-CF_3_), −121.8 (AB system, *^2^J_FF_* = 355.2 Hz, 1F, CF_2_-CF_2_-CF_2_-CF_2_-CF_3_), −119.1 (AB system, *^2^J_FF_* = 380.5 Hz, 1F, CF_2_-CF_2_-CF_2_-CF_2_-CF_3_), −116.5 (AB system, *^2^J_FF_* = 380.5 Hz, 1F, CF_2_-CF_2_-CF_2_-CF_2_-CF_3_), −107.1 (AB system, *^2^J_FF_* = 350 Hz, 1F, CF_2_-CF_2_-CF_2_-CF_2_-CF_3_), −99.5 (AB system, *^2^J_FF_* = 350 Hz, 1F, CF_2_-CF_2_-CF_2_-CF_2_-CF_3_), −80.69 (m 3F, CF_3_). MS (*m*/*z*): 522 ([M − I]^+^, 100). HRMS calcd. for C_22_H_19_F_11_NO^+^: 522.1291, found 522.1297. Anal. Calcd. for C_22_H_19_F_11_NIO: C, 40.70; H, 2.95; N, 2.16. Found: C, 40.73; H, 2.96; N, 2.16.

*2-Perfluoropentyl-N-(4-methoxyphenyl)-6,7,8,9-tetrahydro-5H-cyclohepta[b]pyridinium (***19l***).* 6.66 g of 4-anisidine **15l** (5.41 × 10^−2^ mol) and 2.42 g (2.16 × 10^−2^ mol) of cycloheptanone **16v** were added to a solution of 9.6 g (1.8 × 10^−2^ mol) of **14** (R_F_ = C_5_F_11_) in 96 mL of dry dichloromethane. The mixture was stirred for 4 h at reflux. 10.31 g of the title product **19l** were obtained, total yield 88%. Spectral data: ^1^H-NMR (300.13 MHz, CDCl_3_) *δ* 1.7 (m, 2H), 1.9 (m, 4H), 3 (m, 2H), 3.3 (m, 2H), 3.8 (s, 3H, OCH_3_), 7 (d, *J* = 8.8 Hz, 2H), 7.6 (d, *J* = 8.4 Hz, 2H), 8.1 (d, *J* = 8.4 Hz, 1H), 8.7 (d, *J* = 8 Hz, 1H); ^13^C-NMR (75.46MHz, CDCl_3_) *δ* 24.3, 25.3, 30.7, 33.9, 35.5, 55.8, 114.6, 127.6, 128.2, 130, 138.2, 146.5, 150.2, 161.7, 168.2; ^19^F-NMR (235.3 MHz, CDCl_3_) *δ −*126 (m 2F, CF_2_), −122.3 (m 2F, CF_2_), −117.2 (m 2F, CF_2_), −101.5 (m 2F, CF_2_), −80.7 (m 3F, CF_3_). MS (*m*/*z*): 522 ([M − I]^+^, 100). HRMS calcd. for C_22_H_19_F_11_NO^+^: 522.1291, found 522.1295. Anal. Calcd. for C_22_H_19_F_11_NIO: C, 40.70; H, 2.95; N, 2.16. Found: C, 40.74; H, 2.95; N, 2.13. 

### 3.3. General Procedure for the Synthesis of 2-Trifluoromethylated and 2-Perfluoroalkylated 6-Methyl-N-(o-/p-Carboxyphenyl)Pyridinium Iodides (***17m–n’***), N-(O-/P-Carboxyphenyl)-5,6,7,8-Tetrahydroquinolinium Iodides (***18m–n***) and N-(O-Carboxyphenyl)-6,7,8,9-Tetrahydro-5H-Cyclohepta[b]Pyridinium Iodide (***19m***)

To a stirred solution of 1-acetoxy-1-iodo-perfluoroalkylethane compounds **14** or **14’** (1 equiv.) in anhydrous dichloromethane (5 mL DCM for 1 g of **14**–**14’**), was added three equiv. of the corresponding aminobenzoic acid **15m**–**n** and 1.2 equiv. of ketone **16t**–**v**. The mixture was stirred under reflux for 12 h (Table 1, Table 2 and Table 3) until complete consumption of **14**–**14’** (monitored by TLC eluent petroleum ether/ethyl acetate: 80/20 *v*/*v*, and ^19^F-NMR of aliquots). When the reaction was completed, the mixture was allowed to cool to r.t. then the brown precipitate accumulated during the reaction was separated by vacuum filtration (it was subsequently identified as anilinium salts by NMR and MS). Then a mixture of petroleum ether and ethyl ether (40/60 *v*/*v*) was added to the filtrate and the corresponding pyridinium iodides **17m–n’, 18m–n** and **19m** instantly precipitate and were isolated by vacuum filtration as amorphous solids.

*2-Perfluoropentyl-6-methyl-N-(2-carboxyphenyl)pyridinium iodide (***17m***)*. 7.34 g of 2-aminobenzoic acid **15m** (5.35 × 10^−2^ mole) and 1.58 mL or 1.24 g (2.14 × 10^−2^ mole) of acetone **16t** were added to a solution of 9.5 g (1.78 × 10^−2^ mole) of **14** (R_F_ = C_5_F_11_) in 48 mL of dry dichloromethane. The mixture was stirred for 12 h at reflux. 5.43 g of the title product **17m** were obtained, total yield 50%. ^1^H-NMR (300.13 MHz, DMSO-*d*_6_) *δ* 2.4 (s, 3H, CH_3_), 7.7–7.9 (m, 3 H), 8.2 (d, *J* = 8 Hz, 1H), 8.7 (m, 2H), 9 (t, *J* = 8 Hz, 1H), 13.9 (bs, 1H, CO_2_H); ^13^C-NMR (75.46 MHz, DMSO-*d*_6_) *δ* 22.9 (s, CH_3_), 127.9, 128.6, 129.4, 132.8, 133.6, 134.4, 137, 139.7 (t, *^2^J_CF_* = 25.1 Hz), 148.2, 163.4, 165.1 (s, CO_2_H); ^19^F-NMR (282.4 MHz, DMSO-*d*_6_) *δ −*126.7 (AB system, *^2^J_FF_* = 288.7 Hz, 1F, CF_2_-CF_2_-CF_2_-CF_2_-CF_3_), −125.7 (AB system, *^2^J_FF_* = 288.7 Hz, 1F, CF_2_-CF_2_-CF_2_-CF_2_-CF_3_), −122.8 (AB system, *^2^J_FF_* = 289 Hz, 1F, CF_2_-CF_2_-CF_2_-CF_2_-CF_3_), −121.8 (AB system, *^2^J_FF_* = 289 Hz, 1F, CF_2_-CF_2_-CF_2_-CF_2_-CF_3_), −119.2 (AB system, *^2^J_FF_* = 310.2 Hz, 1F, CF_2_-CF_2_-CF_2_-CF_2_-CF_3_), −116.3 (AB system, *^2^J_FF_* = 310.2 Hz, 1F, CF_2_-CF_2_-CF_2_-CF_2_-CF_3_), −107.5 (AB system, *^2^J_FF_* = 282.5 Hz, 1F, CF_2_-CF_2_-CF_2_-CF_2_-CF_3_), −99.1 (AB system, *^2^J_FF_* = 282.5 Hz, 1F, CF_2_-CF_2_-CF_2_-CF_2_-CF_3_), −80.69 (m 3F, CF_3_). MS (*m*/*z*): 482 ([M − I]^+^, 100). HRMS calcd. for C_18_H_11_F_11_NO_2_^+^ 482.0614, found 482.0622. Anal. Calcd. for C_18_H_11_F_11_INO_2_: C, 35.49; H, 1.82; N, 2.30. Found: C, 35.51; H, 1.82; N, 2.33.

*2-Trifluoromethyl-6-methyl-N-(2-carboxyphenyl)pyridinium iodide (***17m’***)**.*** 11.02 g of 2-aminobenzoic acid **15m** (8.04 × 10^−2^ mol) and 2.37 mL or 1.86 g (3.21 × 10^−2^ mol) of acetone **16t** were added to a solution of 8.9 g (2.68 × 10^−2^ mol) of **14’** (R_F_ = CF_3_) in 89 mL of dry dichloromethane. The mixture was stirred for 12 h at reflux. 5.7 g of the title product **17m’** were obtained, total yield 52%. Spectral data: ^1^H-NMR (300.13 MHz, DMSO-*d*_6_) *δ* 2.3 (s, 3H, C*H*_3_), 7.7–7.8 (m, 3 H), 8 (d, *J* = 8.1 Hz, 1H), 8.8 (m, 2H), 9 (t, *J* = 8 Hz, 1H), 14.2 (bs, 1H, CO_2_*H*); ^13^C-NMR (75.46 MHz, DMSO-*d*_6_) *δ* 21.9 (s, CH_3_), 118.5 (q, CF_3_, *^1^J_CF_* = 280.2 Hz), 127.5 (q, *^3^J_CF_* = 3.9 Hz), 128, 133.7, 140.2, 140.7, 146, 148.1 (q, C-CF_3_, *^2^J_CF_* = 36.6 Hz), 163, 167 (s, *C*O_2_H); ^19^F-NMR (282.4 MHz, DMSO-*d*_6_) *δ −*60.3 (s 3F, CF_3_). MS (*m*/*z*): 282 ([M − I]^+^, 100). HRMS calcd. for C_14_H_11_F_3_NO_2_^+^: 282.0742, found 282.0745. Anal. Calcd. for C_14_H_11_F_3_INO_2_: C, 41.10; H, 2.71; N, 3.42. Found: C, 41.13; H, 2.72; N, 3.40.

*2-Perfluoropentyl-6-methyl-N-(4-carboxyphenyl)pyridinium iodide (***17n***)*. 8.27 g of 4-aminobenzoic acid **15n** (6.03 × 10^−2^ mol) and 1.78 mL or 1.39 g (2.41 × 10^−2^ mol) of acetone **16t** were added to a solution of 10.7 g (2.01 × 10^−2^ mol) of **14** (R_F_ = C_5_F_11_) in 107 mL of dry dichloromethane. The mixture was stirred for 12 h at reflux. 7.35 g of the title product **17n** were obtained, total yield 60%. ^1^H-NMR (300.13 MHz, DMSO-*d*_6_) *δ* 2.5 (s, 3H, C*H*_3_), 8 (d, *J* = 8.1 Hz, 2H, Ph-*H*), 8.3 (d, *J* = 8.1 Hz, 2H, Ph-*H*), 8.8 (m, 2H, Py-*H*), 9 (t, *J* = 8 Hz, 1H, Py-*H*), 13.8 (bs, 1H, CO_2_*H*); ^13^C-NMR (75.46 MHz, DMSO-*d*_6_) *δ* 22.9 (s, *C*H_3_), 127.1, 128.7, 130.5, 133.8, 139.7, 140.7, 146, 147.6, 163.1, 165.9 (s, *C*O_2_H); ^19^F-NMR (282.4 MHz, DMSO-*d*_6_) *δ −*125.8 (s 2F, CF_2_-CF_2_-CF_2_-CF_2_-CF_3_), −122.3 (s 2F, CF_2_-CF_2_-CF_2_-CF_2_-CF_3_), −117.7 (s 2F, CF_2_-CF_2_-CF_2_-CF_2_-CF_3_), −101.8 (s, 2F, CF_2_-CF_2_-CF_2_-CF_2_-CF_3_), −80.2 (m 3F, CF_2_-CF_2_-CF_2_-CF_2_-CF_3_). MS (*m*/*z*): 482 ([M − I]^+^, 100). HRMS calcd. for C_18_H_11_F_11_NO_2_^+^: 482.0614, found 482.0620. Anal. Calcd. for C_18_H_11_F_11_INO_2_: C, 35.49; H, 1.82; N, 2.30. Found: C, 35.52; H, 1.83; N, 2.28.

*2-Trifluoromethyl-6-methyl-N-(4-carboxyphenyl)pyridinium iodide (***17n’***).*12.63 g of 4-aminobenzoic acid **15n** (9.21 × 10^−2^ mol) and 2.72 mL or 2.13 g (3.68 × 10^−2^ mol) of acetone **16t** were added to a solution of 10.2 g (3.07 × 10^−2^ mol) of **14’** (R_F_ = CF_3_) in 102 mL of dry dichloromethane. The mixture was stirred for 12 h at reflux. 7.54 g of the title product **17n’** were obtained, total yield 60%. Spectral data: ^1^H-NMR (300.13 MHz, DMSO-*d*_6_) *δ* 2.5 (s, 3H, CH_3_), 8.1 (d, *J* = 8 Hz, 2H), 8.3 (d, *J* = 8 Hz, 2H), 8.7 (m, 2H), 9.1 (t, *J* = 8 Hz, 1H), 14 (bs, 1H, CO_2_H); ^13^C-NMR (75.46 MHz, DMSO-*d*_6_) *δ* 22.7 (s, CH_3_), 118 (q, CF_3_, *^1^J_CF_* = 279 Hz), 127.1 (q, *^3^J_CF_* = 4.2 Hz), 128, 130.4, 133.7, 140, 140.7, 146, 147.6 (q, C-CF_3_, *^2^J_CF_* = 35.5 Hz), 163, 166 (s, CO_2_H); ^19^F-NMR (282.4 MHz, DMSO-*d*_6_) *δ −*59.9 (s 3F, CF_3_). MS (*m*/*z*): 282 ([M − I]^+^, 100). HRMS calcd. for C_14_H_11_F_3_NO_2_^+^: 282.0742, found 282.0747. Anal. Calcd. for C_14_H_11_F_3_INO_2_: C, 41.10; H, 2.71; N, 3.42. Found: C, 41.14; H, 2.70; N, 3.40.

*2-Perfluoropentyl-N-(2-carboxyphenyl)-5,6,7,8-tetrahydroquinolinium iodide (***18m***)*. 7.88 g of 2-aminobenzoic acid **15m** (5.75 × 10^−2^ mol) and 2.25 g (2.3 × 10^−2^ mole) of cyclohexanone **16u** were added to a solution of 10.2 g (1.91 × 10^−2^ mol) of **14** (R_F_ = C_5_F_11_) in 51 mL of dry dichloromethane. The mixture was stirred for 12 h at reflux. 8.96 g of the title product **18m** were obtained, total yield 72%. ^1^H-NMR (300.13 MHz, CDCl_3_) *δ* 1.8 (s, 2H, CH_2_, cyclohexyl group), 2 (s, 2H, CH_2_, cyclohexyl group), 2.4 (m, 1H, CH_2_, cyclohexyl group), 2.7 (m, 1H, CH_2_, cyclohexyl group), 3.1 (m, 1H, CH_2_, cyclohexyl group), 3.3 (m, 1H, CH_2_, cyclohexyl group), 6.9 (bs, 1H, CO_2_*H*), 7.8 (m, 3H, Ph-*H*), 8.2 (d, *J* = 7.4 Hz, 1H, Ph-*H*), 8.4 (d, *J* = 8.1 Hz, 1H, Py-*H*), 8.9 (d, *J* = 8.2 Hz, 1H, Py-*H*); ^1^H-NMR (400.13 MHz, DMSO-*d*_6_) *δ* 1.6–1.8 (m, 4H, CH_2_, cyclohexyl group), 2.3 (m, 1H, CH_2_, cyclohexyl group), 2.6 (m, 1H, CH_2_, cyclohexyl group), 3.2 (m, 2H, CH_2_, cyclohexyl group), 7.8–8 (m, 3H, Ph-*H*), 8.3 (d, *J* = 8 Hz, 1H, Ph-*H*), 8.6 (d, *J* = 8 Hz, 1H, Py-*H*), 8.9 (d, *J* = 8 Hz, 1H, Py-*H*); ^13^C-NMR (75.46 MHz, CDCl_3_) *δ* 19.9, 21.4, 29.5, 30.5, 126.5, 126.7, 130.6, 132.2, 132.7, 133.2, 136.4, 139.3 (t, *^2^J_CF_* = 25 Hz), 144.4, 147.5, 161.6, 165.8 (s, *C*O_2_H); ^13^C-NMR (100.6 MHz, DMSO-*d*_6_) *δ* 20, 21.4, 29.1, 30.3, 127.2 (t, *^3^J_CF_* = 4 Hz, =*C*-H, Py), 128.5, 132.6, 133, 134.6, 136.6, 137.2, 137.4 (t, *^2^J_CF_* = 22.13 Hz, =*C*-CF_2_, Py), 144.7, 148, 162.2, 164.9 (s, *C*O_2_H); ^19^F-NMR (282.4 MHz, DMSO-*d*_6_) *δ −*126.5 (AB system, *^2^J_FF_* = 282 Hz, 1F, CF_2_-CF_2_-CF_2_-CF_2_-CF_3_), −125.3 (AB system, *^2^J_FF_* = 282 Hz, 1F, CF_2_-CF_2_-CF_2_-CF_2_-CF_3_), −123 (AB system, *^2^J_FF_* = 367 Hz, 1F, CF_2_-CF_2_-CF_2_-CF_2_-CF_3_), −121.9 (AB system, *^2^J_FF_* = 367 Hz, 1F, CF_2_-CF_2_-CF_2_-CF_2_-CF_3_), −118.9 (AB system, *^2^J_FF_* = 338 Hz, 1F, CF_2_-CF_2_-CF_2_-CF_2_-CF_3_), −117 (AB system, *^2^J_FF_* = 338 Hz, 1F, CF_2_-CF_2_-CF_2_-CF_2_-CF_3_), −107 (AB system, *^2^J_FF_* = 310 Hz, 1F, CF_2_-CF_2_-CF_2_-CF_2_-CF_3_), −98.5 (AB system, *^2^J_FF_* = 310 Hz, 1F, CF_2_-CF_2_-CF_2_-CF_2_-CF_3_), −80.31 (m 3F, CF_3_). MS (*m*/*z*): 522 ([M − I]^+^, 98). HRMS calcd. for C_21_H_15_F_11_NO_2_^+^ 522.0927, found 522.0933. Anal. Calcd. for C_21_H_15_F_11_NIO_2_: C, 38.85; H, 2.33; N, 2.16. Found: C, 38.88; H, 2.34; N, 2.15.

*2-Perfluoropentyl-N-(4-carboxyphenyl)-5,6,7,8-tetrahydroquinolinium iodide (***18n***).* 8.27 g of 4-aminobenzoic acid **15n** (6.03 × 10^−2^ mol) and 2.36 g (2.41 × 10^−2^ mol) of cyclohexanone **16u** were added to a solution of 10.7 g (2.01 × 10^−2^ mol) of **14** (R_F_ = C_5_F_11_) in 53.5 mL of dry dichloromethane. The mixture was stirred for 12 h at reflux. 9.14 g of the title product **18n** were obtained, total yield 70%. Spectral data: ^1^H-NMR (300.13 MHz, CDCl_3_) *δ* 2 (m, 4H), 2.6 (m, 2H), 3.4 (m, 2H), 7.9 (d, *J* = 8 Hz, 2H), 8.2 (d, *J* = 8 Hz, 2H), 8.8 (d, *J* = 8.1 Hz, 1H), 9 (d, *J* = 8.1 Hz, 1H) 12.9 (bs, 1H, CO_2_*H*); ^13^C-NMR (75.46MHz, CDCl_3_) *δ* 20.8, 21, 29.2, 31.5, 125.5, 127, 128.4, 129.5, 133.7, 139.7 (t, *^2^J_CF_* = 24.3 Hz), 146, 147.2, 163.1, 166 (s, CO_2_H); ^19^F-NMR (235.3 MHz, CDCl_3_) *δ −*125.9 (m 2F, CF_2_), −122.5 (m 2F, CF_2_), −117.7 (m 2F, CF_2_), −101.5 (m 2F, CF_2_), −80.4 (m 3F, CF_3_). MS (*m*/*z*): 522 ([M − I]^+^, 90). HRMS calcd. for C_21_H_15_F_11_NO_2_^+^: 522.0927, found 522.0931. Anal. Calcd. for C_21_H_15_F_11_NIO_2_: C, 38.85; H, 2.33; N, 2.16. Found: C, 38.89; H, 2.34; N, 2.15.

*2-Perfluoropentyl-N-(2-carboxyphenyl)-6,7,8,9-tetrahydro-5H-cyclohepta[b]pyridinium (***19m***).* 8.35 g of 2-aminobenzoic acid **15m** (6.09 × 10^−2^ mol) and 2.73 g (2.43 × 10^−2^ mole) of cycloheptanone **16v** were added to a solution of 10.8 g (2.03 × 10^−2^ mole) of **14** (R_F_ = C_5_F_11_) in 54 mL of dry dichloromethane. The mixture was stirred for 6 h at reflux. 9.15 g of the title product **19m** were obtained, total yield 68%. ^1^H-NMR (300.13 MHz, CDCl_3_) *δ* 1.4–2 (m, 6H, CH_2_, cycloheptyl group), 2.7 (m, 1H, CH_2_, cycloheptyl group), 3 (m, 1H, CH_2_, cycloheptyl group), 3.3–3.4 (m, 2H, CH_2_, cycloheptyl group), 7.7 (bs, 1H, CO_2_*H*), 7.77 (m, 3H, Ar-*H*), 8.1 (d, *J* = 8 Hz, 1H, Ar-*H*), 8.3 (d, *J* = 8 Hz, 1H, Py-*H*), 8.9 (d, *J* = 8 Hz, 1H, Py-*H*); ^1^H-NMR (300.13 MHz, DMSO-*d*_6_) *δ* 1.4 (m, 1H, CH_2_, cycloheptyl group), 1.6–1.9 (m, 5H, CH_2_, cycloheptyl group), 2.8 (m, 2H, CH_2_, cycloheptyl group), 3.3 (m, 2H, CH_2_, cycloheptyl group), 7.9 (m, 2H, Ar-*H*), 8 (m, 1H, Ar-*H*), 8.4 (d, *J* = 6 Hz, 1H, Ar-*H*), 8.6 (d, *J* = 6.15 Hz, 1H, Py-*H*), 8.9 (d, *J* = 6.18 Hz, 1H, Py-*H*); ^13^C-NMR (75.46MHz, DMSO-*d*_6_) *δ* 24, 25.5, 30.4, 33.4, 35.3, 127.2, 127.5, 129.7, 132.2, 132.6, 132.9, 137.1, 138.2 (t, *^2^J_CF_* = 20.1 Hz), 147.1, 149.2, 165.1, 166.6; ^19^F-NMR (282.4 MHz, DMSO-*d*_6_) *δ −*126.6 (AB system, *^2^J_FF_* = 293.4 Hz, 1F, CF_2_-CF_2_-CF_2_-CF_2_-CF_3_), −125.5 (AB system, *^2^J_FF_* = 293.4 Hz, 1F, CF_2_-CF_2_-CF_2_-CF_2_-CF_3_), −123.4 (AB system, *^2^J_FF_* = 297.1 Hz, 1F, CF_2_-CF_2_-CF_2_-CF_2_-CF_3_), −122.3 (AB system, *^2^J_FF_* = 297.1 Hz, 1F, CF_2_-CF_2_-CF_2_-CF_2_-CF_3_), -119 (AB system, *^2^J_FF_* = 300.96 Hz, 1F, CF_2_-CF_2_-CF_2_-CF_2_-CF_3_), −116.5 (AB system, *^2^J_FF_* = 300.96 Hz, 1F, CF_2_-CF_2_-CF_2_-CF_2_-CF_3_), −108.2 (AB system, *^2^J_FF_* = 285.9 Hz, 1F, CF_2_-CF_2_-CF_2_-CF_2_-CF_3_), −97.9 (AB system, *^2^J_FF_* = 285.9 Hz, 1F, CF_2_-CF_2_-CF_2_-CF_2_-CF_3_), −80.86 (m 3F, CF_3_). MS (*m*/*z*): 536 ([M − I]^+^, 95). HRMS calcd. for C_22_H_17_F_11_NO_2_^+^ 536.1084, found 536.1089. Anal. Calcd. for C_22_H_17_F_11_NIO_2_: C, 39.84; H, 2.58; N, 2.11. Found: C, 39.86; H, 2.59; N, 2.10. 

### 3.4. General Procedure for the Synthesis 2-Trifluoromethyl or 2-Perfluoroalkyl-7-Methoxyquinolines ***20o–o’***

To a stirred solution of 1-acetoxy-1-iodo-perfluoroalkylethane compounds **14** or **14’** (1 equiv.) in anhydrous dichloromethane (10 mL DCM for 1g of **14**–**14’**), was added 3 equiv. of *meta*-anisidine **15o**. The mixture was stirred under reflux for the desired time (12 h, Table 4) until complete consumption of **14**–**14’** (monitored by TLC eluent petroleum ether/ethyl acetate: 80/20 *v*/*v*, and ^19^F-NMR of aliquots). When the reaction was completed, the mixture was concentrated under reduced pressure and then stirred with diethyl ether. An excess of petroleum ether was added; the precipitate that had formed was eliminated by vacuum filtration and washed three times with petroleum ether. The filtrate was concentrated in vacuo to give a brown oil. Chromatography over silica gel column (eluent, petroleum ether/ethyl acetate 98/2 *v*/*v*) left a yellow oil which was crystallized from methanol/water to give pure samples of the corresponding quinolines **20o**–**o’**. 

### 3.5. General Procedure for the Synthesis 2-Trifluoromethyl or 2-Perfluoroalkyl-7-Methoxyquinolines ***20o***–***o’*** in the Presence of Ketones ***16t***–***v***

To a stirred solution of 1-acetoxy-1-iodo-perfluoroalkylethane compounds **14** or **14’** (1 equiv.) in anhydrous dichloromethane (10 mL DCM for 1 g of **14**–**14’**), was added three equiv. of *meta*-anisidine **15o** and 1.2 equiv. of ketone **16t**–**v**. The mixture was stirred under reflux for desired time (12 h, Table 4) until complete consumption of **14**–**14’** (monitored by TLC eluent petroleum ether/ethyl acetate: 80/20 *v*/*v*, and ^19^F-NMR of aliquots). When the reaction was completed, the mixture was concentrated under reduced pressure and then stirred with a mixture of petroleum ether/ethyl acetate. Chromatography over silica gel column (eluent, petroleum ether/ethyl acetate 98/2 *v*/*v*) left a yellow oil which was crystallized from methanol/water to give pure samples of the corresponding quinolines **20o**–**o’**. We were able to isolate unreacted ketones **16t**–**v** from the corresponding reactions (1.2 equivalent) which were identified and characterized by NMR and mass spectroscopy

*2-perfluoropentyl-6-methoxyquinoline (***20o***).* 6.25 g of *meta*-anisidine **15o** (5.07 × 10^−2^ mol) and 2.03 × 10^−2^ mole of the corresponding ketone (1.17 g of acetone **16t** or 1.99 g of cyclohexanone **16u** or 2.27 g of cylcoheptanone **16v**) were added to a solution of 9 g (1.69 × 10^−2^ mole) of **14** (R_F_ = C_5_F_11_) in 90 mL of dry dichloromethane. The mixture was stirred for 12 h at reflux. In the presence of acetone: 6.1 g of **20o** (85% yield) and 0.5 g of **16t**. Or in the presence of cyclohexanone: 5.9 g of **20o** (82% yield) and 1.9 g of **16u**. Or in the case of cycloheptanone: 5.9 g of **20o** (82% yield) and 2.25 g of **16u** were obtained respectively. 

Or 6.8 g of *meta*-anisidine **15o** (5.52 × 10^−2^ mol) were added to a solution of 9.8 g (1.84 ×10^−2^ mol) of **14** (R_F_ = C_5_F_11_) in 98 mL of dry dichloromethane. The mixture was stirred for 12 h at reflux. 6.6 g (84% yield) of quinoline **20o** were obtained respectively. ^1^H-NMR (300.13 MHz, CDCl_3_) *δ* 3.8 (s, 3H, OC*H*_3_), 7.5 (d, *J* = 8.5 Hz, 1H), 7.8 (d, *J* = 8.6 Hz, 1H), 8 (m, 2H), 8.6 (d, *J* = 8.6 Hz, 1H); ^13^C-NMR (75.46 MHz, CDCl_3_) *δ* 55.6 (s, O*C*H_3_), 117.6, 126.5, 130.5, 130.7, 135.2, 138.7, 139.8, 146.3, 147.8 (t, *^2^J_CF_* = 26 Hz, *C*-CF_2_); ^19^F-NMR (282.4 MHz, CDCl_3_) *δ −*126.5 (m 2F, CF_2_-CF_2_-CF_2_-CF_2_-CF_3_), −122.5 (m 2F, CF_2_-CF_2_-CF_2_-CF_2_-CF_3_), −122 (m 2F, CF_2_-CF_2_-CF_2_-CF_2_-CF_3_), −114.5 (m, 2F, CF_2_-CF_2_-CF_2_-CF_2_-CF_3_), −81.2 (m 3F, CF_2_-CF_2_-CF_2_-CF_2_-CF_3_). MS (*m*/*z*): 428 [M + H]^+^. HRMS *m*/*z* [M + H]^+^ calcd. for C_15_H_9_F_11_NO^+^: 428.0508, found: 428.0510. Anal calcd. for C_15_H_8_F_11_NO: C, 42.17; H, 1.89; N, 3.28, found: C, 42.19; H, 1.88; N, 3.26.

*2-Trifluoromethyl-6-methoxyquinoline (***20o’***).* 10.9 g of 3-anisidine **15o** (8.85 × 10^−2^ mol) and 2.6 mL or 2 g (3.54 × 10^−2^ mol) of acetone **16t** were added to a solution of 9.8 g (2.95 × 10^−2^ mol) of **14’** (R’_F_ = C_2_F_5_) in 98 mL of dry dichloromethane. The mixture was stirred for 12 h at reflux. 5.9 g of the quinoline **20o’** were obtained, total yield 88%. Spectral data: ^1^H-NMR (300.13 MHz, CDCl_3_) *δ* 3.8 (s, 3H, OCH_3_), 7.6 (d, *J* = 8.4 Hz, 1H), 7.8 (d, *J* = 8.5 Hz, 1H), 7.9–8 (m, 2H), 8.5 (d, *J* = 8.5 Hz, 1H); ^13^C-NMR (75.46 MHz, CDCl_3_) *δ* 55.5 (s, OCH_3_), 116.5 (q, *^3^J_CF_* = 2.1 Hz, CH-C-CF_3_), 122.8 (q, *^1^J_CF_* = 275.6 Hz, CF_3_), 128, 128.5, 129.3, 132.1, 136, 139.1, 142.6, 148.5 (q, *^2^J_CF_* = 34 Hz, C-CF_3_); ^19^F-NMR (282.4 MHz, CDCl_3_) *δ −* 67.8 (m 3F, CF_3_). MS (*m*/*z*): 228 [M + H]^+^. HRMS *m*/*z* [M + H]^+^ calcd. for C_11_H_9_F_3_NO^+^: 228.0636, found: 228.0643. Anal calcd. for C_11_H_8_F_3_NO: C, 58.15; H, 3.55; N, 6.17, found: C, 58.17; H, 3.56; N, 6.15.

### 3.6. General Procedure for the Preparation and Isolation of 2-Perfluoroalkyl- and 2-Trifluoromethyl-1-(R-phenyl)amino-3-(R-phenyl)iminopropene Intermediates ***21a***–***l*** (all examples except R = CO_2_H or R = m-OMe)

A mixture of one equivalent of *gem*-iodoacetate compounds **14** or **14’** or **14”** and two equivalents of the corresponding anilines **15a–l** in dichloromethane (10 mL DCM for 1 g of **14**–**14”**) was stirred at room temperature until disappearance of ^19^F-NMR signals corresponding to the starting products **14**–**14”** (2–6 h). At the end of the reaction, a solution of 10% sodium thiosulfate was added to the reaction mixture and the product was extracted three times with ether. The combined extracts were washed several times with aqueous 0.5 M hydrochloric solution, dried over anhydrous sodium sulfate and concentrated under reduced pressure to give a bright yellow oil. Chromatography over silica gel column (eluent: petroleum ether/ethyl acetate 98/2 *v*/*v*) yielded the pure compounds **21a**–**l** as yellow liquids.

*3-Perfluoropenthyl-1-phenylamino-3-phenyliminopropene (***21a***).* 3.49 g of aniline **15a** (3.75 × 10^−2^ mol) were added to a solution of 10 g (1.87 × 10^−2^ mol) of **14** (R_F_ = C_5_F_11_) in 100 mL of dry dichloromethane. The mixture was stirred for 4 h at room temperature. 8.28 g of the title product **21a** were obtained, total yield 90%. ^1^H-NMR (300.13 MHz, DMSO-*d*_6_, *EEE*-**21a**) *δ* 5.5 (d, *^3^J_HH_* = 13 Hz, 1H, C*H*=CH-NH), 6.8 (t (AB system), *J* = 8.7 Hz, 4H*_ortho_*, Ph-*H*), 6.9 (t, *J* = 7.4 Hz, 1H*_para_*, Ph-*H*), 7.1 (t, *J* = 7.4 Hz, 1H*_para_*, Ph-*H*), 7.2 (t, *J* = 8.1 Hz, 2H*_meta_*, Ph-*H*), 7.4 (t, *J* = 7.8 Hz, 2H*_meta_*, Ph-*H*), 7.45 (m, 1H, CH=C*H*-NH), 9.9 (d, *^3^J_HNH_* = 12.8 Hz, CH=CH-N*H*); ^1^H-NMR (300.13 MHz, DMSO-*d*_6_/D_2_O, *EEE*-**21a**) *δ* 5.4 (d, *^3^J_HH_* = 13 Hz, 1H, C*H*=CH-NH), 6.7 (d, *J* = 8.5 Hz, 4H*_ortho_*, Ph-*H*), 6.9 (t, *J* = 7.5 Hz, 1H*_para_*, Ph-*H*), 7.1 (t, *J* = 7.4 Hz, 1H*_para_*, Ph-*H*), 7.2 (t, *J* = 8 Hz, 2H*_meta_*, Ph-*H*), 7.4 (t, *J* = 8 Hz, 2H*_meta_*, Ph-*H*), 7.45 (m, 1H, CH=C*H*-NH); ^13^C-NMR (75.46 MHz, DMSO-*d*_6_, *EEE*-**21a**) *δ * 91.5, 114.6, 118.5, 122.1, 123.5, 129.1, 129.5, 130, 140.5, 141.5 (t, *^3^J_CF_* = 5.4 Hz, CF_2_-(C=N)-*C*H=CH-NH), 149.9, 153.6 (t, *^2^J_C1F_* = 22.5 Hz, CF_2_-(*C*=N)-CH=CH-NH); ^19^F-NMR (282.4 MHz, DMSO-*d*_6_, *EEE*-**21a**) *δ −*80.2 (t, *J* = 8.4 Hz, 3F, CF_2_-CF_2_-CF_2_-CF_2_-CF_3_), −109.5 (t, *J* = 11 Hz, 2F, CF_2_-CF_2_-CF_2_-CF_2_-CF_3_), −120.4 (m, 2F, CF_2_-CF_2_-CF_2_-CF_2_-CF_3_), −121 (q, *J* = 5.6 Hz, 2F, CF_2_-CF_2_-CF_2_-CF_2_-CF_3_), −125.8 (t, *J* = 11.2 Hz, 2F, CF_2_-CF_2_-CF_2_-CF_2_-CF_3_). MS (*m*/*z*): 491 [M + H]^+^. HRMS calcd. for C_20_H_14_F_11_N_2_^+^: 491.0981, found: 491.0985; Anal calcd. for C_20_H_13_F_11_N_2_: C, 48.99; H, 2.67; N, 5.71, found C, 48.97; H, 2.66; N, 5.74.

*3-Trifluoromethyl-1-phenylamino-3-phenyliminopropene (***21a’***).* 3.64 g of aniline **15a** (3.91 × 10^−2^ mol) were added to a solution of 6.5 g (1.95 × 10^−2^ mol) of **14’** (R_F_ = CF_3_) in 65 mL of dry dichloromethane. The mixture was stirred for 3 h at room temperature. 4.65 g of the title product **21a’** were obtained, total yield 82%. ^1^H-NMR (300.13 MHz, DMSO-*d*_6_, *EEE*-**21a**’) *δ* 5.5 (d, *J* = 13.7 Hz, 1H, C*H*=CH-NH), 6.8 (d, *J* = 7.5 Hz, 2H), 7 (m, 3H), 7.15 (t, *J* = 7.4 Hz, 1H), 7.3 (t, *J* = 7.8 Hz, 2H), 7.4 (t, *J* = 7.7 Hz, 2H), 7.6 (t, *J* = 13 Hz, 1H, CH=C*H*-NH), 9.9 (d, *J* = 12.3 Hz, 1H, CH=CH-N*H*); ^13^C-NMR (75.46 MHz, DMSO-*d*_6_, *EEE*-**21a’**) *δ* 90.8, 115.1, 119.4, 120.5 (q, CF_3_, *^1^J_CF_* = 279.2 Hz), 122.2, 123.5, 129.1, 129.5, 140.64, 140.8 (q, *^3^J_CF_* = 3 Hz, CF_3_-(C=N)-*C*H=CH-NH), 149.5, 153.3 (q, *^2^J_CF_* = 30.5 Hz, CF_3_-(*C*=N)-CH=CH-NH); ^19^F-NMR (282.4 MHz, DMSO-*d*_6_, *EEE*-**21a’**) *δ −*65.7 (s, 3F). MS (*m*/*z*): 291 [M + H]^+^. HRMS calcd. for C_16_H_14_F_3_N_2_^+^: 291.1109, found 291.1110. Anal. Calcd. for C_16_H_13_F_3_N_2_: C, 66.20; H, 4.51; N, 9.65. Found: C, 66.19; H, 4.48; N, 9.55.

*3-perfluoropropyl-1-phenylamino-3-phenyliminopropene (***21a”***).* 2.36 g of aniline **15a** (2.54 × 10^−2^ mol) were added to a solution of 5.5 g (1.27 × 10^−2^ mol) of **14”** (R_F_ = C_3_F_7_) in 55 mL of dry dichloromethane. The mixture was stirred for 3 h at room temperature. 4.22 g of the title product **21a”** were obtained, total yield 85%. ^1^H-NMR (300.13 MHz, DMSO-*d*_6_, *EEE*-**21a”**) *δ* 5.5 (d, *J* = 13.5 Hz, 1H, C*H*=CH-NH), 6.8 (d, *J* = 7.4 Hz, 2H), 7 (m, 3H), 7.2 (t, *J* = 7.4 Hz, 1H), 7.3 (t, *J* = 7.7 Hz, 2H), 7.4 (t, *J* = 7.7 Hz, 2H), 7.7 (t, *J* = 13.1 Hz, 1H, CH=C*H*-NH), 9.9 (d, *J* = 12.1 Hz, 1H, CH=CH-N*H*); ^13^C-NMR (75.46 MHz, DMSO-*d*_6_, *EEE*-**21a’’**) *δ * 90.5, 115, 118.5, 122, 123.5, 129.3, 129.7, 130, 140.5, 141 (t, *^3^J_CF_* = 5 Hz, CF_2_-(C=N)-*C*H=CH-NH), 150, 153.6 (t, *^2^J_CF_* = 21.5 Hz, CF_2_-(*C*=N)-CH=CH-NH); ^19^F-NMR (282.4 MHz, DMSO-*d*_6_, *EEE*-**21a”**) *δ −*80.5 (m, 3F), −109.1 (t, *J* = 11 Hz, 2F), −126 (m, 2F). MS (*m*/*z*): 391 [M + H]^+^. HRMS calcd. for C_18_H_14_F_7_N_2_^+^: 391.1045, found: 391.1055; Anal calcd. for C_18_H_13_F_7_N_2_: C, 55.39; H, 3.36; N, 7.18, found C, 55.41; H, 3.35; N, 7.15. 

*3-perfluoropentyl-1-(2-methylphenylamino)-3-(2-methylphenylimino)-propene* (**21b***).* 3.26 g of 2-methylaniline **15b** (3.04 × 10^−2^ mol) were added to a solution of 8.1 g (1.52 × 10^−2^ mol) of **14** (R_F_ = C_5_F_11_) in 81 mL of dry dichloromethane. The mixture was stirred for 6 h at room temperature. 5.52 g of the title product **21b** were obtained, total yield 70%. ^1^H-NMR (300.13 MHz, DMSO-*d*_6_, *EEE*-**21b**) *δ* 2.1 (s, 3H), 2.3 (s, 3H), 5.6 (d, *J* = 13.1 Hz, C*H*=CH-NH), 6.5–7.3 (m, 8H, H_Ar_), 7.6 (m, 1H, CH=C*H*-NH), 9.3 (bs, 1H, CH=CH-N*H*); ^13^C-NMR (75.46 MHz, DMSO-*d*_6_, *EEE*-**21b**) *δ* 18.8, 18.9, 89, 113.1, 117.7, 121.5, 123.5, 129, 129.3, 130, 140.5, 141.5 (t, *^3^J_CF_* = 4.8 Hz, CF_2_-(C=N)-*C*H=CH-NH), 150.1, 153.5 (t, *^2^J_C1F_* = 20.5 Hz, CF_2_-(*C*=N)-CH=CH-NH); ^19^F-NMR (282.4 MHz, DMSO-*d*_6_, *EEE*-**21b**) *δ −*80.5 (m, 3F), −109 (m, 2F), −120.5 (m, 2F), −121 (m, 2F), −125.5 (m, 2F). MS (*m*/*z*): 519 [M + H]^+^. HRMS calcd. for C_22_H_18_F_11_N_2_^+^: 519.1294, found: 519.1298; Anal calcd. for C_22_H_17_F_11_N_2_: C, 50.97; H, 3.31; N, 5.40, found C, 50.99; H, 3.30; N, 5.37.

*3-perfluoropentyl-1-(3-methylphenylamino)-3-(3-methylphenylimino)-propene (***21c***)*. 3.22 g of 3-methylaniline **15c** (3.10^−2^ mol) were added to a solution of 8 g (1.5 × 10^−2^ mol) of **14** (R_F_ = C_5_F_11_) in 80 mL of dry dichloromethane. The mixture was stirred for 4 h at room temperature. 6.23 g of the title product **21c** were obtained, total yield 80%. ^1^H-NMR (300.13 MHz, DMSO-*d*_6_, *EEE*-**21c**) *δ* 2.2 (s, 3H), 2.3 (s, 3H), 5.4 (d, *J* = 13.6 Hz, 1H, C*H*=CH-NH), 6.6 (m, 2H), 6.7 (m, 3H), 6.9 (d, *J* = 7.6 Hz, 1H), 7.1 (m, 1H), 7.2 (t, *J* = 7.6 Hz, 1H), 7.5 (t, *J* = 12.5 Hz, 1H, CH=C*H*-NH), 9.7 (d, *J* = 12.5 Hz, 1H, CH=CH-N*H*); ^13^C-NMR (75.46 MHz, DMSO-*d*_6_, *EEE*-**21c**) *δ* 20.5, 20.9, 89, 113.5, 117.5, 121.5, 123.5, 129, 129.5, 130.2, 140, 141.1 (t, *^3^J_CF_* = 5.3 Hz, CF_2_-(C=N)-*C*H=CH-NH), 150, 153 (t, *^2^J_C1F_* = 21.5 Hz, CF_2_-(*C*=N)-CH=CH-NH); ^19^F-NMR (282.4 MHz, DMSO-*d*_6_, *EEE*-**21c**) *δ −*80.5 (m, 3F), −109.6 (m, 2F), −120.2 (m, 2F), −121.5 (m, 2F), −125.5 (m, 2F). MS (*m*/*z*): 519 [M + H]^+^. HRMS calcd. for C_22_H_18_F_11_N_2_^+^: 519.1294, found: 519.1295; Anal calcd. for C_22_H_17_F_11_N_2_: C, 50.97; H, 3.31; N, 5.40, found C, 50.99; H, 3.28; N, 5.38

*3-perfluoropentyl-1-(4-methylphenylamino)-3-(4-methylphenylimino)-propene (***21d***)*. 3.62 g of 4-methylaniline **15d** (3.38 × 10^−2^ mol) were added to a solution of 9 g (1.69 × 10^−2^ mol) of **14** (R_F_ = C_5_F_11_) in 90 mL of dry dichloromethane. The mixture was stirred for 4 h at room temperature. 7.71 g of the title product **21d** were obtained, total yield 88%. ^1^H-NMR (300.13 MHz, DMSO-*d*_6_, *EEE*-**21d**) *δ* 2.2 (s, 3H), 2.3 (s, 3H), 5.4 (d, *J* = 13 Hz, 1H, C*H*=CH-NH), 6.7 (d, *J* = 8 Hz, 2H), 6.8 (d, *J* = 8.2 Hz, 2H), 7.1 (d, *J* = 8.2 Hz, 2H), 7.2 (d, *J* = 8 Hz, 2H), 7.5 (t, *J* = 12.5 Hz, 1H, CH=C*H*-NH), 9.7 (d, *J* = 12.4 Hz, 1H, CH=CH-N*H*); ^13^C-NMR (75.46 MHz, DMSO-*d*_6_, *EEE*-**21d**) *δ* 20.1, 20.4, 90.2, 115.5, 117.5, 122, 124.5, 129.2, 129.5, 140, 141.9 (t, *^3^J_CF_* = 3.5 Hz, CF_2_-(C=N)-*C*H=CH-NH), 150.5, 152.9 (t, *^2^J_C1F_* = 31.1 Hz, CF_2_-(*C*=N)-CH=CH-NH); ^19^F-NMR (282.4 MHz, DMSO-*d*_6_, *EEE*-**21d**) *δ −*80.3 (m, 3F), −109.5 (m, 2F), −120.1 (m, 2F), −121.5 (m, 2F), −125 (m, 2F). MS (*m*/*z*): 519 [M + H]^+^. HRMS calcd. for C_22_H_18_F_11_N_2_^+^: 519.1294, found: 519.1290; Anal calcd. for C_22_H_17_F_11_N_2_: C, 50.97; H, 3.31; N, 5.40, found C, 50.97; H, 3.29; N, 5.42

*3-Trifluoromethyl-1-(4-methylphenylamino)-3-(4-methylphenylimino)-propene (***21d’***).* 5.48 g of 4-methylaniline **15d** (5.12 × 10^−2^ mol) were added to a solution of 8.5 g (2.56 × 10^−2^ mol) of **14’** (R_F_ = CF_3_) in 85 mL of dry dichloromethane. The mixture was stirred for 4 h at room temperature. 7 g of the title product **21d’** were obtained, total yield 86%. ^1^H-NMR (300.13 MHz, DMSO-*d*_6_, *EEE*-**21d’**) *δ* 2.2 (s, 3H), 2.3 (s, 3H), 5.4 (d, *J* = 13.8 Hz, 1H, C*H*=CH-NH), 6.7 (d, *J* = 8 Hz, 2H), 6.8 (d, *J* = 8.2 Hz, 2H), 7.1 (d, *J* = 8.2 Hz, 2H), 7.2 (d, *J* = 8 Hz, 2H), 7.5 (t, *J* = 12.9 Hz, 1H, CH=C*H*-NH), 9.7 (d, *J* = 12.4 Hz, 1H, CH=CH-N*H*); ^13^C-NMR (75.46 MHz, DMSO-*d*_6_, *EEE*-**21d’**) *δ* 20.1, 20.3, 90.4, 115, 119.1, 120.6 (q, *^1^J_CF_* = 279.5 Hz, CF_3_), 129.1, 129.7, 129.9, 130.8, 131.1, 132.5, 138.2, 140.8 (q, *^3^J_CF_* = 3.1 Hz, CF_3_-(C=N)-*C*H=CH-NH), 147, 153.4 (q, *^2^J_CF_* = 30.8 Hz, CF_3_-(*C*=N)-CH=CH-NH); ^19^F-NMR (282.4 MHz, DMSO-*d*_6_, *EEE*-**21d’**) *δ −*65.7 (s, 3F). MS (*m*/*z*): 319 [M + H]^+^. HRMS calcd. for C_18_H_18_F_3_N_2_^+^: 319.1422, found: 319.1425; Anal calcd. for C_18_H_17_F_3_N_2_: C, 67.91; H, 5.38; N, 8.80, found C, 67.95; H, 5.37; N, 8.82.

*3-perfluoropentyl-1-(2-ethylphenylamino)-3-(2-ethylphenylimino)-propene (***21e***)*. 3.59 g of 2-ethylaniline **15e** (2.96 × 10^−2^ mol) were added to a solution of 7.9 g (1.48 × 10^−2^ mol) of **14** (R_F_ = C_5_F_11_) in 79 mL of dry dichloromethane. The mixture was stirred for 6 h at room temperature. 5.43 g of the title product **21e** were obtained, total yield 67%. ^1^H-NMR (300.13 MHz, DMSO-*d*_6_, *EEE*-**21e**) *δ* 1.2 (t, *J* = 8 Hz, 3H, CH_2_-C*H*_3_), 1.3 (t, *J* = 8 Hz, 3H, CH_2_-C*H*_3_), 2.5 (q, *J* = 8 Hz, 2H, C*H*_2_-CH_3_), 2.7(q, *J* = 8 Hz, 2H, C*H*_2_-CH_3_), 5.6 (d, *J* = 12.8 Hz, C*H*=CH-NH), 6.5–7.3 (m, 8H, H_Ar_), 7.5 (m, 1H, CH=C*H*-NH), 9.1 (bs, 1H, CH=CH-N*H*); ^13^C-NMR (75.46 MHz, DMSO-*d*_6_, *EEE*-**21e**) *δ* 13.2 (s, CH_2_-*C*H_3_), 13.3 (s, CH_2_-*C*H_3_), 24.1 (s, *C*H_2_-CH_3_), 24.3 (s, *C*H_2_-CH_3_), 89, 112.9, 117.5, 121.2, 129.3, 129.4, 130.5, 140.5, 141 (t, *^3^J_CF_* = 4.9 Hz, CF_2_-(C=N)-*C*H=CH-NH), 149.9, 153.1 (t, *^2^J_C1F_* = 21.5 Hz, CF_2_-(*C*=N)-CH=CH-NH); ^19^F-NMR (282.4 MHz, DMSO-*d*_6_, *EEE*-**21e**) *δ −*80.6 (m, 3F), −109.2 (m, 2F), −120.1 (m, 2F), −121.1 (m, 2F), −125 (m, 2F). MS (*m*/*z*): 547 [M + H]^+^. HRMS calcd. for C_24_H_22_F_11_N_2_^+^: 547.1607, found: 547.1610; Anal calcd. for C_24_H_21_F_11_N_2_: C, 52.75; H, 3.87; N, 5.13, found C, 52.76; H, 3.88; N, 5.10.

*3-perfluoropentyl-1-(3-ethylphenylamino)-3-(3-ethylphenylimino)-propene (***21f***)*. 3.87 g of 3-ethylaniline **15f** (3.19 × 10^−2^ mol) were added to a solution of 8.5 g (1.59 × 10^−2^ mol) of **14** (R_F_ = C_5_F_11_) in 85 mL of dry dichloromethane. The mixture was stirred for 4 h at room temperature. 6.71 g of the title product **21f** were obtained, total yield 77%. ^1^H-NMR (300.13 MHz, DMSO-*d*_6_, *EEE*-**21f**) *δ* 1.1 (t, *J* = 8 Hz, 3H, CH_2_-C*H*_3_), 1.3 (t, *J* = 8 Hz, 3H, CH_2_-C*H*_3_), 2.6 (q, *J* = 8 Hz, 2H, C*H*_2_-CH_3_), 2.7 (q, *J* = 8 Hz, 2H, C*H*_2_-CH_3_), 5.4 (d, *J* = 13.1 Hz, 1H, C*H*=CH-NH), 6.6–6.8 (m, 5H), 6.9 (d, *J* = 7.5 Hz, 1H), 7.1 (m, 1H), 7.2 (t, *J* = 7.6 Hz, 1H), 7.5 (t, *J* = 12.5 Hz, 1H, CH=C*H*-NH), 9.6 (d, *J* = 12.8 Hz, 1H, CH=CH-N*H*); ^13^C-NMR (75.46 MHz, DMSO-*d*_6_, *EEE*-**21f**) *δ* 13.1 (s, CH_2_-*C*H_3_), 13.3 (s, CH_2_-*C*H_3_), 24 (s, *C*H_2_-CH_3_), 24.3 (s, *C*H_2_-CH_3_), 90.2, 114.1, 117.8, 122, 123.1, 129, 129.2, 130.2, 140, 141.1 (t, *^3^J_CF_* = 5.9 Hz, CF_2_-(C=N)-*C*H=CH-NH), 151.1, 153.5 (t, *^2^J_C1F_* = 22.1 Hz, CF_2_-(*C*=N)-CH=CH-NH); ^19^F-NMR (282.4 MHz, DMSO-*d*_6_, *EEE*-**21f**) *δ −*81.5 (m, 3F), −109.1 (m, 2F), −120.2 (m, 2F), −121.5 (m, 2F), −126.1 (m, 2F). MS (*m*/*z*): 547 [M + H]^+^. HRMS calcd. for C_24_H_22_F_11_N_2_^+^: 547.1607, found: 547.1611; Anal calcd. for C_24_H_21_F_11_N_2_: C, 52.75; H, 3.87; N, 5.13, found C, 52.78; H, 3.88; N, 5.14. 

*3-perfluoropentyl-1-(4-ethylphenylamino)-3-(4-ethylphenylimino)-propene (***21g***)*. 4 g of 4-ethylaniline **15g** (3.3 × 10^−2^ mol) were added to a solution of 8.8 g (1.65 × 10^−2^ mol) of **14** (R_F_ = C_5_F_11_) in 88 mL of dry dichloromethane. The mixture was stirred for 4 h at room temperature. 7.67 g of the title product **21g** were obtained, total yield 85%. ^1^H-NMR (300.13 MHz, DMSO-*d*_6_, *EEE*-**21g**) *δ* 1.2 (t, *J* = 8 Hz, 3H, CH_2_-C*H*_3_), 1.3 (t, *J* = 8 Hz, 3H, CH_2_-C*H*_3_), 2.6 (q, *J* = 8 Hz, 2H, C*H*_2_-CH_3_), 2.7 (q, *J* = 8 Hz, 2H, C*H*_2_-CH_3_), 5.6 (d, *J* = 13.4 Hz, 1H, C*H*=CH-NH), 6.6 (d, *J* = 8.1 Hz, 2H), 6.8 (d, *J* = 8.2 Hz, 2H), 7.1 (d, *J* = 8.2 Hz, 2H), 7.2 (d, *J* = 8.1 Hz, 2H), 7.5 (t, *J* = 12.8 Hz, 1H, CH=C*H*-NH), 9.6 (d, *J* = 12.7 Hz, 1H, CH=CH-N*H*); ^13^C-NMR (75.46 MHz, DMSO-*d*_6_, *EEE*-**21g**) *δ* 13.3 (s, CH_2_-*C*H_3_), 13.4 (s, CH_2_-*C*H_3_), 24.3 (s, *C*H_2_-CH_3_), 24.5 (s, *C*H_2_-CH_3_), 90.2, 115.5, 117.5, 123.1, 125.2, 129.1, 129.3, 140.5, 141.9 (t, *^3^J_CF_* = 4.2 Hz, CF_2_-(C=N)-*C*H=CH-NH), 151.5, 153.4 (t, *^2^J_C1F_* = 32.2 Hz, CF_2_-(*C*=N)-CH=CH-NH); ^19^F-NMR (282.4 MHz, DMSO-*d*_6_, *EEE*-**21g**) *δ −*80.7 (m, 3F), −109.5 (m, 2F), −120.7 (m, 2F), −122.4 (m, 2F), −125.8 (m, 2F). MS (*m*/*z*): 547 [M + H]^+^. HRMS calcd. for C_24_H_22_F_11_N_2_^+^: 547.1607, found: 547.1609; Anal calcd. for C_24_H_21_F_11_N_2_: C, 52.75; H, 3.87; N, 5.13, found C, 52.77; H, 3.87; N, 5.14.

*3-Trifluoromethyl-1-(4-ethylphenylamino)-3-(4-ethylphenylimino)-propene (***21g’***)*. 6.56 g of 4-ethylaniline **15g** (5.42 × 10^−2^ mol) were added to a solution of 9 g (2.71 × 10^−2^ mol) of **14’** (R_F_ = CF_3_) in 90 mL of dry dichloromethane. The mixture was stirred for 4 h at room temperature. 7.97 g of the title product **21g’** were obtained, total yield 85%. ^1^H-NMR (300.13 MHz, DMSO-*d*_6_, *EEE*-**21g’**) *δ* 1.2 (t, *J* = 7.8 Hz, 3H, CH_2_-C*H*_3_), 1.3 (t, *J* = 8 Hz, 3H, CH_2_-C*H*_3_), 2.5 (q, *J* = 7.9 Hz, 2H, C*H*_2_-CH_3_), 2.7 (q, *J* = 8.1 Hz, 2H, C*H*_2_-CH_3_), 5.5 (d, *J* = 13.6 Hz, 1H, C*H*=CH-NH), 6.7 (d, *J* = 7.9 Hz, 2H), 6.8 (d, *J* = 8 Hz, 2H), 7.1 (d, *J* = 8.1 Hz, 2H), 7.2 (d, *J* = 8 Hz, 2H), 7.5 (t, *J* = 12.5 Hz, 1H, CH=C*H*-NH), 9.6 (d, *J* = 12.4 Hz, 1H, CH=CH-N*H*); ^13^C-NMR (75.46 MHz, DMSO-*d*_6_, *EEE*-**21g’**) *δ* 13.3 (s, CH_2_-*C*H_3_), 13.4 (s, CH_2_-*C*H_3_), 24.3 (s, *C*H_2_-CH_3_), 24.5 (s, *C*H_2_-CH_3_), 90.6, 116.2, 120.1, 120.5 (q, *^1^J_CF_* = 280.5 Hz, CF_3_), 129.7, 129.9, 130.8, 131, 132.5, 138.3, 140.8 (q, *^3^J_CF_* = 4.2 Hz, CF_3_-(C=N)-*C*H=CH-NH), 147.5, 153.1 (q, *^2^J_CF_* = 29.9 Hz, CF_3_-(*C*=N)-CH=CH-NH); ^19^F-NMR (282.4 MHz, DMSO-*d*_6_, *EEE*-**21g’**) *δ −*65.5 (s, 3F). MS (*m*/*z*): 347 [M + H]^+^. HRMS calcd. for C_20_H_22_F_3_N_2_^+^: 347.1735, found: 347.1740; Anal calcd. for C_20_H_21_F_3_N_2_: C, 69.35; H, 6.11; N, 8.09, found C, 69.41; H, 6.10; N, 8.11.

*3-perfluoropentyl-1-(2-chlorophenylamino)-3-(2-chlorophenylimino)-propene (***21h***).* 4.84 g of 2-chloroaniline **15h** (3.79 × 10^−2^ mol) were added to a solution of 10.1 g (1.89 × 10^−2^ mol) of **14** (R_F_ = C_5_F_11_) in 101 mL of dry dichloromethane. The mixture was stirred for 4 h at room temperature. 6.57 g of the title product **21h** were obtained, total yield 62%. ^1^H-NMR (300.13 MHz, DMSO-*d*_6_, *EEE*-**21h**) *δ* 5.6 (d, *J* = 13.8 Hz, 1H, C*H*=CH-NH), 6.9 (d, *J* = 7.71 Hz, 1H_Ar_), 7.1–7.4 (m, 7H_Ar_), 7.5 (d, *J* = 7.9 Hz, 1H, CH=C*H*-NH), 9.5 (bs, 1H, CH=CH-N*H*); ^13^C-NMR (75.46 MHz, DMSO-*d*_6_, *EEE*-**21h**) *δ* 90.1, 114.2, 115.1, 117.1, 121.2, 129.3, 129.4, 130.5, 140.5, 149.9, 153.2 (t, *^2^J_C1F_* = 29.8 Hz, CF_2_-(*C*=N)-CH=CH-NH); ^19^F-NMR (282.4 MHz, DMSO-*d*_6_, *EEE*-**21h**) *δ −*80.6 (m, 3F), −109.5 (m, 2F), −120.3 (m, 2F), −121.1 (m, 2F), −125 (m, 2F). MS (*m*/*z*): 560 [M + H]^+^. HRMS calcd. for C_20_H_12_Cl_2_F_11_N_2_^+^: 559.0202, found: 559.0210; Anal calcd. for C_20_H_11_Cl_2_F_11_N_2_: C, 42.96; H, 1.98; N, 5.01, found C, 42.95; H, 1.96; N, 5.10.

*3-perfluoropentyl-1-(3-chlorophenylamino)-3-(3-chlorophenylimino)-propene (***21i***)*. 4.79 g of 3-chloroaniline **15i** (3.75 × 10^−2^ mol) were added to a solution of 10 g (1.87 × 10^−2^ mol) of **14** (R_F_ = C_5_F_11_) in 100 mL of dry dichloromethane. The mixture was stirred for 4 h at room temperature. 7.35 g of the title product **21i** were obtained, total yield 70%. ^1^H-NMR (300.13 MHz, DMSO-*d*_6_, *EEE*-**21i**) *δ* 5.6 (d, *J* = 13.6 Hz, 1H, C*H*=CH-NH), 6.7–6.9 (m, 6H), 7.2–7.3 (m, 2H), 7.5 (t, *J* = 13.2 Hz, 1H, CH=C*H*-NH), 9.7 (d, *J* = 12.8 Hz, 1H, CH=CH-N*H*); ^13^C-NMR (75.46 MHz, DMSO-*d*_6_, *EEE*-**21i**) *δ* 90.4, 112.2, 115.1, 116.4, 117.1, 121.4, 129.1, 129.5, 130.6, 141.5, 150.2, 153.2 (t, *^2^J_C1F_* = 30.2 Hz, CF_2_-(*C*=N)-CH=CH-NH); ^19^F-NMR (282.4 MHz, DMSO-*d*_6_, *EEE*-**21i**) *δ −*80.5 (m, 3F), −109.1 (m, 2F), −120.1 (m, 2F), −121.1 (m, 2F), −125 (m, 2F). MS (*m*/*z*): 560 [M + H]^+^. HRMS calcd. for C_20_H_12_Cl_2_F_11_N_2_^+^: 559.0202, found: 559.0205; Anal calcd. for C_20_H_11_Cl_2_F_11_N_2_: C, 42.96; H, 1.98; N, 5.01, found C, 42.98; H, 1.97; N, 5.00.

*3-perfluoropentyl-1-(4-chlorophenylamino)-3-(4-chlorophenylimino)-propene (***21j***)*. 4.6 g of 4-chloroaniline **15j** (3.6 × 10^−2^ mol) were added to a solution of 9.6 g (1.8 × 10^−2^ mol) of **14** (R_F_ = C_5_F_11_) in 96 mL of dry dichloromethane. The mixture was stirred for 4 h at room temperature. 7.76 g of the title product **21j** were obtained, total yield 77%. ^1^H-NMR (300.13 MHz, DMSO-*d*_6_, *EEE*-**21j**) *δ* 5.4 (d, *J* = 13.6 Hz, 1H, C*H*=CH-NH), 6.8 (d, *J* = 8 Hz, 2H), 7 (d, *J* = 8.1 Hz, 2H), 7.3 (d, *J* = 8.2 Hz, 2H), 7.4 (d, *J* = 8 Hz, 2H), 7.6 (t, *J* = 13.2 Hz, 1H, CH=C*H*-NH), 9.8 (d, *J* = 12.9 Hz, 1H, CH=CH-N*H*); ^13^C-NMR (75.46 MHz, DMSO-*d*_6_, *EEE*-**21j**) *δ* 90.3, 116.1, 117.5, 123, 125.2, 129.2, 129.3, 140.6, 141.5 (t, *^3^J_CF_* = 5.1 Hz, CF_2_-(C=N)-*C*H=CH-NH), 151.6, 153.5 (t, *^2^J_C1F_* = 31.1 Hz, CF_2_-(*C*=N)-CH=CH-NH); ^19^F-NMR (282.4 MHz, DMSO-*d*_6_, *EEE*-**21j**) *δ −*80.6 (m, 3F), −109.2 (m, 2F), −120.5 (m, 2F), −122.5 (m, 2F), −125.2 (m, 2F). MS (*m*/*z*): 560 [M + H]^+^. HRMS calcd. for C_20_H_12_Cl_2_F_11_N_2_^+^: 559.0202, found: 559.0209; Anal calcd. for C_20_H_11_Cl_2_F_11_N_2_: C, 42.96; H, 1.98; N, 5.01, found C, 42.99; H, 1.98; N, 5.03.

*3-perfluoropentyl-1-(2-methoxyphenylamino)-3-(2-methoxyphenylimino)-propene (***21k***).* 3.93 g of 2-methoxyaniline **15k** (3.19 × 10^−2^ mol) were added to a solution of 8.5 g (1.59 × 10^−2^ mol) of **14** (R_F_ = C_5_F_11_) in 85 mL of dry dichloromethane. The mixture was stirred for 6 h at room temperature. 5.97 g of the title product **21k** were obtained, total yield 68%. ^1^H-NMR (300.13 MHz, DMSO-*d*_6_, *EEE*-**21k**) *δ* 3.7 (s, 3H, OC*H*_3_), 3.8 (s, 3H, OC*H*_3_), 5.5 (d, *J* = 12.8 Hz, C*H*=CH-NH), 6.5–7.4 (m, 8H, H_Ar_), 7.5 (m, 1H, CH=C*H*-NH), 9.1 (bs, 1H, CH=CH-N*H*); ^13^C-NMR (75.46 MHz, DMSO-*d*_6_, *EEE*-**21k**) *δ* 55.5 (s, O*C*H_3_), 55.6 (s, O*C*H_3_), 89.2, 112.5, 116.9, 120.8, 129.1, 129.2, 130.2, 132.1, 140.6, 141.2 (t, *^3^J_CF_* = 4.5 Hz, CF_2_-(C=N)-*C*H=CH-NH), 149.2, 152.9 (t, *^2^J_C1F_* = 20.1 Hz, CF_2_-(*C*=N)-CH=CH-NH); ^19^F-NMR (282.4 MHz, DMSO-*d*_6_, *EEE*-**21k**) *δ −*80.6 (m, 3F), −109.1 (m, 2F), −120.1 (m, 2F), −121.3 (m, 2F), −125.5 (m, 2F). MS (*m*/*z*): 551 [M + H]^+^. HRMS calcd. for C_22_H_18_F_11_N_2_O_2_^+^: 551.1193, found: 551.1199; Anal calcd. for C_22_H_17_F_11_N_2_O_2_: C, 48.01; H, 3.11; N, 5.09, found C, 48.08; H, 3.10; N, 5.11.

*3-perfluoropentyl-1-(4-methoxyphenylamino)-3-(4-methoxyphenylimino)-propene (***21l***)*. 4.25 g of 4-anisidine **15l** (3.45 × 10^−2^ mol) were added to a solution of 9.2 g (1.72 × 10^−2^ mol) of **14** (R_F_ = C_5_F_11_) in 92 mL of dry dichloromethane. The mixture was stirred for 4 h at room temperature. 6.84 g of the title product **21l** were obtained, total yield 72%. ^1^H-NMR (300.13 MHz, DMSO-*d*_6_, *EEE*-**21l**) *δ* 3.7 (s, 3H, OC*H*_3_), 3.8 (s, 3H, OC*H*_3_), 5.6 (d, *J* = 13.1 Hz, 1H, C*H*=CH-NH), 6.6 (d, *J* = 7.9 Hz, 2H), 6.8 (d, *J* = 8 Hz, 2H), 7.1 (d, *J* = 8 Hz, 2H), 7.2 (d, *J* = 8.1 Hz, 2H), 7.5 (t, *J* = 12.5 Hz, 1H, CH=C*H*-NH), 9.4 (d, *J* = 12.7 Hz, 1H, CH=CH-N*H*); ^13^C-NMR (75.46 MHz, DMSO-*d*_6_, *EEE*-**21l**) *δ* 55.5 (s, O*C*H_3_), 55.7 (s, O*C*H_3_), 89.8, 114.8, 115.6, 117.52, 123.1, 125, 129, 129.3, 139.8, 141.5 (t, *^3^J_CF_* = 3.9 Hz, CF_2_-(C=N)-*C*H=CH-NH), 151.1, 154.2 (t, *^2^J_C1F_* = 29.6 Hz, CF_2_-(*C*=N)-CH=CH-NH); ^19^F-NMR (282.4 MHz, DMSO-*d*_6_, *EEE*-**21l**) *δ −*80.5 (m, 3F), −109 (m, 2F), −120.3 (m, 2F), −122.2 (m, 2F), −125.5 (m, 2F). MS (*m*/*z*): 551 [M + H]^+^. HRMS calcd. for C_22_H_18_F_11_N_2_O_2_^+^: 551.1193, found: 551.1195; Anal calcd. for C_22_H_17_F_11_N_2_O_2_: C, 48.01; H, 3.11; N, 5.09, found C, 48.05; H, 3.12; N, 5.08.

### 3.7. General Procedure for the Isolation of 2-Perfluoropentyl- and 2-Trifluoromethyl-1-((2-/4-)-Carboxy-phenyl)Amino-3-((2-/4-)-Carboxyphenyl)Iminopropene Intermediates ***21m***–***n’***

A mixture of one equivalent of gem-iodoacetate compounds **14** or **14’** and two equivalents of the corresponding aminobenzoic acid **15m–n** in dichloromethane (10 mL DCM for 1 g of **14**–**14’**) was stirred at reflux until disappearance of ^19^F-NMR signals corresponding to the starting products **14**–**14’** (2–4 h). The reaction mixture was concentrated in vacuo and then diluted with diethyl ether. An excess of petroleum ether was added, and the precipitate that had formed was eliminated by vacuum filtration. The filtrate was concentrated under reduced pressure to give brown oil. Chromatography over silica gel column (eluent, petroleum ether/ethyl acetate 90/10 *v*/*v*) then purification over a plate chromatography (eluent petroleum ether/ethyl acetate 70/30 *v*/*v*) yielded pure compounds **21m-n’** as yellow amorphous solids.

*3-perfluoropentyl-1-(2-carboxyphenylamino)-3-(2-carboxyphenylimino)-propene (***21m***).* 5.15 g of 2-aminobenzoic acid **15m** (3.75 × 10^−2^ mol) were added to a solution of 10 g (1.87 × 10^−2^ mol) of **14** (R_F_ = C_5_F_11_) in 100 mL of dry dichloromethane. The mixture was stirred for 4 h at reflux. 4.88 g of the title product **21m** were obtained after column chromatography, yield 45%. ^1^H-NMR (300.13 MHz, DMSO-*d*_6_, *EEE*-**21m**) *δ* 5.7 (d, *^3^J_HH_* = 13.7 Hz, C*H*=CH-NH), 6.9–7.45 (m, 8H, H_Ar_), 7.6 (m, 1H, CH=C*H*-NH), 9.6 (bs, 1H, CH=CH-N*H*), 14 (bs, 2H, CO_2_*H*); ^13^C-NMR (75.46 MHz, DMSO-*d*_6_, *EEE*-**21m**) *δ* 90.1, 114.5, 115, 118.1, 120.8, 129.1, 129.4, 131.2, 141.5, 150.5, 153.1 (t, *^2^J_C1F_* = 25.6 Hz, CF_2_-(*C*=N)-CH=CH-NH), 170.5 (s, *C*O_2_H), 170.7 (s, *C*O_2_H); ^19^F-NMR (282.4 MHz, DMSO-*d*_6_, *EEE*-**21m**) *δ −*80.3 (m, 3F), −109.68 (m, 2F), −120.4 (m, 2F), −121.05 (m, 2F), −125.83 (m, 2F). MS (*m*/*z*): 579 [M + H]^+^. HRMS calcd. for C_22_H_14_F_11_N_2_O_4_^+^: 579.0778, found: 579.0783 Anal calcd. for: C_22_H_13_F_11_N_2_O_4_: C, 45.69; H, 2.27; N, 4.84, found C, 45.72; H, 2.26; N,4.82. 

*3-Trifluoromethyl-1-(2-carboxyphenylamino)-3-(2-carboxyphenylimino)-propene (***21m’***)*. 6.44 g of 2-aminobenzoic acid **15m** (4.69 × 10^−2^ mol) were added to a solution of 7.8 g (2.34 × 10^−2^ mol) of **14’** (R_F_ = CF_3_) in 78 mL of dry dichloromethane. The mixture was stirred for 4 h at reflux. 3.99 g of the title product **21m’** were obtained after column chromatography, yield 45%. ^1^H-NMR (300.13 MHz, DMSO-*d*_6_, *EEE*-**21m’**) *δ* 5.7 (d, *J* = 13.6 Hz, C*H*=CH-NH), 6.8–7.5 (m, 8H, H_Ar_), 7.6 (m, 1H, CH=C*H*-NH), 9.7 (bs, 1H, CH=CH-N*H*), 14.1 (bs, 2H, CO_2_*H*); ^13^C-NMR (75.46 MHz, DMSO-*d*_6_, *EEE*-**21m’**) *δ* 91.4, 115.2, 119.5, 121 (q, *^1^J_CF_* = 277.7 Hz, CF_3_), 129.2, 129.7, 130.1, 130.8, 131.5, 132.5, 138.1, 140.7 (q, *^3^J_CF_* = 2.8 Hz, CF_3_-(C=N)-*C*H=CH-NH), 147.1, 153.5 (q, *^2^J_CF_* = 30.1 Hz, CF_3_-(*C*=N)-CH=CH-NH), 170.3 (s, *C*O_2_H), 170.5 (s, *C*O_2_H); ^19^F-NMR (282.4 MHz, DMSO-*d*_6_, *EEE*-**21m’**) *δ −*65.6 (s, 3F). MS (*m*/*z*): 379 [M + H]^+^. HRMS calcd. for C_18_H_14_F_3_N_2_O_4_^+^: 379.0906, found: 379.0911 Anal calcd. for: C_18_H_13_F_3_N_2_O_4_: C, 57.15; H, 3.46; N, 7.41, found C, 57.19; H, 3.45; N,7.40.

*3-Perfluoropenthyl-1-(4-carboxyphenyl)amino-3-(4-carboxyphenyl)iminopropene (***21n***)*. 5.15 g of 4-aminobenzoic acid (3.75 × 10^−2^ mole) were added to a solution of 10 g (1.87 × 10^−2^ mole) of **14** (R’_F_ = C_6_F_13_) in 100 mL of dry dichloromethane. The mixture was stirred for 4 h at reflux. 5.21 g of the title product **21n** were obtained after column chromatography, yield 48%. ^1^H-NMR (300.13 MHz, DMSO-*d*_6_, *EEE*-**21n**) *δ* 5.4 (d, *^3^J_HH_* = 12.9 Hz, 1H, C*H*=CH-NH), 6.8 (d, *^3^J_HH_* = 8.1 Hz, 2H, Ar-*H*), 7 (d, *^3^J_HH_* = 8.3 Hz, 2H, Ar-*H*), 7.3 (d, *^3^J_HH_* = 8.3 Hz, 2H, Ar-*H*), 7.4 (d, *^3^J_HH_* = 8.1 Hz, 2H, Ar-*H*), 7.6 (t (dd), *^3^J_HH_* = *^3^J_HNH_* = 12.5 Hz, 1H, CH=C*H*-NH), 9.9 (d, *^3^J_HNH_* = 12.2 Hz, CH=CH-N*H*), 13.5 (bs, 2H, CO_2_*H*); ^13^C-NMR (75.46 MHz, DMSO-*d*_6_, *EEE*-**21n**) *δ* 91, 115.5, 118.5, 121.9, 123.5, 129.2, 129.5, 130.1, 142.5, 141.9 (t, *^3^J_CF_* = 4.9 Hz, CF_2_-(C=N)-*C*H=CH-NH), 149.8, 153.8 (t, *^2^J_C1F_* = 20.5 Hz, CF_2_-(*C*=N)-CH=CH-NH); 170.1 (s, *C*O_2_H), 170.4 (s, *C*O_2_H); ^19^F-NMR (282.4 MHz, DMSO-*d*_6_, *EEE*-**21n**) *δ −*80.5 (m, 3F, CF_2_-CF_2_-CF_2_-CF_2_-CF_3_), −109.2 (t, *J* = 11.2 Hz, 2F, CF_2_-CF_2_-CF_2_-CF_2_-CF_3_), −120.4 (m, 2F, CF_2_-CF_2_-CF_2_-CF_2_-CF_3_), −121.5 (m, 2F, CF_2_-CF_2_-CF_2_-CF_2_-CF_3_), −125.5 (m, 2F, CF_2_-CF_2_-CF_2_-CF_2_-CF_3_). MS (*m*/*z*): 579 [M + H]^+^. HRMS calcd. for C_22_H_14_F_11_N_2_O_4_^+^: 579.0778, found: 579.0782 Anal calcd. for: C_22_H_13_F_11_N_2_O_4_: C, 45.69; H, 2.27; N, 4.84, found C, 45.71; H, 2.26; N,4.82. 

*3-Trifluoromethyl-1-(4-carboxyphenylamino)-3-(4-carboxyphenylimino)-propene (***21n’***)*. 8.67 g of 4-aminobenzoic acid **15n** (6.32 × 10^−2^ mol) were added to a solution of 10.5 g (3.16 × 10^−2^ mol) of **14** (R_F_ = CF_3_) in 105 mL of dry dichloromethane. The mixture was stirred for 4 h at reflux. 6.57 g of the title product **21n’** were obtained after column chromatography, yield 55%. ^1^H-NMR (300.13 MHz, DMSO-*d*_6_, *EEE*-**21n’**) *δ* 5.5 (d, *^3^J_HH_* = 13 Hz, 1H, C*H*=CH-NH), 6.8 (d, *J* = 8 Hz, 2H), 7.1 (d, *J* = 8.1 Hz, 2H), 7.3 (d, *J* = 8 Hz, 2H), 7.4 (d, *J* = 8.1 Hz, 2H), 7.6 (t, *J* = *J* = 12.8 Hz, 1H, CH=C*H*-NH), 9.8 (d, *J* = 12.5 Hz, CH=CH-N*H*), 13.8 (bs, 2H, CO_2_*H*); ^13^C-NMR (75.46 MHz, DMSO-*d*_6_, *EEE*-**21n’**) *δ* 91.1, 115.5, 119.8, 121.5 (q, CF_3_, *^1^J_CF_* = 278.2 Hz), 123.1, 123.5, 128.9, 129.2, 140.5, 141.2 (q, *^3^J_CF_* = 2.1 Hz, CF_3_-(C=N)-*C*H=CH-NH), 150.2, 153.5 (q, *^2^J_CF_* = 27.5 Hz, CF_3_-(*C*=N)-CH=CH-NH), 170 (s, *C*O_2_H), 170.2 (s, *C*O_2_H); ^19^F-NMR (282.4 MHz, DMSO-*d*_6_, *EEE*-**21n’**) *δ −*65.5 (s, 3F). MS (*m*/*z*): 379 [M + H]^+^. HRMS calcd. for C_18_H_14_F_3_N_2_O_4_^+^: 379.0906, found: 379.0910 Anal calcd. for: C_18_H_13_F_3_N_2_O_4_: C, 57.15; H, 3.46; N, 7.41, found C, 57.19; H, 3.45; N,7.42.

### 3.8. General Procedure for the Preparation and Isolation of 2-Perfluoropentyl- and 2-Trifluoromethyl-1-(3-Methoxyphenyl)Amino-3-(3-Methoxyphenyl)Iminopropene Intermediates ***21o***–***o’***

A mixture of one equivalent of *gem*-iodoacetate compounds **14** or **14’** and two equivalents of 3-anisidine **15o** in dichloromethane (20 mL DCM for 1 g of **14**–**14’**) was stirred at room temperature. The evolution of the reaction was monitored by TLC (eluent petroleum ether/ethyl acetate: 80/20 *v*/*v*), and ^19^F-NMR spectroscopy of aliquots. After four h of stirring, a solution of 10% sodium thiosulfate was added to the reaction mixture and the product was extracted three times with ether. The combined extracts were washed two times with saturated sodium bicarbonate solution, dried over anhydrous sodium sulfate and concentrated under reduced pressure to give a yellow oil. Chromatography over silica gel column (eluent: petroleum ether/ethyl acetate 98/2 *v*/*v*), then purification over a plate chromatography (eluent petroleum ether/ethyl acetate 85/15 *v*/*v*) yielded the pure compounds **21o**–**o’** as yellow liquids.

*3-Perfluoropenthyl-1-(3-methoxyphenyl)amino-3-(3-methoxyphenyl)iminopropene (***21o***).* 3.98 g of 3-anisidine **15o** (3.23 × 10^−2^ mole) were added to a solution of 8.6 g (1.61 × 10^−2^ mole) of **14** (R_F_ = C_5_F_11_) in 172 mL of dry dichloromethane. 4 g of the title product **21o** were obtained after column chromatography, yield 45%. ^1^H-NMR (300.13 MHz, DMSO-*d*_6_, *EEE*-**21o**) *δ* 3.7 (s, 3H, OC*H*_3_), 3.8 (s, 3H, OC*H*_3_), 5.4 (d, *J* = 13.1 Hz, 1H, C*H*=CH-NH), 6.6–6.9 (m, 6H, Ph-*H*), 7.1 (t, *J* = 7.4 Hz, 1H, Ph-*H*), 7.3 (t, *J* = 7.4 Hz, 1H, Ph-*H*), 7.6 (t, *J* = 12.9 Hz, 1H, CH=C*H*-NH), 9.6 (d, *J* = 12.8 Hz, 1H, CH=CH-N*H*); ^13^C-NMR (75.46 MHz, DMSO-*d*_6_, *EEE*-**21o**) *δ* 55.5 (s, O*C*H_3_), 55.6 (s, O*C*H_3_), 89.5, 113.5, 115.5, 117.5, 121.5, 123.5, 129.1, 129.4, 130.6, 140, 140.8 (t, *^3^J_CF_* = 4.2 Hz, CF_2_-(C=N)-*C*H=CH-NH), 150.5, 153.2 (t, *^2^J_C1F_* = 26.7 Hz, CF_2_-(*C*=N)-CH=CH-NH); ^19^F-NMR (282.4 MHz, DMSO-*d*_6_, *EEE*-**21o**) *δ −*80.5 (m, 3F, CF_2_-CF_2_-CF_2_-CF_2_-CF_3_), −109.5 (m, 2F, CF_2_-CF_2_-CF_2_-CF_2_-CF_3_), −120.5 (m, 2F, CF_2_-CF_2_-CF_2_-CF_2_-CF_3_), −121.3 (m, 2F, CF_2_-CF_2_-CF_2_-CF_2_-CF_3_), −125.6 (m, 2F, CF_2_-CF_2_-CF_2_-CF_2_-CF_3_). MS (*m*/*z*): 551 [M + H]^+^. HRMS calcd. for C_22_H_18_F_11_N_2_O_2_^+^: 551.1193, found: 551.1198; Anal calcd. for C_22_H_17_F_11_N_2_O_2_: C, 48.01; H, 3.11; N, 5.09, found C, 48.03; H, 3.11; N, 5.10.

*3-Trifluoromethyl-1-(3-methoxyphenyl)amino-3-(3-methoxyphenyl)iminopropene (***21o’***).* 6.67 g of 3-anisidine **15o** (5.42 × 10^−2^ mol) were added to a solution of 9 g (2.71 × 10^−2^ mol) of **14’** (R_F_ = CF_3_) in 180 mL of dry dichloromethane. 4.55 g of the title product **21o’** were obtained after column chromatography, yield 48%. ^1^H-NMR (300.13 MHz, DMSO-*d*_6_, *EEE*-**21o’**) *δ* 3.7 (s, 3H, OC*H*_3_), 3.8 (s, 3H, OC*H*_3_), 5.4 (d, *J* = 13 Hz, 1H, C*H*=CH-NH), 6.7–6.9 (m, 6H, Ph-*H*), 7.1 (t, *J* = 7.3 Hz, 1H, Ph-*H*), 7.3 (t, *J* = 7.4 Hz, 1H, Ph-*H*), 7.7 (t, *J* = 12.7 Hz, 1H, CH=C*H*-NH), 9.6 (d, *J* = 12.6 Hz, 1H, CH=CH-N*H*); ^13^C-NMR (75.46 MHz, DMSO-*d*_6_, *EEE*-**21o’**) *δ* 55.5 (s, O*C*H_3_), 55.6 (s, O*C*H_3_), 90.2, 113.1, 115.5, 116.5, 119.4, 120.7 (q, *^1^J_CF_* = 278.7 Hz, *C*F_3_), 123.5, 124.2, 129.6, 138.7, 139.1, 140.5, 141 (q, *^3^J_CF_* = 2.8 Hz, CF_3_-(C=N)-*C*H=CH-NH), 149.6, 153.5 (q, *^2^J_CF_* = 30.1 Hz, CF_3_-(*C*=N)-CH=CH-NH); ^19^F-NMR (282.4 MHz, DMSO-*d*_6_, *EEE*-**21o’**) *δ −*65.6 (s, 3F, C*F*_3_). MS (*m*/*z*): 351 [M + H]^+^. HRMS calcd. for C_18_H_18_F_3_N_2_O_2_^+^: 351.1320, found: 351.1322; Anal calcd. for C_18_H_17_F_3_N_2_O_2_: C, 61.71; H, 4.89; N, 8.00, found C, 61.75; H, 4.88; N, 8.01.

### 3.9. Reaction of N,N’-Diaryl-2-(Perfluoroalkyl)-1,5-Diazapentadienes ***21a***–***o’*** with Anilines ***15a***–***o*** and Ketones ***16t***–***v***: Formation of 2-Trifluoromethyl-/2-Perfluoroalkyl-N-Arylpyridinium Derivatives ***17***–***19*** or 2-Trifluoromethyl-/2-Perfluoroalkyl-7-Methoxyquinolines ***20o***–***o’***

To a stirred solution of *N,N’*-diaryl-2-(perfluoroalkyl)-1.5-diazapentadienes **21a**–**o’** (1 equiv.) in anhydrous dichloromethane (10 mL DCM for 1g of **21**), was added one equiv. of the corresponding substituted aniline **15a**–**o** and 1.2 equiv. of ketone **16t**–**v**. The mixture was stirred under reflux for desired time (4–12 h) until complete consumption of **21a**–**o’** (monitored by TLC eluent petroleum ether/ethyl acetate: 80/20 *v*/*v*, and ^19^F-NMR of aliquots). When the reaction was completed, the mixture was allowed to cool to r.t. then the brown precipitate accumulated during the reaction was separated by vacuum filtration (it was subsequently identified as anilinium salts by NMR and MS). 

Then in the cases of pyridinium iodides **17a**–**l**, **18a**–**l** and **19a**–**l**, ethyl ether was added to the filtrate. However, in the cases of pyridinium iodides **17m**–**n’**, **18m**–**n** and **19m**, a mixture of petroleum ether and ethyl ether (40/60 *v*/*v*) was added to the corresponding filtrates and the expected pyridiniums **17a**–**n’**, **18a**–**n** and **19a**–**m** precipitate instantly and were isolated by vacuum filtration as amorphous solids.

In the case of 3-anisidine (**15o**) at the end of the reaction, the mixture was concentrated under reduced pressure and then stirred with diethyl ether. An excess of petroleum ether was added; the precipitate that had formed was eliminated by vacuum filtration and washed three times with petroleum ether. The filtrate was concentrated in vacuo to give a yellow oil. Chromatography over silica gel column (eluent, petroleum ether/ethyl acetate 98/2 *v*/*v*) left a yellow oil which was crystallized from methanol/water to give pure samples of the corresponding quinolines **20o**–**o’**. All the isolated pyridiniums **17a**–**n’**, **18a**–**n**, **19a**–**m** and quinolines **20o**–**o’** were fully characterized and found identical to the previously obtained products. 

## 4. Conclusions

We have developed a new simple and efficient method for the synthesis of substituted 2-trifluoromethyl-/2-perfluoroalkyl-*N*-arylpyridiniums **17a**–**n’**, 2-trifluoromethyl-/2-perfluoroalkyl-*N*-(R-phenyl)-5,6,7,8-tetrahydroquinoliniums **18a**–**n** and 2-trifluoromethyl-/2-perfluoroalkyl-*N*-(R-phenyl)-6,7,8,9-tetrahydro-5*H*-cyclohepta[*b*]pyridiniums **19a**–**m**, starting from perfluoroalkylated *gem*-iodoacetoxy derivative **14**–**14”**, substituted anilines **15a**–**o** and ketones **16t**–**v**, under mild conditions with good to excellent yields. To our knowledge this is the first synthesis of pyridinium derivatives by a multicomponent reaction involving an activated β-dicarbonyl compound, a ketone, and an aromatic amine.

The reaction is assumed to proceed by a cascade cyclisation mechanism [66,67,68,69,70], either through an inverse electron demand Diels-Alder (IEDDA) cycloaddition, or through an Aza-Robinson cyclisation (Scheme 7), both ways being supported by the isolation and characterization of the intermediate *N*,*N’*-diaryl-2-(perfluoroalkyl)-1,5-diazapentadiene **21a**–**o’**, while stereoelectronic effects may account for the preferred intramolecular diazapentadiene cyclization in the case of the *m*-methoxy substituent, selectively forming the perfluoroalkylated quinoline **20**.

Beyond the scope of this work, various literature reports point out such hetero-Diels-Alder reactions as possibly relevant of bio-orthogonal chemistry because of their selectivity, moderate activation energy and ability to proceed under biological conditions, taking in account e.g., the pH and temperature [66,67,68,69,70]. Besides, since pyridinium-containing compounds are considered as possible exogenous neurotoxins, research efforts have also been undertaken to disclose the formation of such compounds under endogenous (biogenic) conditions [33,34,35,36,37,43,44,45]. In this respect, the biological activity of newly synthesized pyridinium compounds **17**–**19**, are currently under evaluation with promising results to be published in future papers.

## Figures and Tables

**Table 1:**
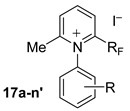
Reaction conditions and conversions for the synthesis of substituted 2-trifluoromethyl-/2-perfluoroalkyl-*N*-arylpyridinium compounds **17a**–**n’**, using acetone **16t** as reactant.

Entry	14		15		Time	Pyridiniums 17	Conv.
		R_F_		R	(h)		[%]^a^
1	**14**	**C_5_F_11_**	**15a**	H	4	**17a**	95
2	**14’**	**CF_3_**	**15a**	H	4	**17a’**	95
3	**14’’**	**C_3_F_7_**	**15a**	H	4	**17a’’**	95
4	**14**	**C_5_F_11_**	**15b**	*o*-Me	6	**17b**	80
5	**14**	**C_5_F_11_**	**15c**	*m*-Me	4	**17c**	85
6	**14**	**C_5_F_11_**	**15d**	*p*-Me	4	**17d**	88
7	**14’**	**CF_3_**	**15d**	*p*-Me	4	**17d’**	88
8	**14**	**C_5_F_11_**	**15e**	*o*-Et	6	**17e**	75
9	**14**	**C_5_F_11_**	**15f**	*m*-Et	4	**17f**	80
10	**14**	**C_5_F_11_**	**15g**	*p*-Et	4	**17g**	83
11	**14’**	**CF_3_**	**15g**	*p*-Et	4	**17g’**	85
12	**14**	**C_5_F_11_**	**15h**	*o*-Cl	12	**17h**	65
13	**14**	**C_5_F_11_**	**15i**	*m*-Cl	6	**17i**	75
14	**14**	**C_5_F_11_**	**15j**	*p*-Cl	6	**17j**	83
15	**14**	**C_5_F_11_**	**15k**	*o*-OMe	6	**17k**	75
16	**14**	**C_5_F_11_**	**15l**	*p*-OMe	4	**17l**	78
17	**14**	**C_5_F_11_**	**15m**	*o*-CO_2_H	12	**17m**	50
18	**14’**	**CF_3_**	**15m**	*o*-CO_2_H	12	**17m’**	52
19	**14**	**C_5_F_11_**	**15n**	*p*-CO_2_H	12	**17n**	60
20	**14’**	**CF_3_**	**15n**	*p*-CO_2_H	12	**17n’**	62

^a^ Determined by ^19^F-NMR analysis; NMR yield based on consumed **14** and formed **17**.

**Table 2:**
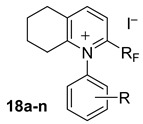
Reaction conditions and conversions for the preparation of substituted 2-trifluoromethyl-/2-perfluoroalkyl-*N*-(R-phenyl)-5,6,7,8-tetrahydroquinoliniums **18a**–**n**, using cyclohexanone **16u** as reactant.

Entry	14		15		Time	Pyridiniums 18	Conv.
		R_F_		R	(h)		[%]^a^
1	**14**	**C_5_F_11_**	**15a**	H	4	**18a**	85
2	**14’**	**CF_3_**	**15a**	H	4	**18a’**	88
3	**14**	**C_5_F_11_**	**15b**	*o*-Me	6	**18b**	85
4	**14**	**C_5_F_11_**	**15c**	*m*-Me	4	**18c**	85
5	**14**	**C_5_F_11_**	**15d**	*p*-Me	4	**18d**	85
6	**14**	**C_5_F_11_**	**15g**	*p*-Et	4	**18g**	86
7	**14**	**C_5_F_11_**	**15i**	*m*-Cl	6	**18i**	78
8	**14**	**C_5_F_11_**	**15l**	*p*-OMe	6	**18l**	90
9	**14**	**C_5_F_11_**	**15m**	*o*-CO_2_H	12	**18m**	75
10	**14**	**C_5_F_11_**	**15n**	*p*-CO_2_H	12	**18n**	75

^a^ Determined by ^19^F-NMR analysis; NMR yield based on consumed **14** and formed **18**.

**Table 3:**
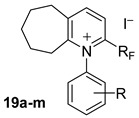
Reaction conditions and conversions for the preparation of 2-trifluoromethyl-/2-perfluoroalkyl-*N*-(R-phenyl)-6,7,8,9-tetrahydro-5*H*-cyclohepta[*b*]pyridiniums **19a**–**m**, using cyclo-heptanone **16v** as reactant.

Entry	14		15		Time	Pyridiniums 19	Conv.
		R_F_		R	(h)		[%]^a^
1	**14**	**C_5_F_11_**	**15a**	H	4	**19a**	85
2	**14**	**C_5_F_11_**	**15c**	*m*-Me	4	**19c**	83
3	**14**	**C_5_F_11_**	**15d**	*p*-Me	4	**19d**	85
4	**14**	**C_5_F_11_**	**15f**	*m*-Et	4	**19f**	88
5	**14**	**C_5_F_11_**	**15g**	*p*-Et	4	**19g**	85
6	**14**	**C_5_F_11_**	**15i**	*m*-Cl	4	**19i**	70
7	**14**	**C_5_F_11_**	**15j**	*p*-Cl	4	**19j**	78
8	**14**	**C_5_F_11_**	**15k**	*o*-OMe	6	**19k**	72
9	**14**	**C_5_F_11_**	**15l**	*p*-OMe	4	**19l**	88
10	**14**	**C_5_F_11_**	**15m**	*o*-CO_2_H	12	**19m**	70

^a^ Determined by ^19^F-NMR analysis; NMR yield based on consumed **14** and formed **19**.

**Table 4:**
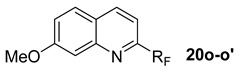
Reaction conditions and conversions obtained for the formation of substituted 2-perfluoropentyl-/2-trifluoromethyl-7-methoxyquinolines (**20o**–**o’**), starting from *m*-anisidine **15o**, with or without ketone **16t**–**v**.

Entry	14		16	Time	Conv.	Quinolines 20
		R_F_		(h)	[%]^a^	
1	**14**	**C_5_F_11_**	**16t**	12	85	**20o**
2	**14**	**C_5_F_11_**	**16u**	12	80	
3	**14**	**C_5_F_11_**	**16v**	12	82	
4	**14**	**C_5_F_11_**	**-**	12	86	
5	**14’**	**CF_3_**	**16t**	12	90	**20o’**
6	**14’**	**CF_3_**	**-**	12	92	

^a^ Determined by ^19^F-NMR analysis; NMR yield based on consumed **14** and formed **19**.

## Supplementary Materials

Supplementary Materials can be accessed.
