# Supplementary material for: Novel Synthesis of Substituted 2-Trifluoromethyl and 2-Perfluoroalkyl N-Arylpyridinium Compounds—Mechanistic Insights
Source: Molecules. 2019 Jun 25;24(12):2328. doi: 10.3390/molecules24122328 (PMC6630758; doi:10.3390/molecules24122328)
Supplement: Supplementary file 1 [file molecules-24-02328-s001.pdf]

Novel synthesis of substituted 2-trifluoromethyl and 2-perfluoroalkyl *N*-arylpyridinium compounds — mechanistic insights

Salem EL KHARRAT[a], Philippe LAURENT[b], Laurent BOITEAU[b], Hubert BLANCOU[b]

[a] Laboratoire de Recherche en Chimie Organique et Pharmaceutique, Faculté de Pharmacie et Faculté de Médecine, Université Saint-Joseph (USJ), Rue de Damas, Beirut, Lebanon.

e-mail : salem.kharrat@usj.edu.lb

[b] Institut des Biomolécules Max Mousseron, IBMM, UMR 5247, CNRS, Université de Montpellier, ENSCM ; c.c.1706, Place E. Bataillon 34095, Montpellier cedex 5, France

e-mails : philippe.laurent@umontpellier.fr

Supplementary Information : list of contents

Page 2 : Table S 1. List of described compounds **17–21**

Pages 3 to 53: Copies of ^{1}H, ^{13}C, ^{1}H-^{1}H COSY, ^{13}C-^{1}H HSQC and ^{19}F NMR spectrum of compounds : **17a, 17a', 17f, 17h, 17i, 17l, 17m, 17n, 18b, 18l, 18m, 19c, 19d, 19f, 19g, 19i, 19j, 19k, 19l, 19m**

Table S 1. List of described compounds **17–21** with correspondence to substrates **14**, **15**, **16** substitutions (including some examples previously published in refs 59, 64 of main Article). Examples of **17–21** are numbered after substrates **14–15** substitutions, e.g. **17a** is obtained from **14** and **15a**, or **17n′** is obtained from **14′** and **15n**

ketone (16)		Acetone 16t			Cyclohexanone 16u		Cycloheptanone 16v			
Substrate (14)		14′	14″	14	14′	14	14	14′	14″	14
R_F		CF_3	C_3F_7	C_5F_{11}	CF_3	C_5F_{11}	C_5F_{11}	CF_3	C_3F_7	C_5F_{11}
R (anilines 15)										
H	15a	17a′	17a″	17a	18a′	18a	19a	21a′[a]	21a″	21a[b]
o-Me	15b			17b		18b				21b
m-Me	15c			17c		18c	19c			21c
p-Me	15d	17d′		17d		18d	19d	21d′[a]		21d
o-Et	15e			17e						21e
m-Et	15f			17f			19f			21f
p-Et	15g	17g′		17g		18g	19g	21g′		21g
o-Cl	15h			17h						21h
m-Cl	15i			17i		18i	19i			21i
p-Cl	15j			17j			19j			21j
o-OMe	15k			17k			19k			21k
p-OMe	15l			17l		18l	19l			21l
o-CO_2H	15m	17m′		17m		18m	19m	21m′		21m
p-CO_2H	15n	17n′		17n		18n		21n′		21n
m-OMe	15o	20o′		20o		20o	20o	21o′		21o

[a] Spectroscopic characterizations published in ref. 64 of main Article. [b] Spectroscopic characterizations published in ref. 59 of main Article.

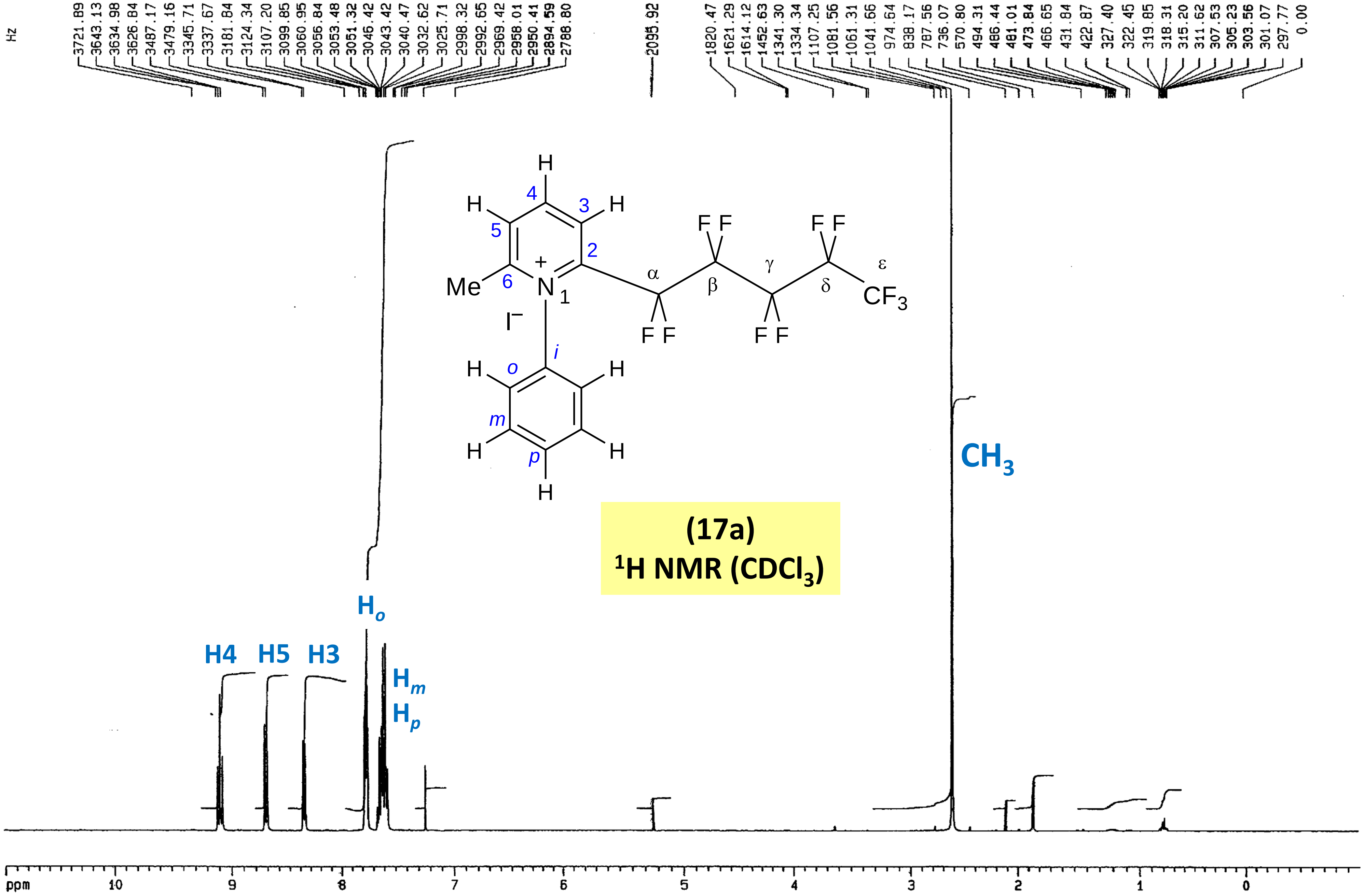

F2 - Acquisition Parameters

Date_	500000
Time	5.09
INSTRUM	spect
PROBHD	5 mm Dual 13
PULPROG	zg30
TD	32768
SOLVENT	CDCl3
NS	16
DS	2
SWH	8223.685 Hz
FIDRES	0.250967 Hz
AQ	1.9923444 sec
RG	161.3
DW	60.800 usec
DE	4.50 usec
TE	300.0 K
D1	1.00000000 sec
P1	10.00 usec
DE	4.50 usec
SFO1	400.1324710 MHz
NUC1	1H
PL1	-6.00 dB

F2 - Processing parameters

SI	16384
SF	400.1300289 MHz
WDW	EM
SSB	0
LB	0.30 Hz
GB	0
PC	1.00

1D NMR plot parameters

CX	30.00 cm
F1P	11.000 ppm
F1	4401.43 Hz
F2P	-1.000 ppm
F2	-400.13 Hz
PPMCM	0.40000 ppm/cm
HZCM	160.05202 Hz/cm

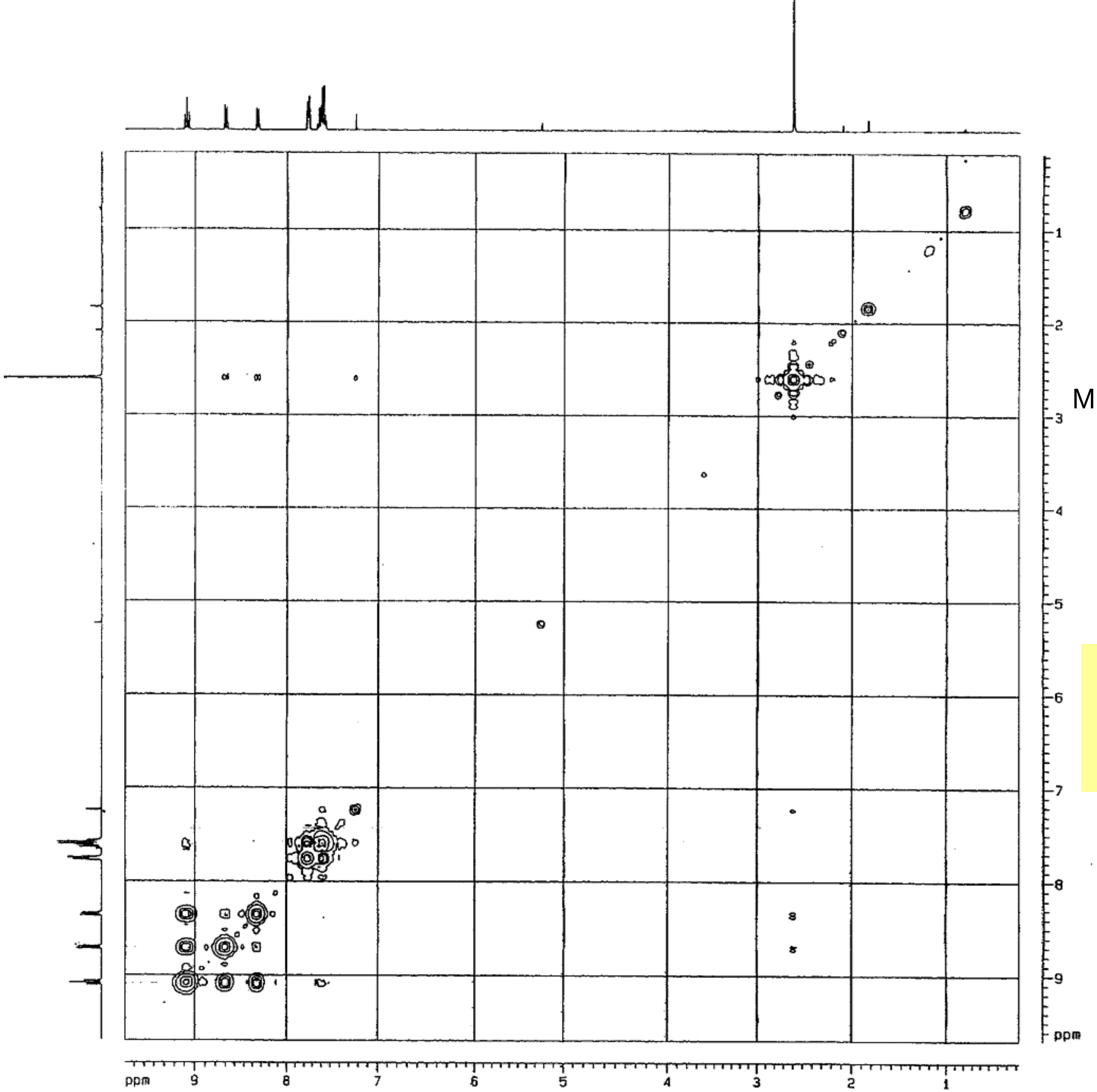

F2 - Acquisition Parameters

Date_	500000
Time	5.10
INSTRUM	spect
PROBHD	5 mm Dual 13
PULPROG	cosy45
TD	1024
SOLVENT	CDCl3

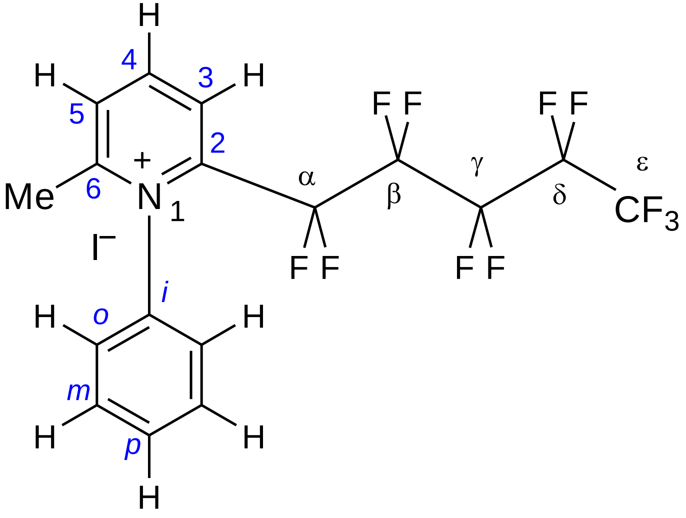

(17a)
COSY ^1H-^1H NMR
($CDCl_3$)

[illegible]	[illegible]
WDW	SINE
SSB	0
LB	0.00 Hz
GB	0

2D NMR plot parameters

CX2	20.00 cm
CX1	20.00 cm
F2PLO	9.698 ppm
F2LO	3880.29 Hz
F2PHI	0.173 ppm
F2HI	69.34 Hz
F1PLO	9.698 ppm
F1LO	3880.29 Hz
F1PHI	0.173 ppm
F1HI	69.34 Hz
F2PPMCM	0.47621 ppm/cm
F2HZCM	190.54784 Hz/cm
F1PPMCM	0.47621 ppm/cm
F1HZCM	[illegible]

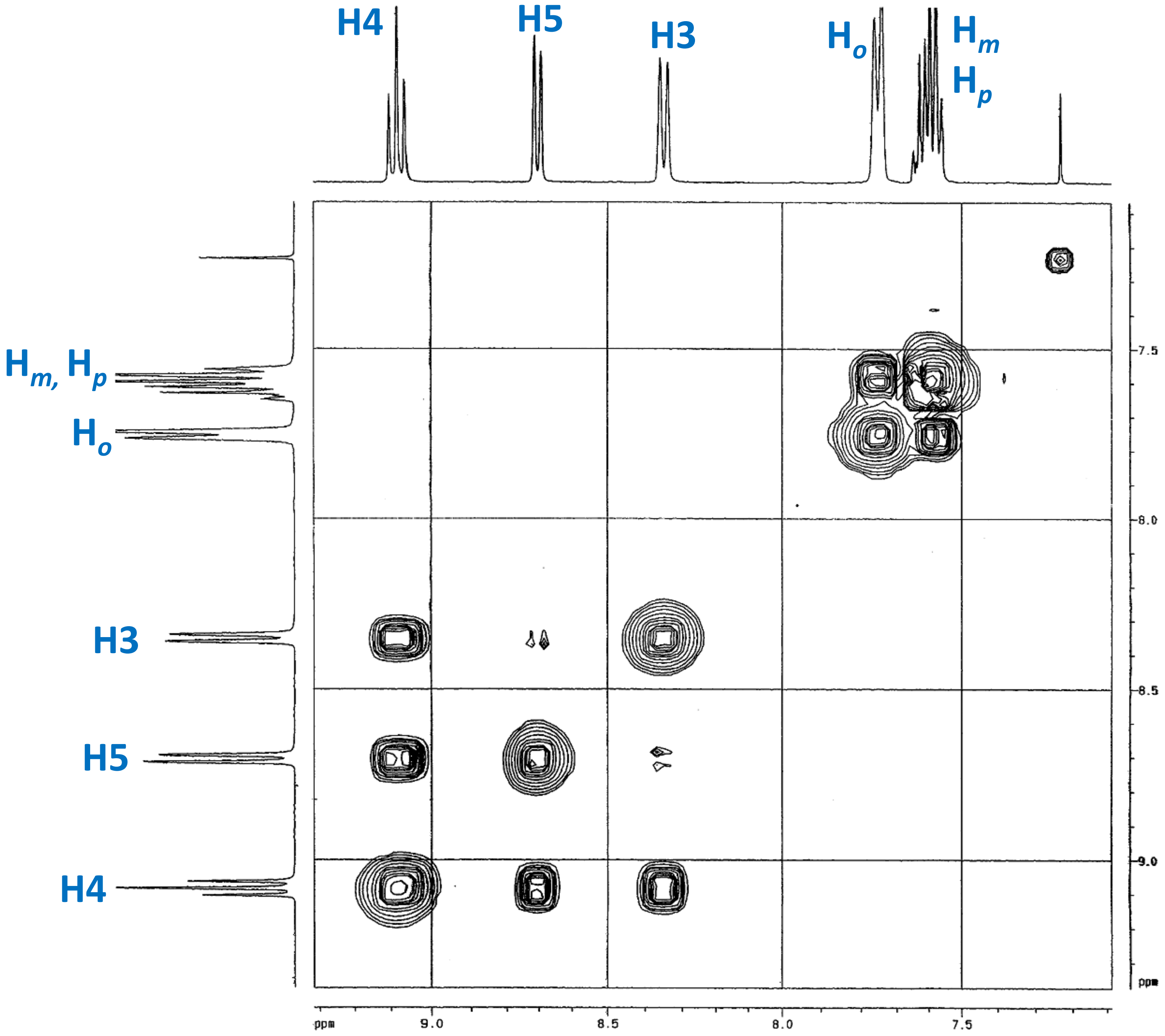

F2 - Acquisition Parameters

Date_	500000
Time	5.10
INSTRUM	spect
PROBHD	5 mm Dual 13
PULPROG	cosy45
TD	1024
SOLVENT	CDC13
NS	16
DS	4
SWH	3810.976 Hz
FIDRES	3.721656 Hz

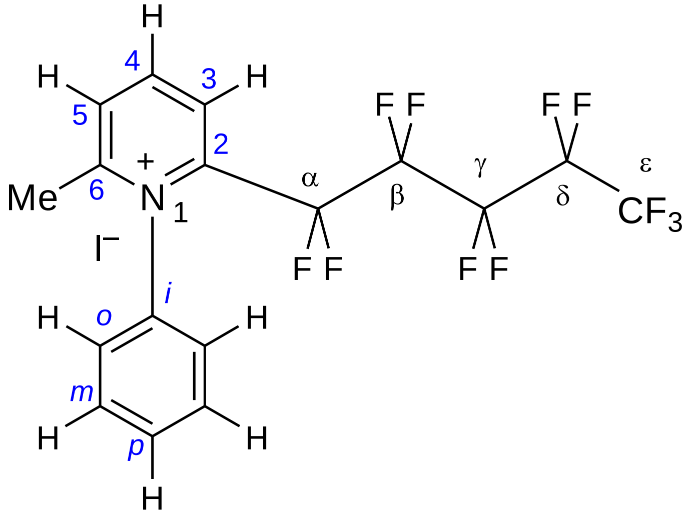

(17a)
COSY ^1H-^1H NMR
($CDCl_3$)

F2PHI	7.086 ppm
F2HI	2835.33 Hz
F1PLO	9.374 ppm
F1LO	3750.86 Hz
F1PHI	7.067 ppm
F1HI	2827.89 Hz
F2PPMCM	0.11161 ppm/cm
F2HZCM	44.65987 Hz/cm
F1PPMCM	0.11533 ppm/cm
F1HZCM	46.14854 Hz/cm

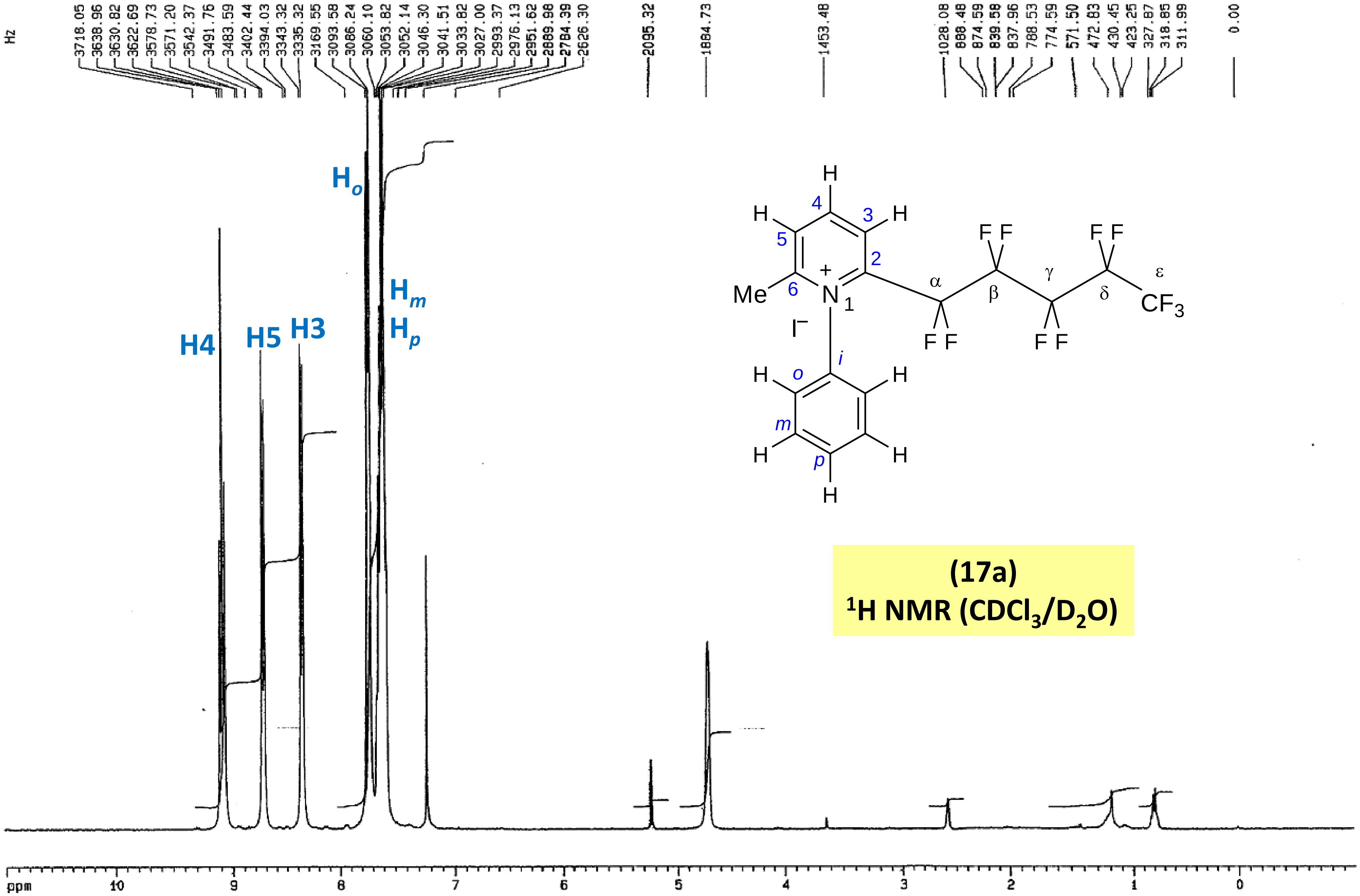

(17a)
^{1}H NMR ($CDCl_3$/D_2O)

F2 - Acquisition Parameters

Date_	500000
Time	10.43
INSTRUM	spect
PROBHD	5 mm Dual 13
PULPROG	zg30
TD	32768
SOLVENT	CDCl3
NS	16
DS	2
SWH	8223.685 Hz
FIDRES	0.250967 Hz
AQ	1.9923444 sec
RG	362
DW	60.800 usec
DE	4.50 usec
TE	300.0 K
D1	1.00000000 sec
P1	10.00 usec
DE	4.50 usec
SFO1	400.1324710 MHz
NUC1	1H
PL1	-6.00 dB

F2 - Processing parameters

SI	16384
SF	400.1300333 MHz
WDW	EM
SSB	0
LB	0.30 Hz
GB	0
PC	1.00

1D NMR plot parameters

CX	30.00 cm
F1P	11.000 ppm
F1	4401.43 Hz
F2P	-1.000 ppm
F2	-400.13 Hz
PPMCM	0.40000 ppm/cm
HZCM	160.05202 Hz/cm

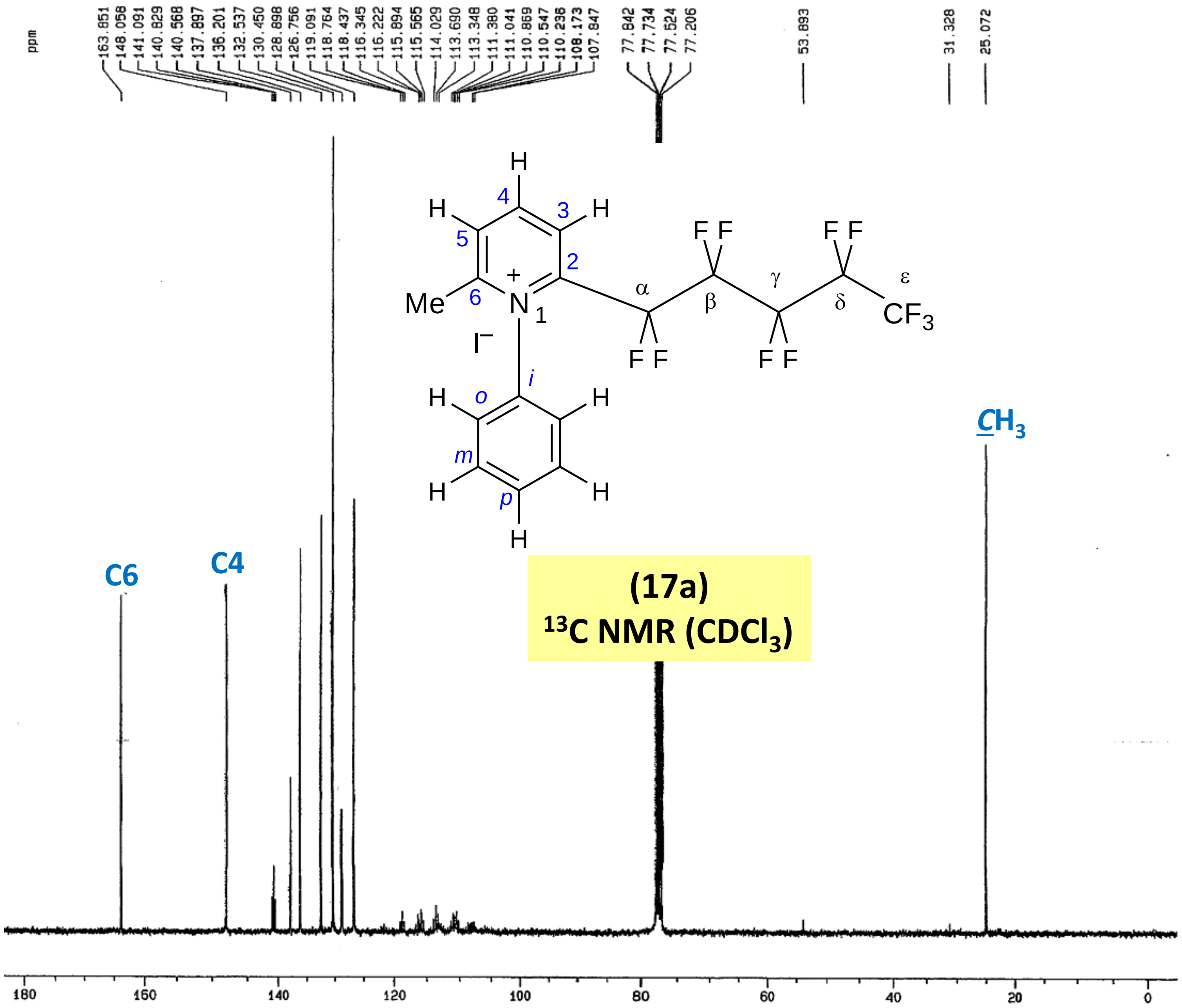

F2 - Acquisition Parameters	
Date_	500000
Time	9.52
INSTRUM	spect
PROBHD	5 mm Dual 13
PULPROG	zgpg30
TD	65536
SOLVENT	CDCl3
NS	6144
DS	2
SWH	31847.133 Hz
FIDRES	0.485949 Hz
AQ	1.0289652 sec
RG	8192
DW	15.700 usec
DE	4.50 usec
TE	300.0 K
D12	0.00002000 sec
PL13	20.00 dB
D1	1.00000000 sec
CPDPRG2	waltz16
PCPD2	100.00 usec
SFO2	400.1316005 MHz
NUC2	1H
PL2	-6.00 dB
PL12	17.00 dB
P1	6.00 usec
DE	4.50 usec
SFO1	100.6254358 MHz
NUC1	13C
PL1	-6.00 dB
D11	0.03000000 sec

F2 - Processing parameters	
SI	32768
SF	100.6127290 MHz
WDW	EM
SSB	0
LB	1.00 Hz
GB	0
PC	1.40

1D NMR plot parameters	
CX	30.00 cm
F1P	215.000 ppm
F1	21631.74 Hz
F2P	-5.000 ppm
F2	-503.06 Hz
PPMCM	7.33333 ppm/cm
HZCM	737.82672 Hz/cm

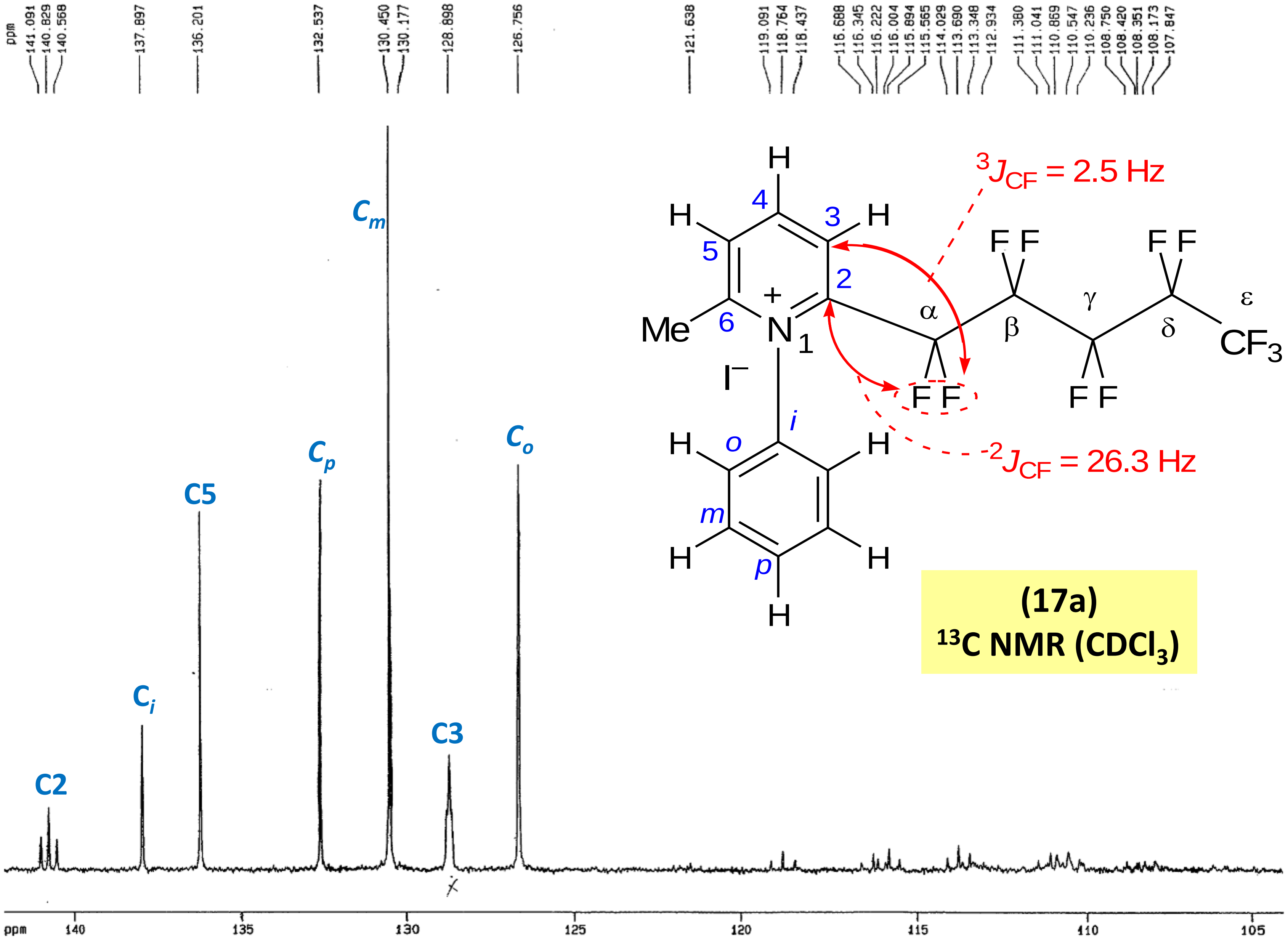

F2 - Acquisition Parameters

Date_	500000
Time	9.52
INSTRUM	spect
PROBHD	5 mm Dual 13
PULPROG	zgpg30
TD	65536
SOLVENT	CDCl3
NS	6144
DS	2
SWH	31847.133 Hz
FIDRES	0.485949 Hz
AQ	1.0289652 sec
RG	8192
DW	15.700 usec
DE	4.50 usec
TE	300.0 K
D12	0.00002000 sec
PL13	20.00 dB
D1	1.00000000 sec
CPDPRG2	waltz16
PCPD2	100.00 usec
SFO2	400.1316005 MHz
NUC2	1H
PL2	-6.00 dB
PL12	17.00 dB
P1	6.00 usec
DE	4.50 usec
SFO1	100.6254358 MHz
NUC1	13C
PL1	-6.00 dB
D11	0.03000000 sec

F2 - Processing parameters

SI	32768
SF	100.6127290 MHz
WDW	EM
SSB	0
LB	1.00 Hz
GB	0
PC	1.40

1D NMR plot parameters

CX	30.00 cm
F1P	142.219 ppm
F1	14309.00 Hz
F2P	104.194 ppm
F2	10483.24 Hz
PPMCM	1.26749 ppm/cm
HZCM	127.52564 Hz/cm

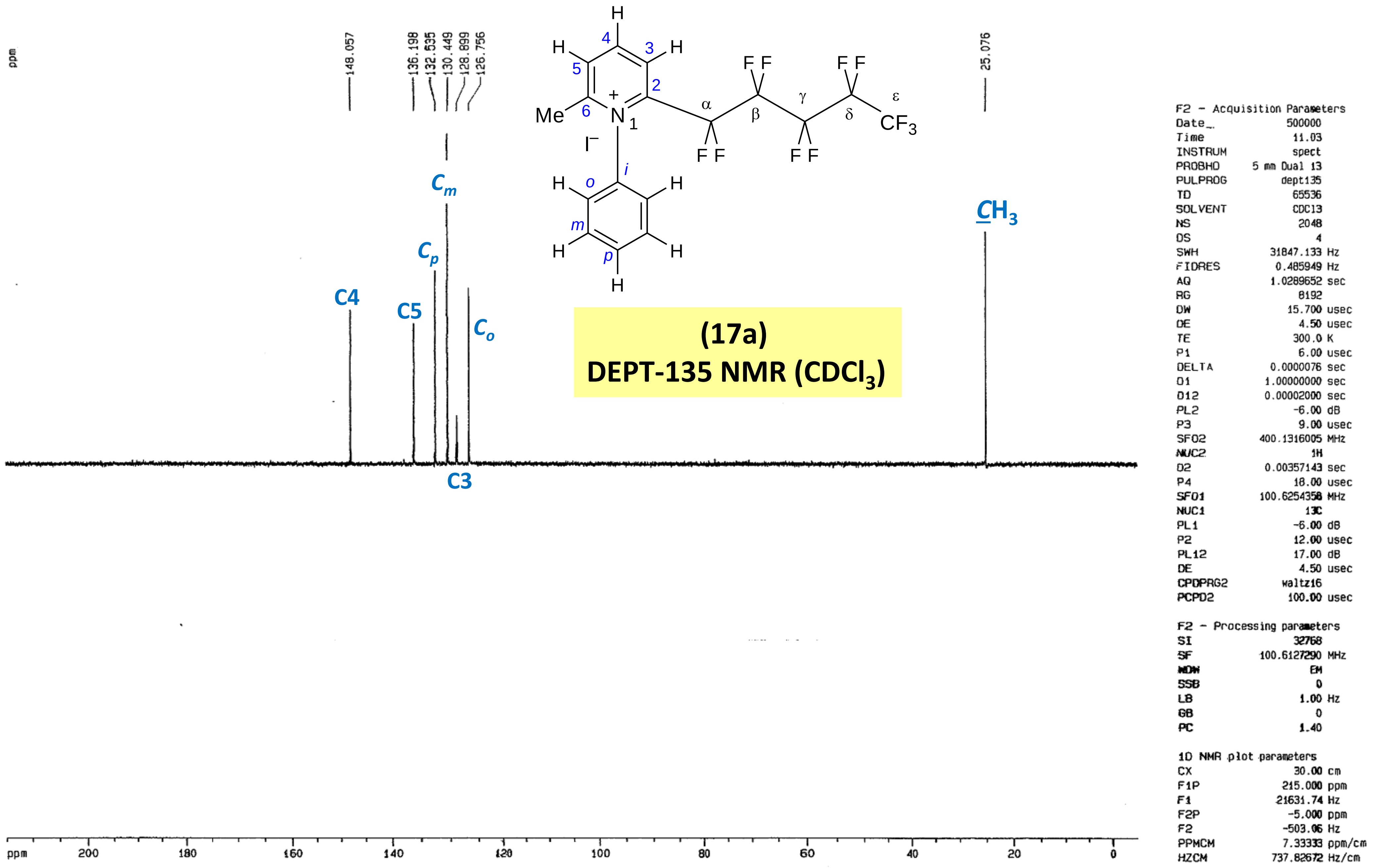
(17a)
DEPT-135 NMR ($CDCl_3$)
148.057
136.198
132.535
130.449
128.899
126.756
25.076
C4
C5
C_p
C_m
C_o
C3
$\underline{C}H_3$
Me
I^-
CF_3
F2 - Acquisition Parameters
Date_ 500000
Time 11.03
INSTRUM spect
PROBHD 5 mm Dual 13
PULPROG dept135
TD 65536
SOLVENT CDCl3
NS 2048
DS 4
SWH 31847.133 Hz
FIDRES 0.485949 Hz
AQ 1.0289652 sec
RG 8192
DW 15.700 usec
DE 4.50 usec
TE 300.0 K
P1 6.00 usec
DELTA 0.0000076 sec
D1 1.00000000 sec
D12 0.00002000 sec
PL2 -6.00 dB
P3 9.00 usec
SFO2 400.1316005 MHz
NUC2 1H
D2 0.00357143 sec
P4 18.00 usec
SFO1 100.6254358 MHz
NUC1 13C
PL1 -6.00 dB
P2 12.00 usec
PL12 17.00 dB
DE 4.50 usec
CPDPRG2 waltz16
PCPD2 100.00 usec
F2 - Processing parameters
SI 32768
SF 100.6127290 MHz
WDW EM
SSB 0
LB 1.00 Hz
GB 0
PC 1.40
1D NMR plot parameters
CX 30.00 cm
F1P 215.000 ppm
F1 21631.74 Hz
F2P -5.000 ppm
F2 -503.06 Hz
PPMCM 7.33333 ppm/cm
HZCM 737.82672 Hz/cm
ppm
200
180
160
140
120
100
80
60
40
20
0

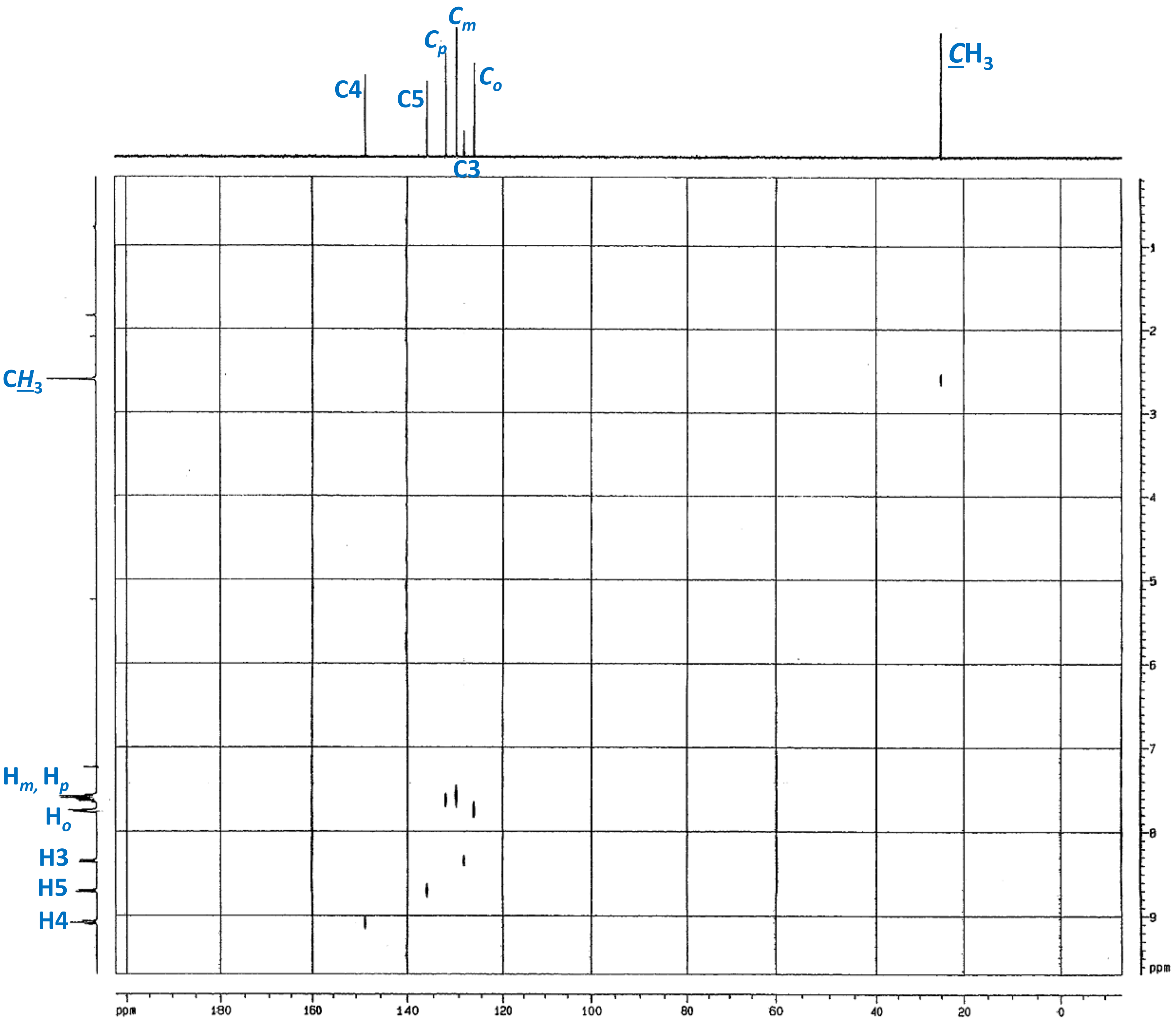

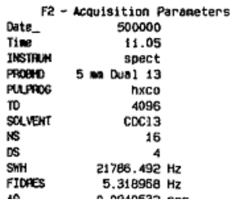

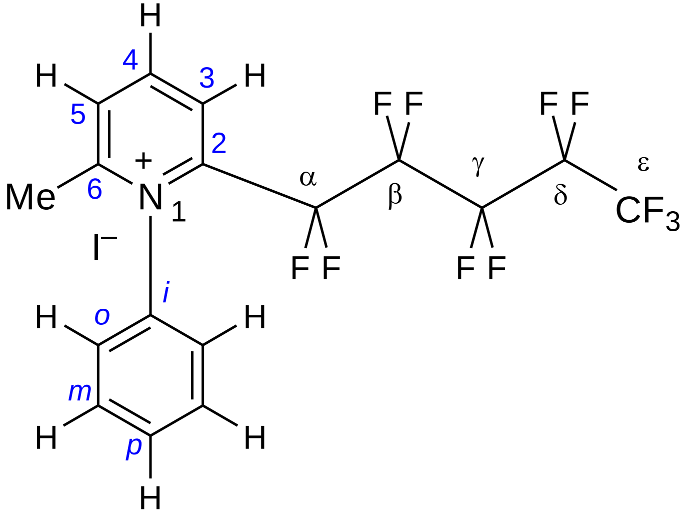

(17a)

HETCOR ^{1}H-^{13}C NMR

($CDCl_3$)

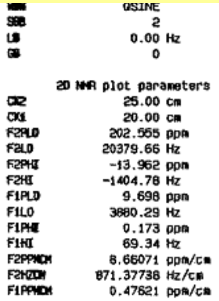

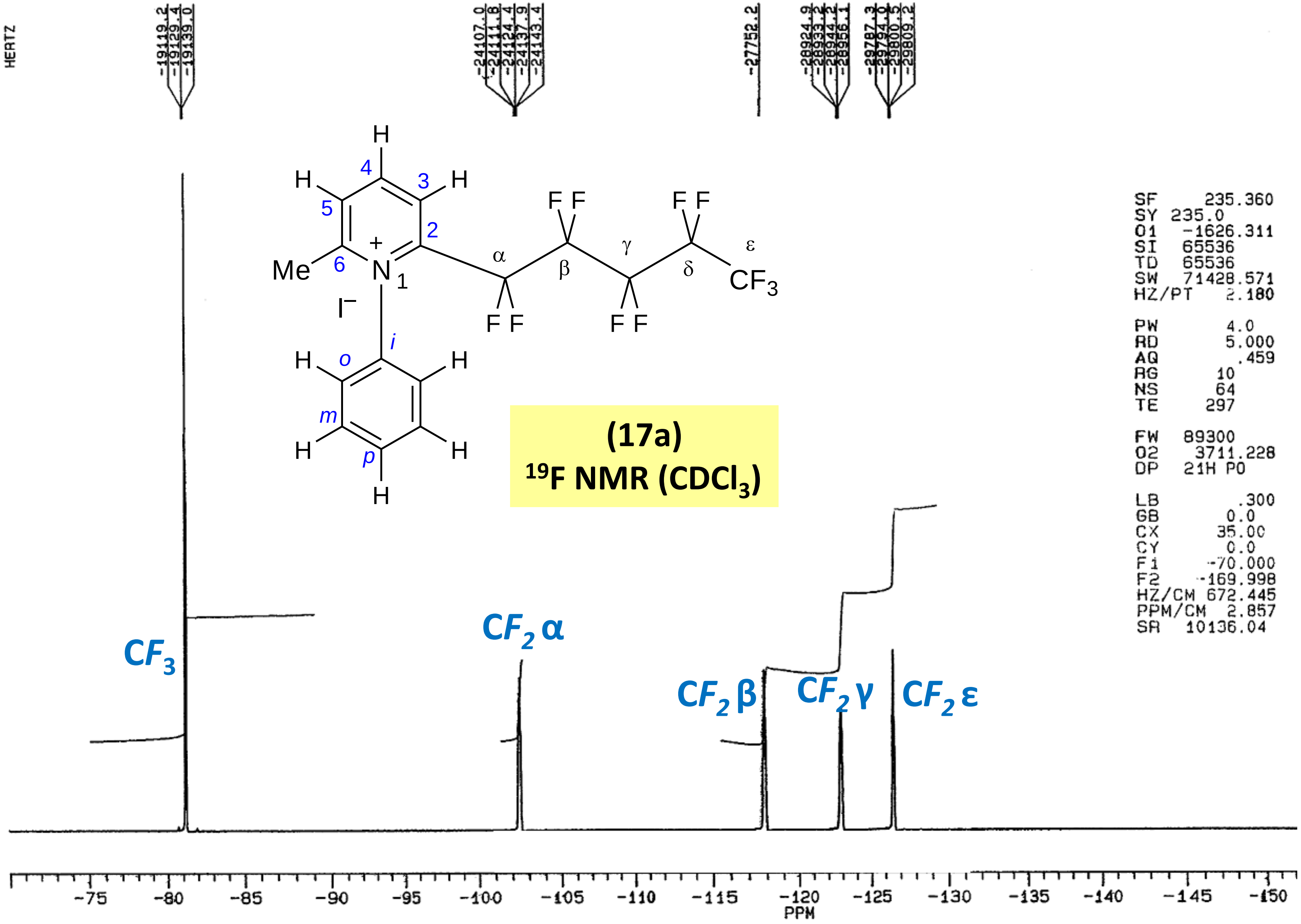

HERTZ
-19119.2
-19129.4
-19139.0
-27752.2
(17a)
19F NMR (CDCl3)
CF3
CF2 α
CF2 β
CF2 γ
CF2 ε
Me
N
I−
CF3
SF 235.360
SY 235.0
O1 -1626.311
SI 65536
TD 65536
SW 71428.571
HZ/PT 2.180
PW 4.0
RD 5.000
AQ .459
RG 10
NS 64
TE 297
FW 89300
O2 3711.228
DP 21H PO
LB .300
GB 0.0
CX 35.00
CY 0.0
F1 -70.000
F2 -169.998
HZ/CM 672.445
PPM/CM 2.857
SR 10136.04
PPM

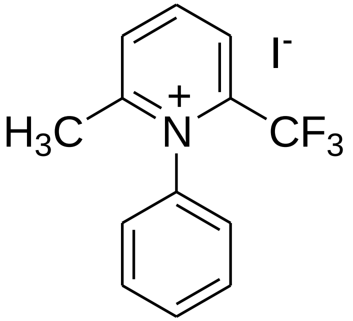

(17a')
^{1}H NMR ($CDCl_3$)

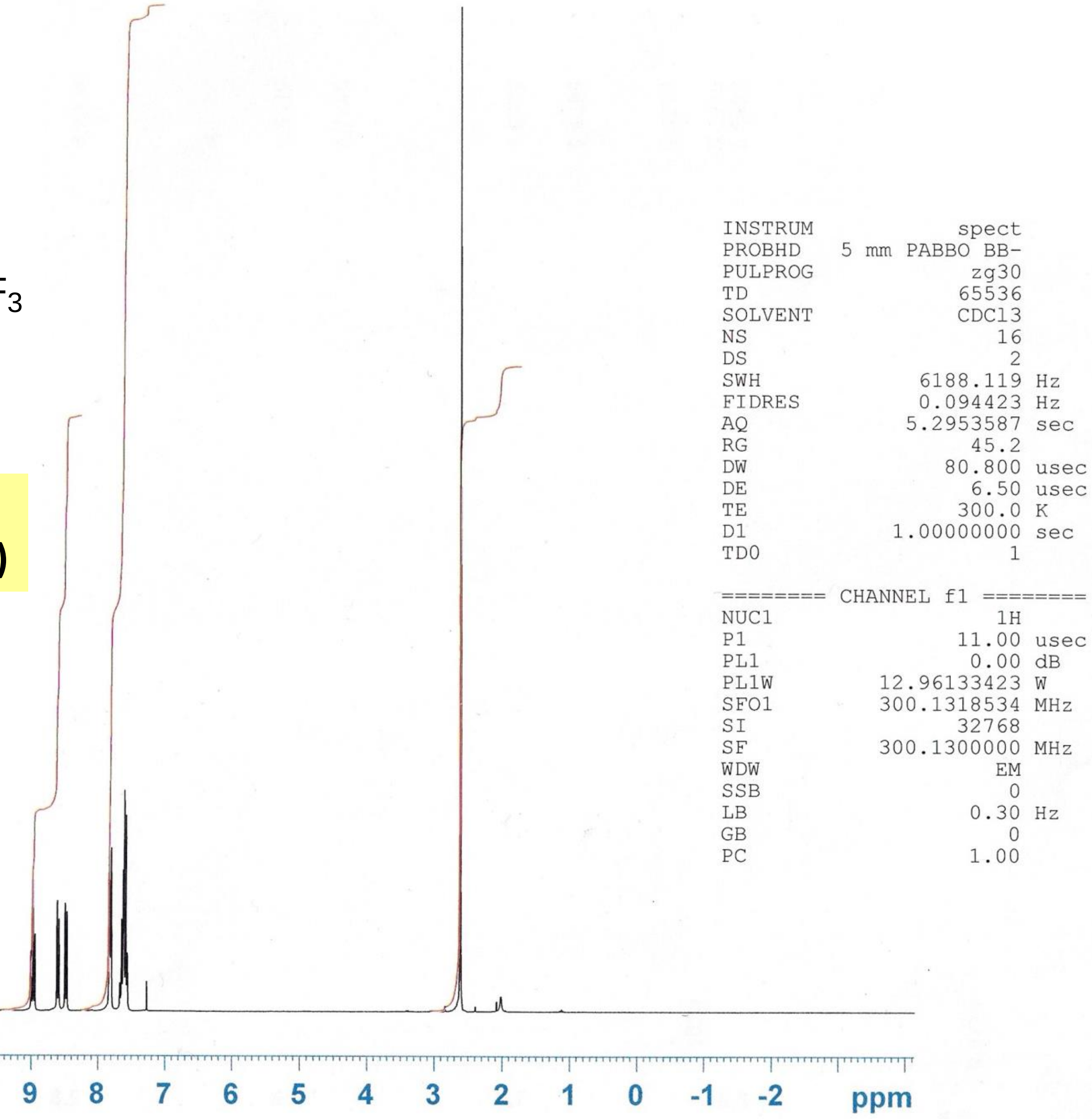

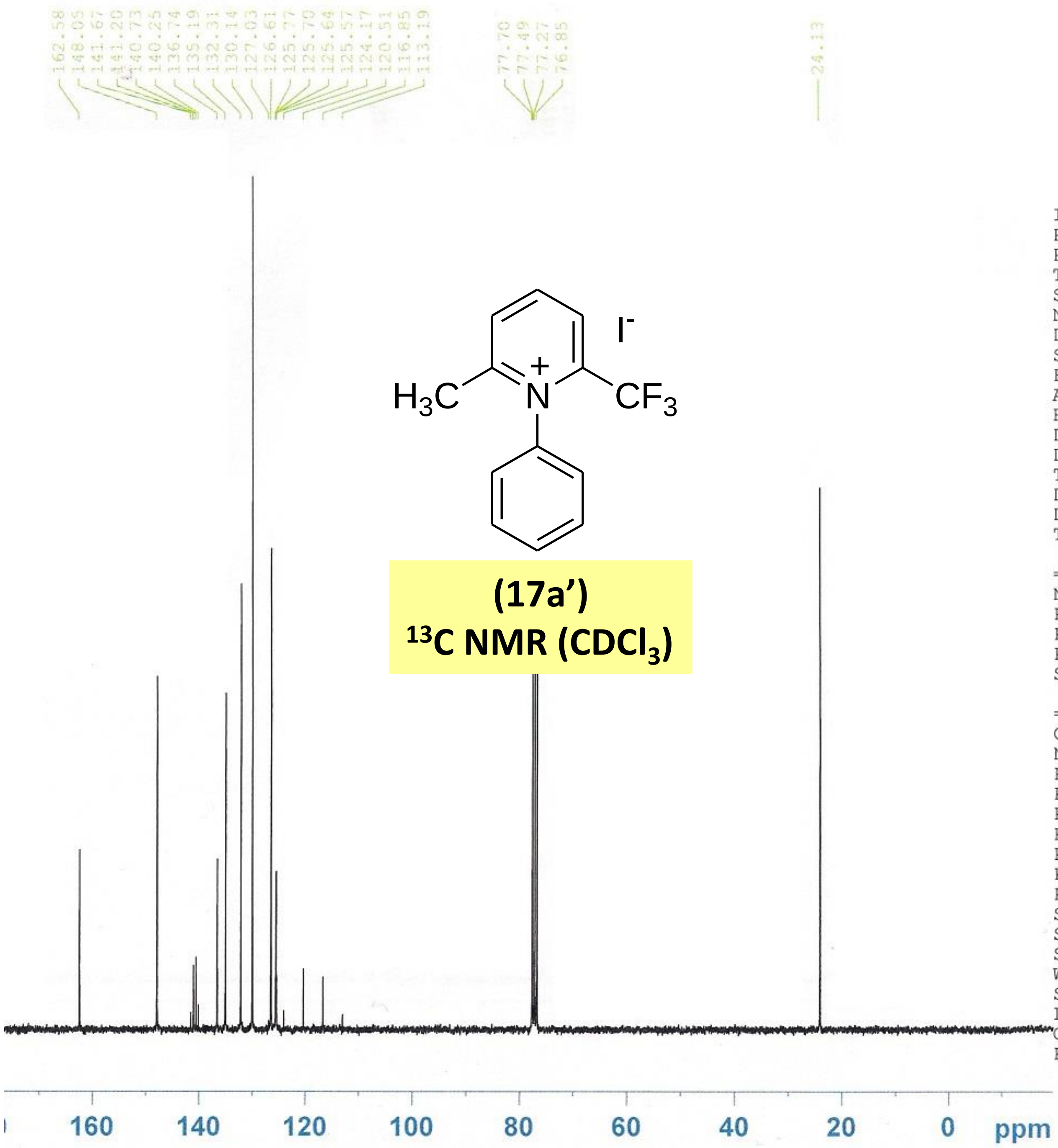

(17a')
^{13}C NMR ($CDCl_3$)

INSTRUM	spect
PROBHD	5 mm PABBO BB-
PULPROG	zgpg30
TD	65536
SOLVENT	CDCl3
NS	1024
DS	4
SWH	18028.846 Hz
FIDRES	0.275098 Hz
AQ	1.8175818 sec
RG	2050
DW	27.733 usec
DE	6.50 usec
TE	300.0 K
D1	2.00000000 sec
D11	0.03000000 sec
TD0	1
======== CHANNEL f1 ========	
NUC1	13C
P1	8.50 usec
PL1	-2.00 dB
PL1W	47.61361694 W
SFO1	75.4752953 MHz
======== CHANNEL f2 ========	
CPDPRG2	waltz16
NUC2	1H
PCPD2	100.00 usec
PL2	0.00 dB
PL12	20.00 dB
PL13	20.00 dB
PL2W	12.96133423 W
PL12W	0.12961334 W
PL13W	0.12961334 W
SFO2	300.1312005 MHz
SI	32768
SF	75.4677490 MHz
WDW	EM
SSB	0
LB	1.00 Hz
GB	0
PC	1.40

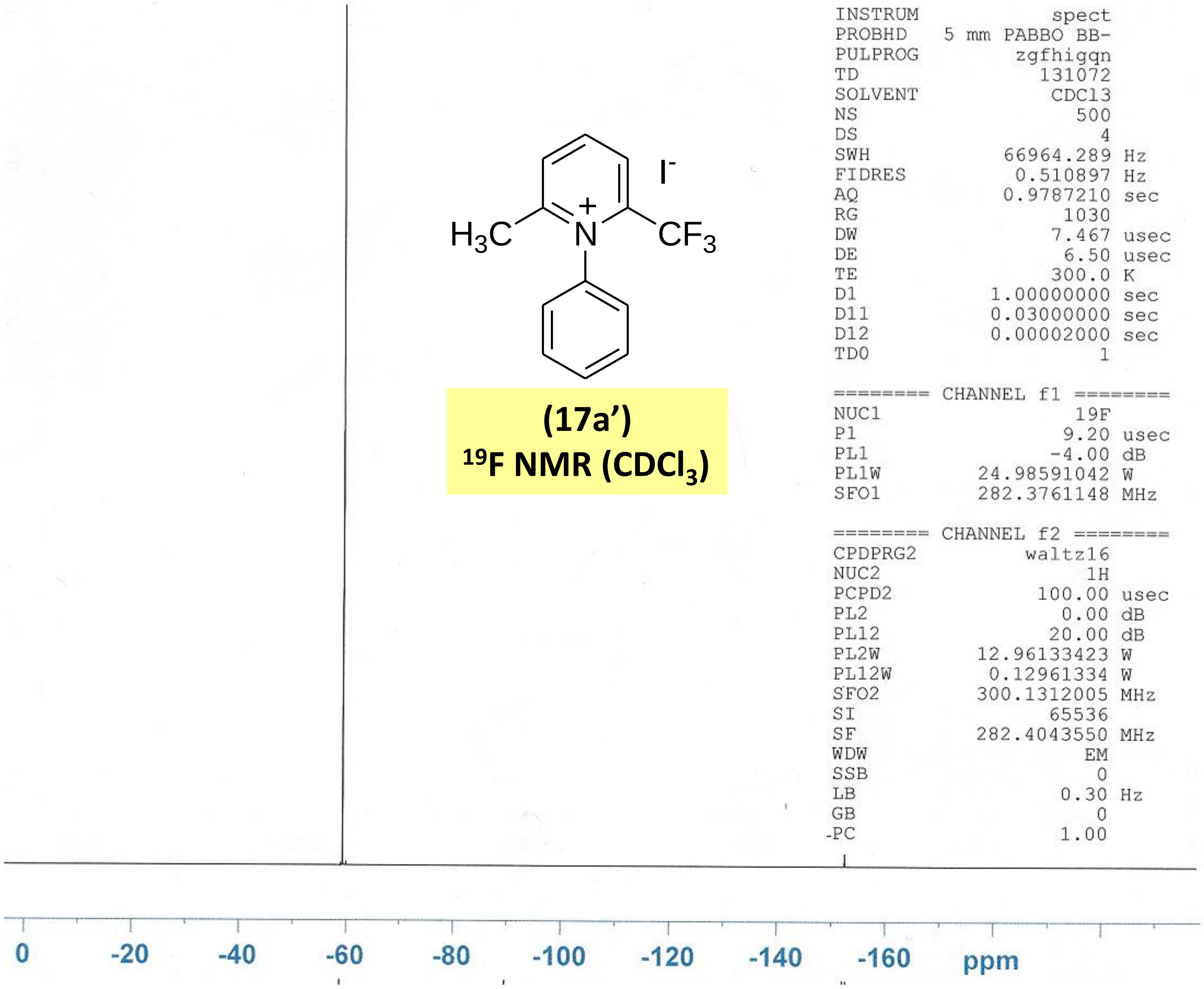

I-
H3C
N
+
CF3
(17a')
19F NMR (CDCl3)
INSTRUM spect
PROBHD 5 mm PABBO BB-
PULPROG zgfhigqn
TD 131072
SOLVENT CDCl3
NS 500
DS 4
SWH 66964.289 Hz
FIDRES 0.510897 Hz
AQ 0.9787210 sec
RG 1030
DW 7.467 usec
DE 6.50 usec
TE 300.0 K
D1 1.00000000 sec
D11 0.03000000 sec
D12 0.00002000 sec
TD0 1
======== CHANNEL f1 ========
NUC1 19F
P1 9.20 usec
PL1 -4.00 dB
PL1W 24.98591042 W
SFO1 282.3761148 MHz
======== CHANNEL f2 ========
CPDPRG2 waltz16
NUC2 1H
PCPD2 100.00 usec
PL2 0.00 dB
PL12 20.00 dB
PL2W 12.96133423 W
PL12W 0.12961334 W
SFO2 300.1312005 MHz
SI 65536
SF 282.4043550 MHz
WDW EM
SSB 0
LB 0.30 Hz
GB 0
PC 1.00
0
-20
-40
-60
-80
-100
-120
-140
-160
ppm

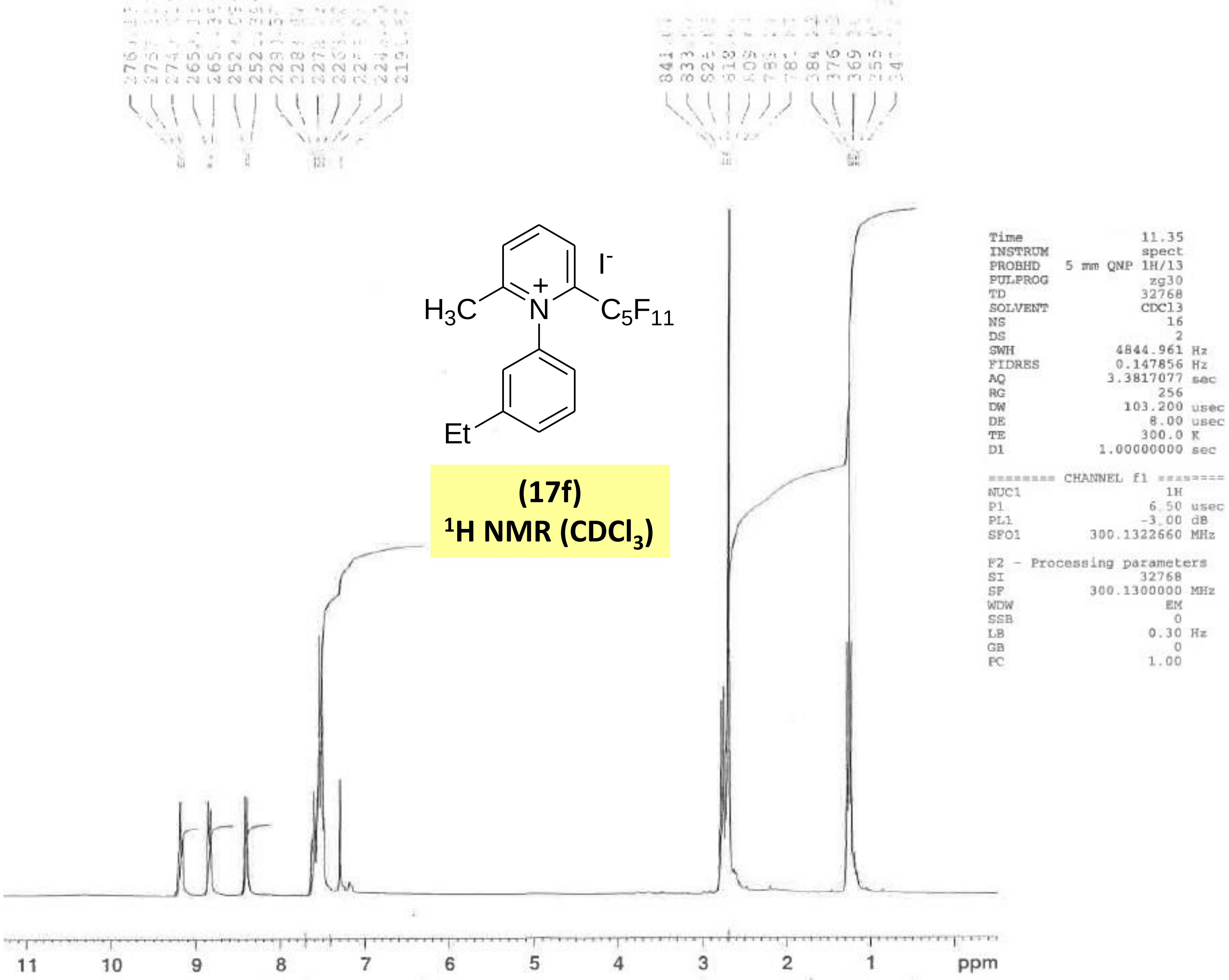

(17f)
^1H NMR ($CDCl_3$)

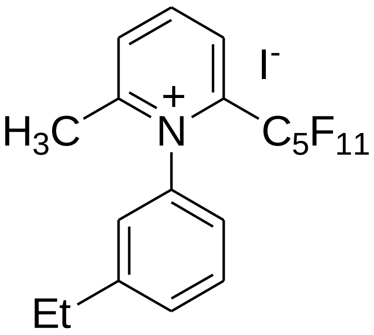

(17f)

^{13}C NMR ($CDCl_3$)

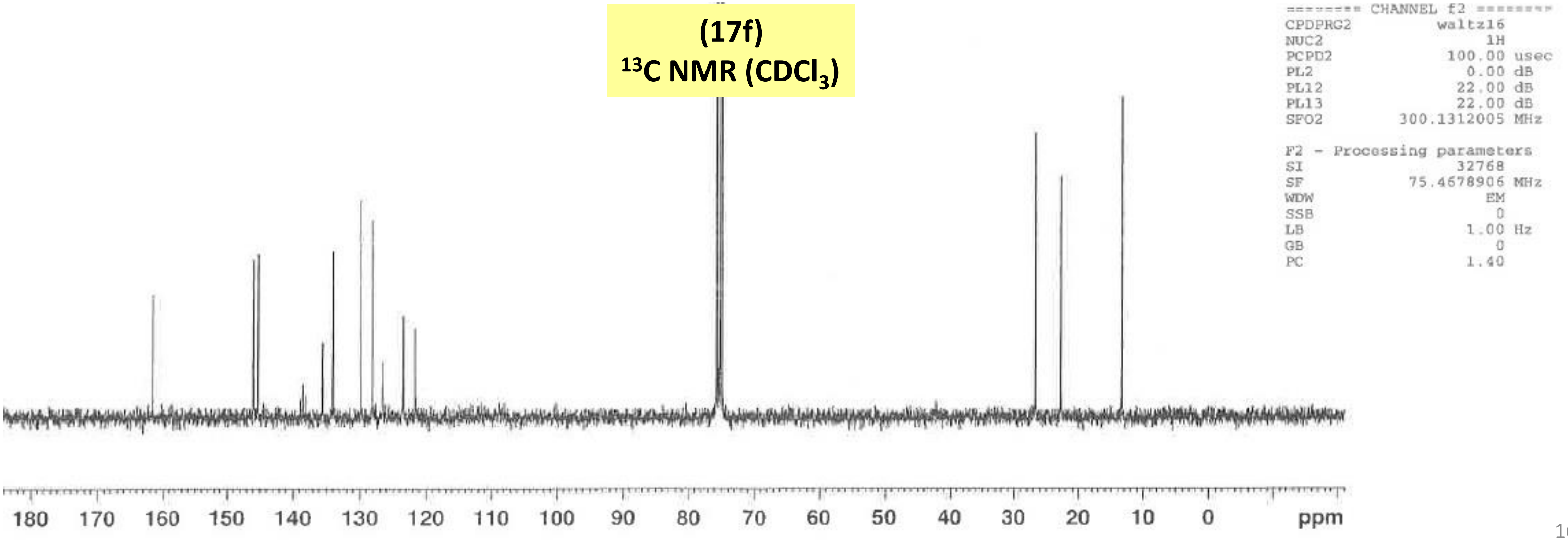

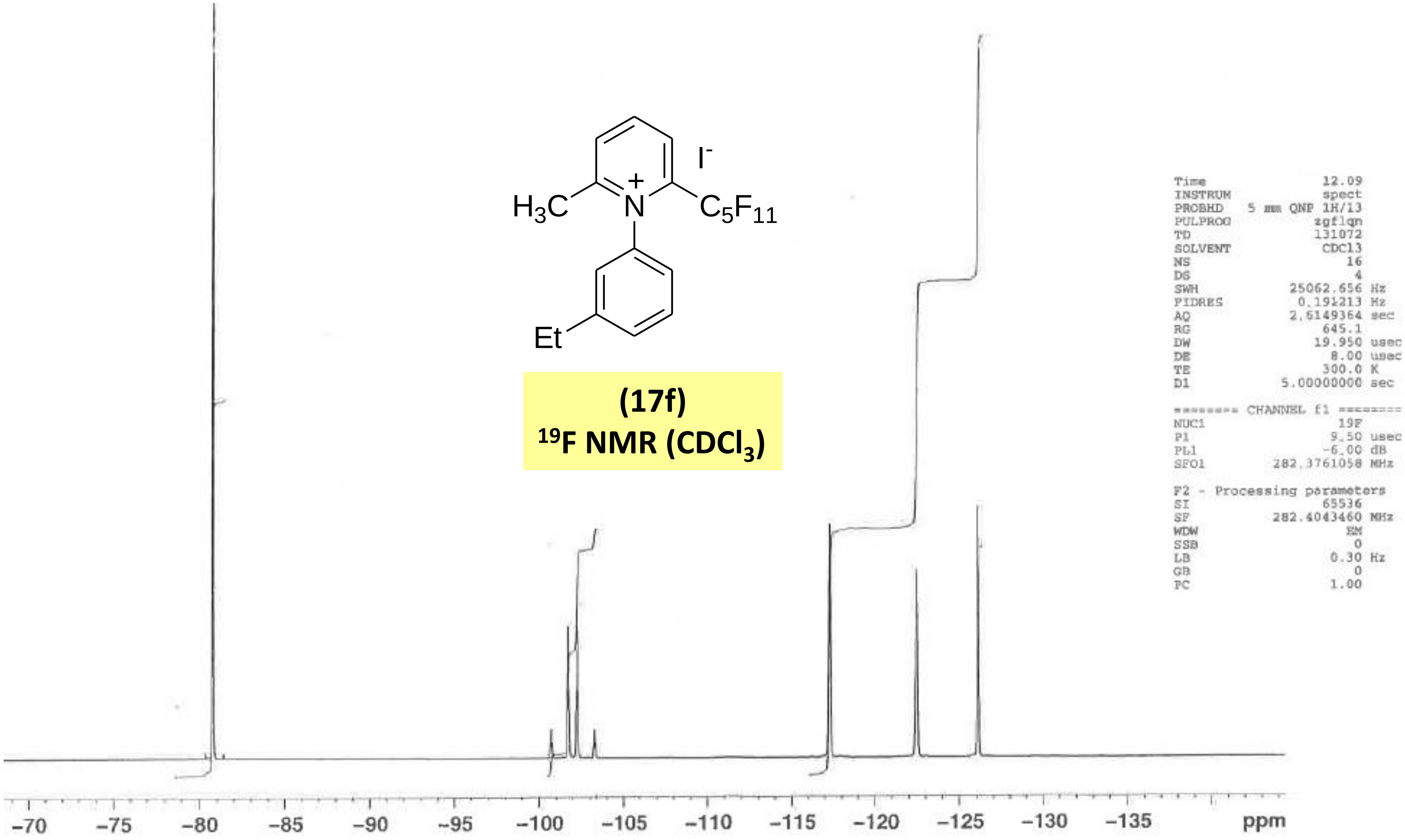

(17f)
^{19}F NMR ($CDCl_3$)

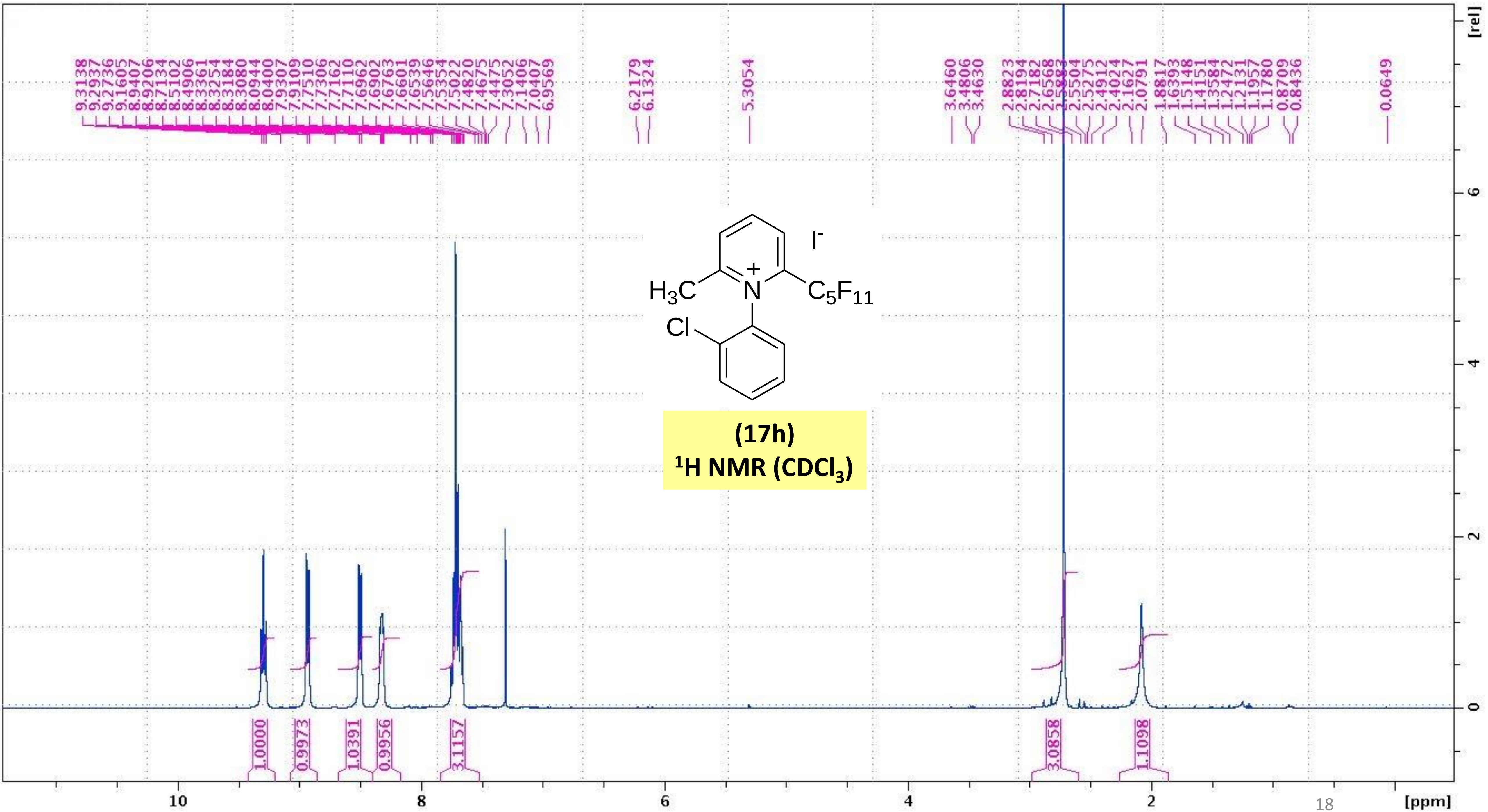
9.3138
9.2937
9.2736
9.1605
8.9407
8.9206
8.7134
8.5102
8.4906
8.3361
8.3254
8.3184
8.3080
8.0944
8.0400
7.9307
7.9109
7.7510
7.7306
7.7162
7.7110
7.6962
7.6902
7.6763
7.6601
7.6539
7.5646
7.5354
7.5022
7.4820
7.4675
7.4475
7.3052
7.1406
7.0407
6.9569
6.2179
6.1324
5.3054
3.6460
3.4806
3.4630
2.8823
2.8194
2.7182
2.6568
2.5883
2.5504
2.5275
2.4912
2.4024
2.1627
2.0791
1.8817
1.6393
1.5148
1.4151
1.3584
1.2472
1.2131
1.1957
1.1780
0.8709
0.8436
0.0649
I-
H3C
N
+
C5F11
Cl
(17h)
1H NMR (CDCl3)
1.0000
0.9973
1.0391
0.9956
3.1157
3.0858
1.1098
10
8
6
4
2
[ppm]
0
2
4
6
[rel]

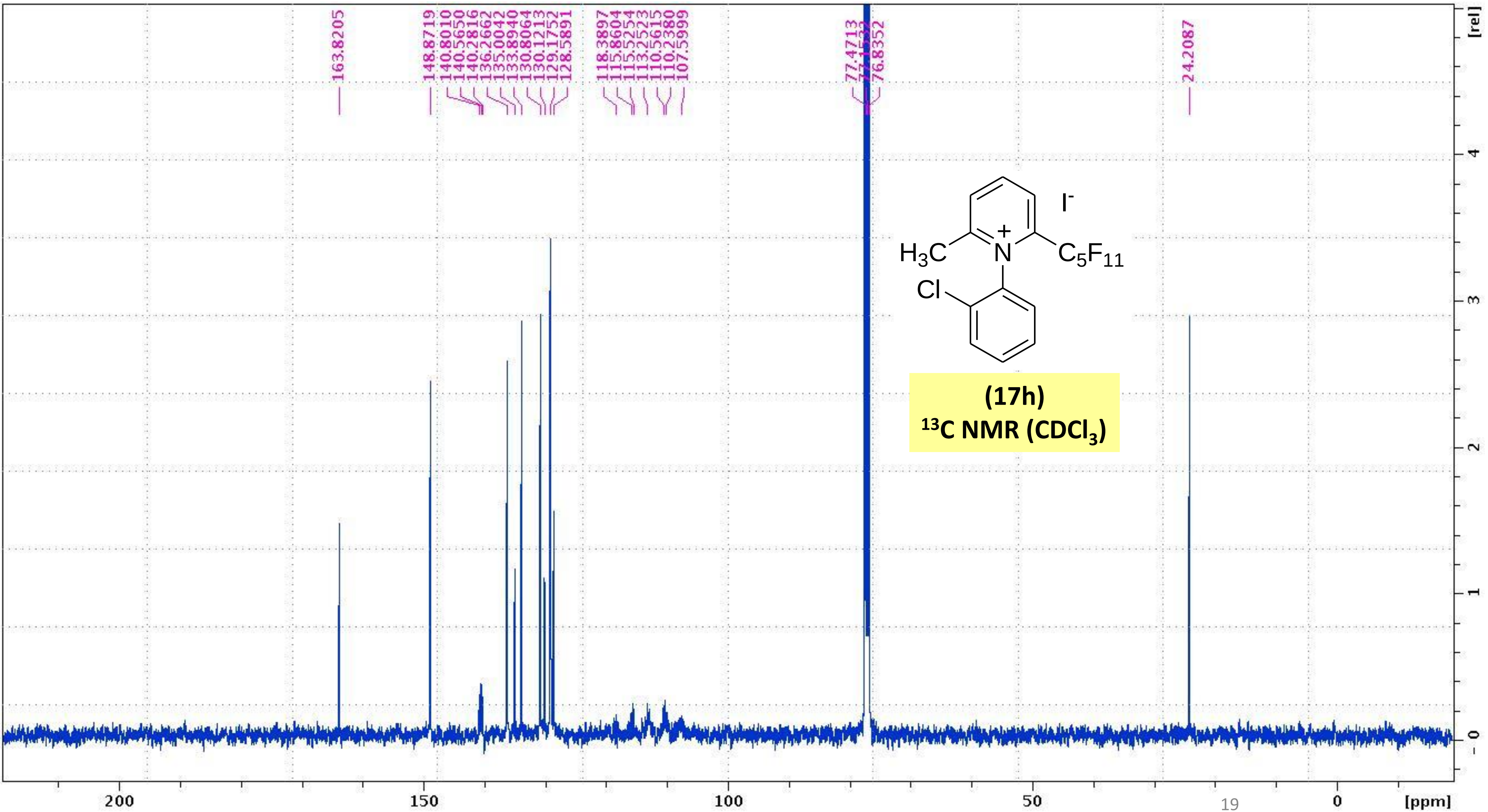

(17h)
^{13}C NMR ($CDCl_3$)

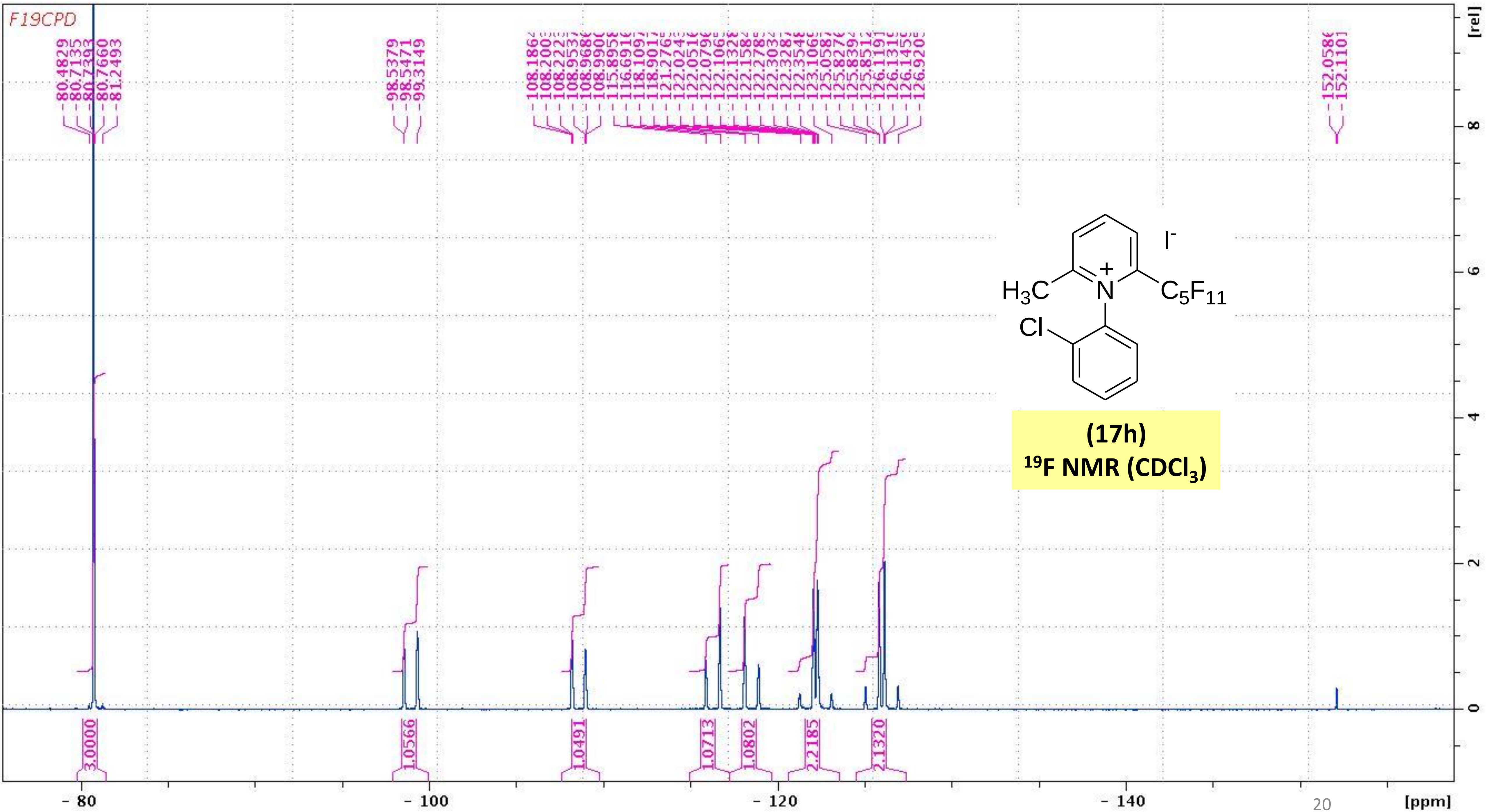
F19CPD
-80.4829
-80.7135
-80.7393
-80.7660
-81.2493
-98.5379
-98.5471
-99.3149
-152.0586
-152.1101
3.0000
1.0566
1.0491
1.0713
1.0802
2.2185
2.1320
I-
H3C
N+
C5F11
Cl
(17h)
19F NMR (CDCl3)
-80
-100
-120
-140
[ppm]
[rel]

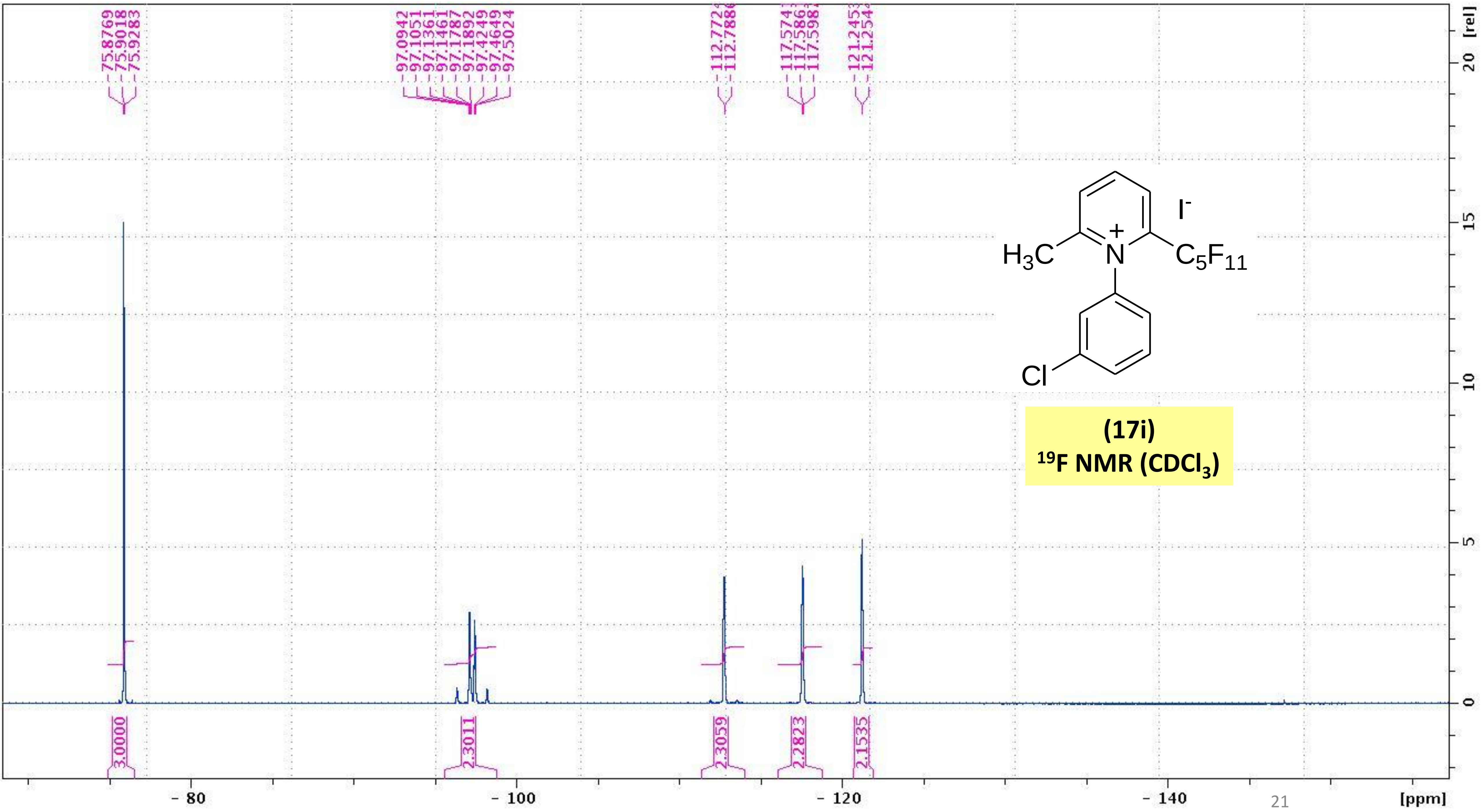

-75.8769
-75.9018
-75.9283
-97.0942
-97.1051
-97.1361
-97.1461
-97.1787
-97.1892
-97.4249
-97.4649
-97.5024
-112.7724
-112.7886
-117.5743
-117.5863
-117.5982
-121.2453
-121.2544
I-
H3C
N
+
C5F11
Cl
(17i)
19F NMR (CDCl3)
3.0000
2.3011
2.3059
2.2823
2.1535
- 80
- 100
- 120
- 140
[ppm]
[rel]

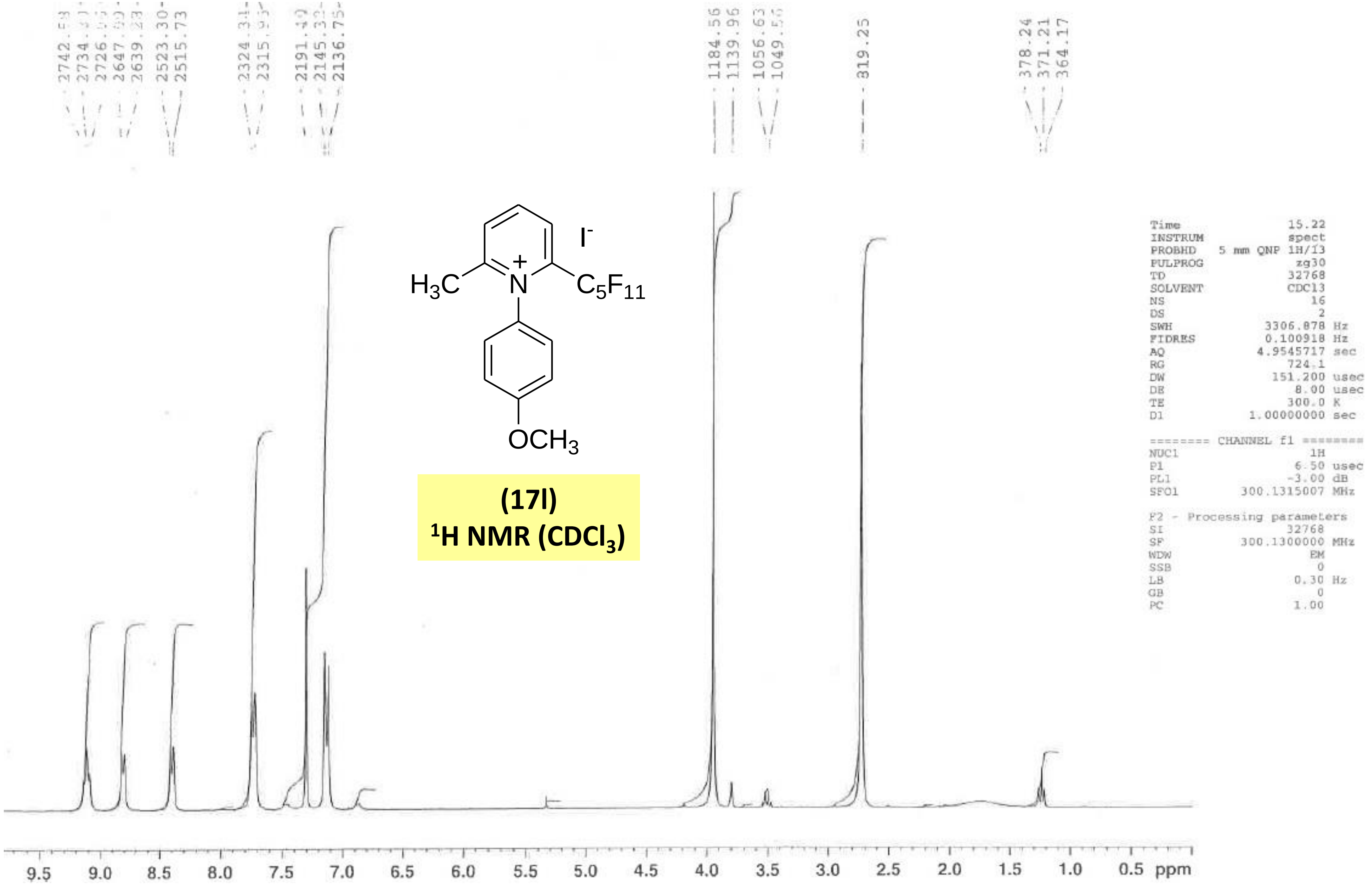

(17l)
^{1}H NMR ($CDCl_3$)

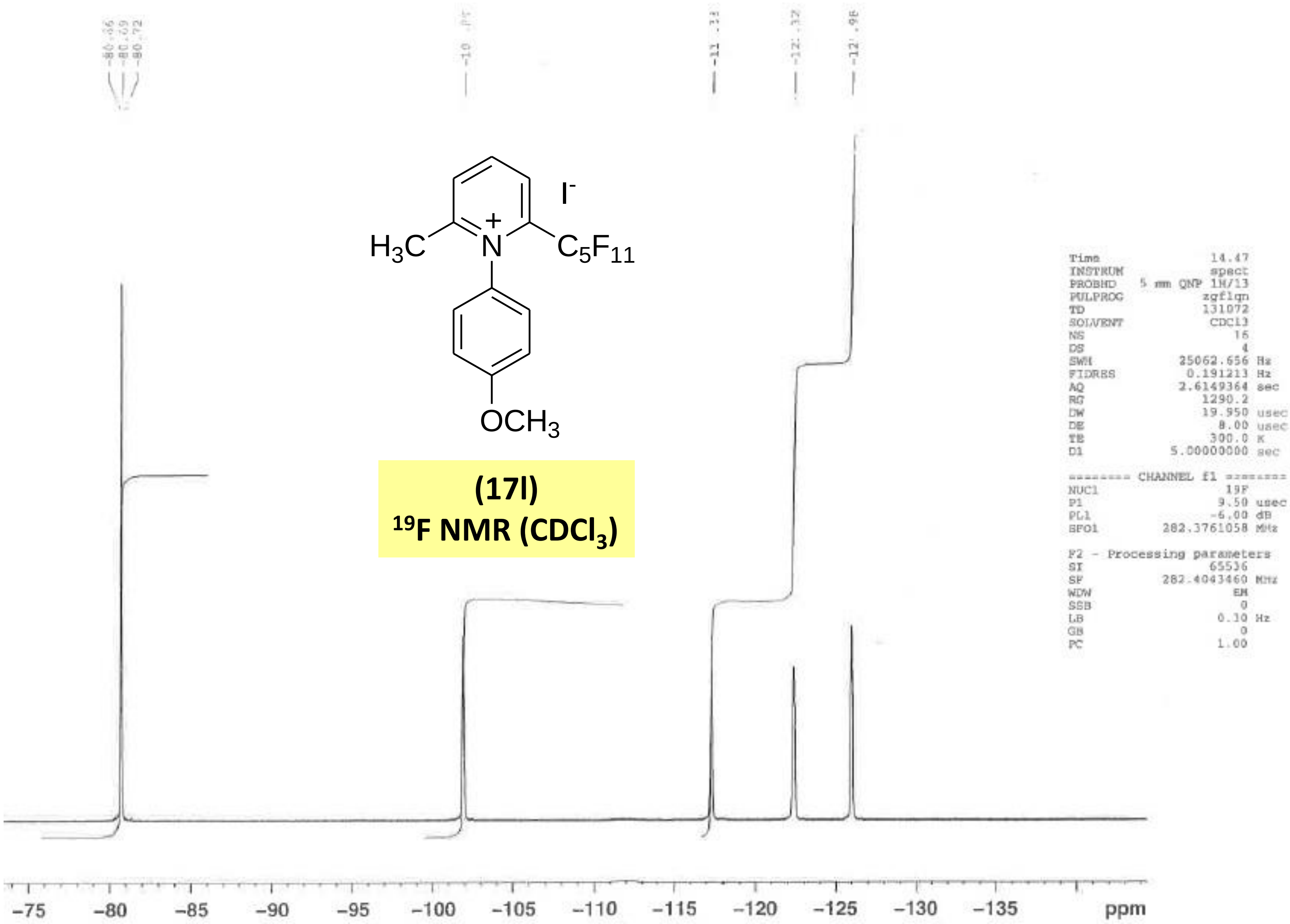

(17l)

^{19}F NMR ($CDCl_3$)

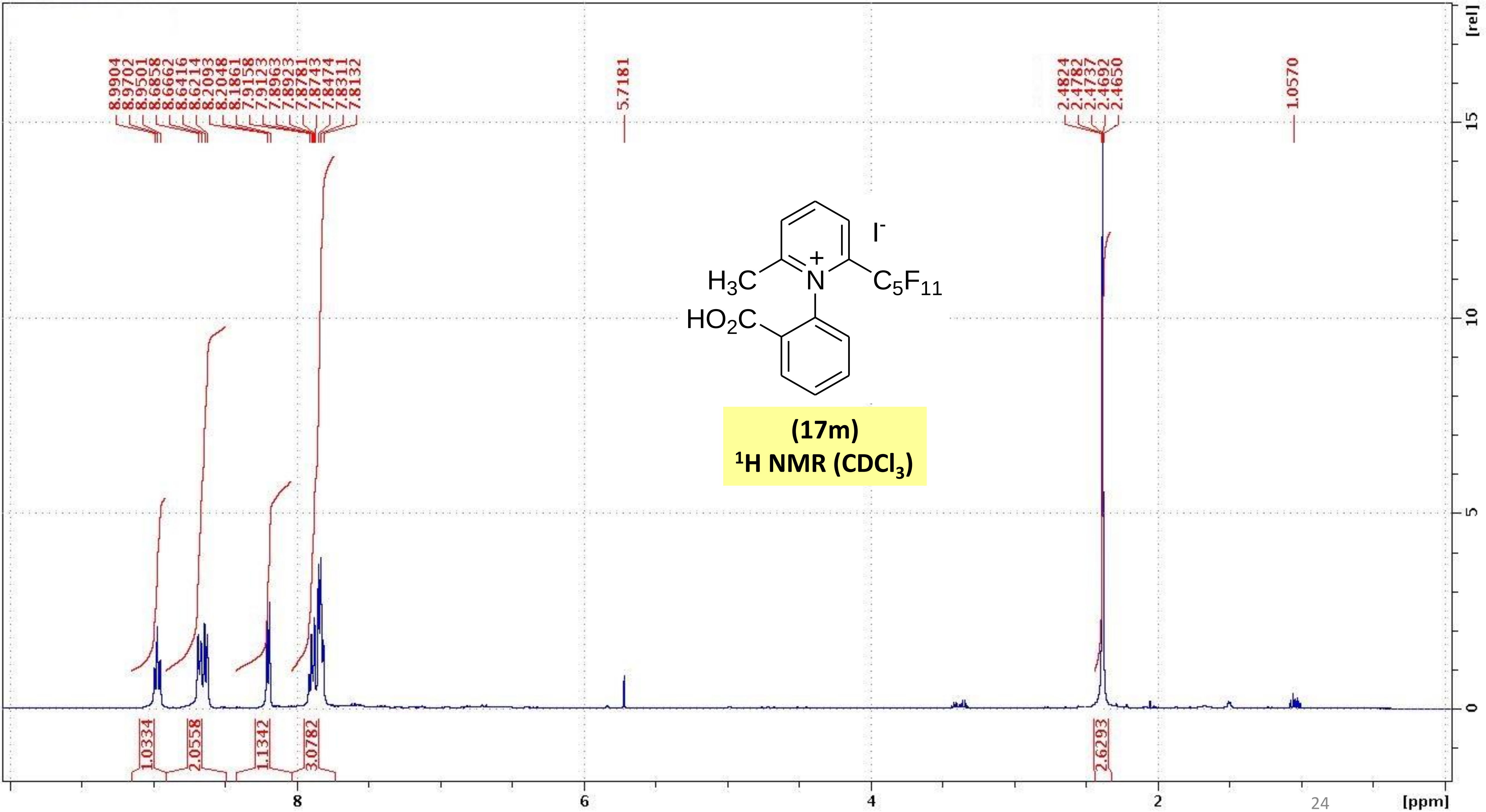
8.9904
8.9702
8.9501
8.6858
8.6662
8.6416
8.6214
8.2093
8.2048
8.1861
7.9158
7.9123
7.8963
7.8923
7.8781
7.8743
7.8474
7.8311
7.8132
5.7181
2.4824
2.4782
2.4737
2.4692
2.4650
1.0570
I-
H3C
N+
C5F11
HO2C
(17m)
1H NMR (CDCl3)
1.0334
2.0558
1.1342
3.0782
2.6293
8
6
4
2
[ppm]
0
5
10
15
[rel]

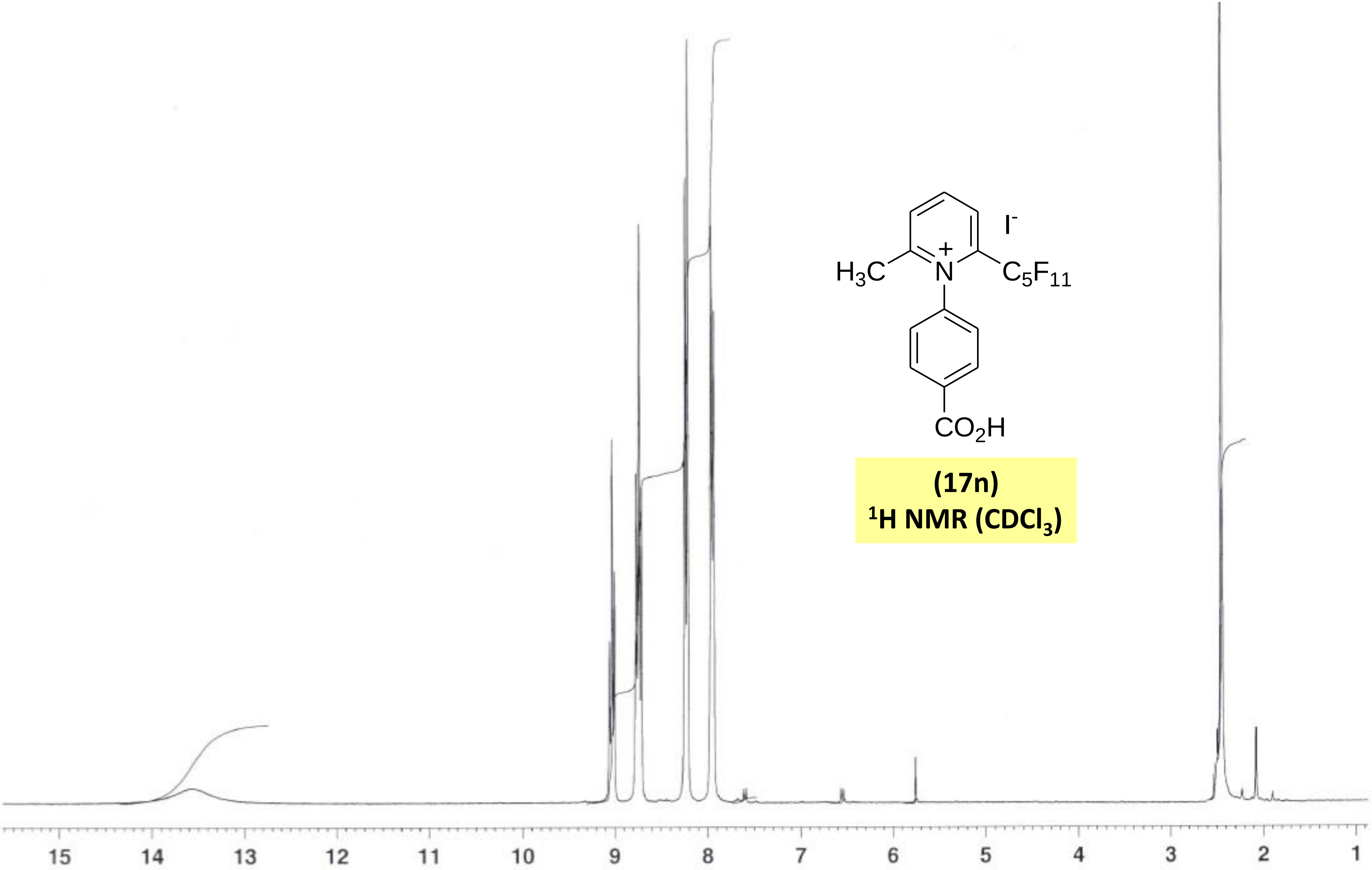

(17n)
^{1}H NMR ($CDCl_3$)

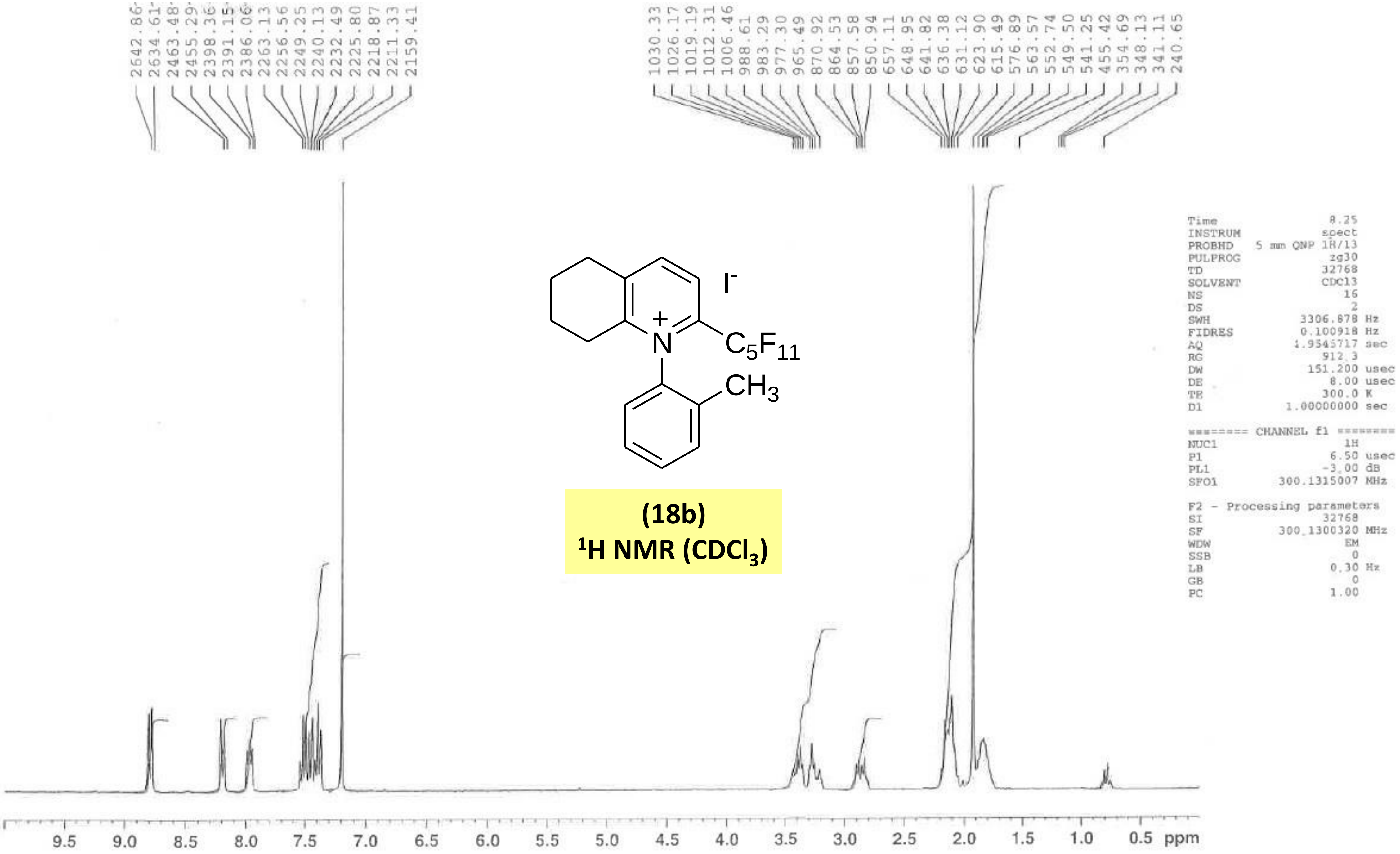

(18b)
^{1}H NMR ($CDCl_3$)

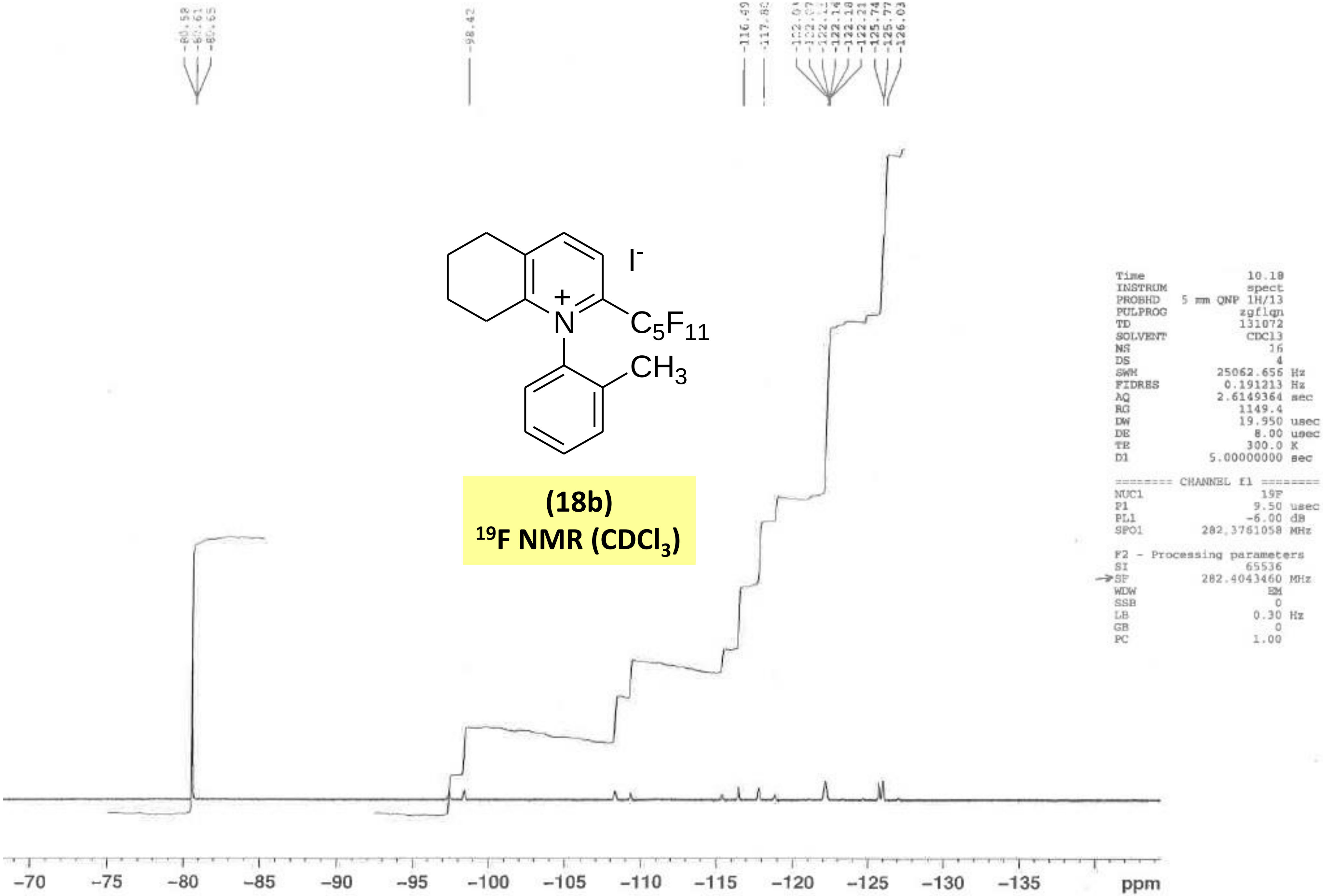

(18b)

^{19}F NMR ($CDCl_3$)

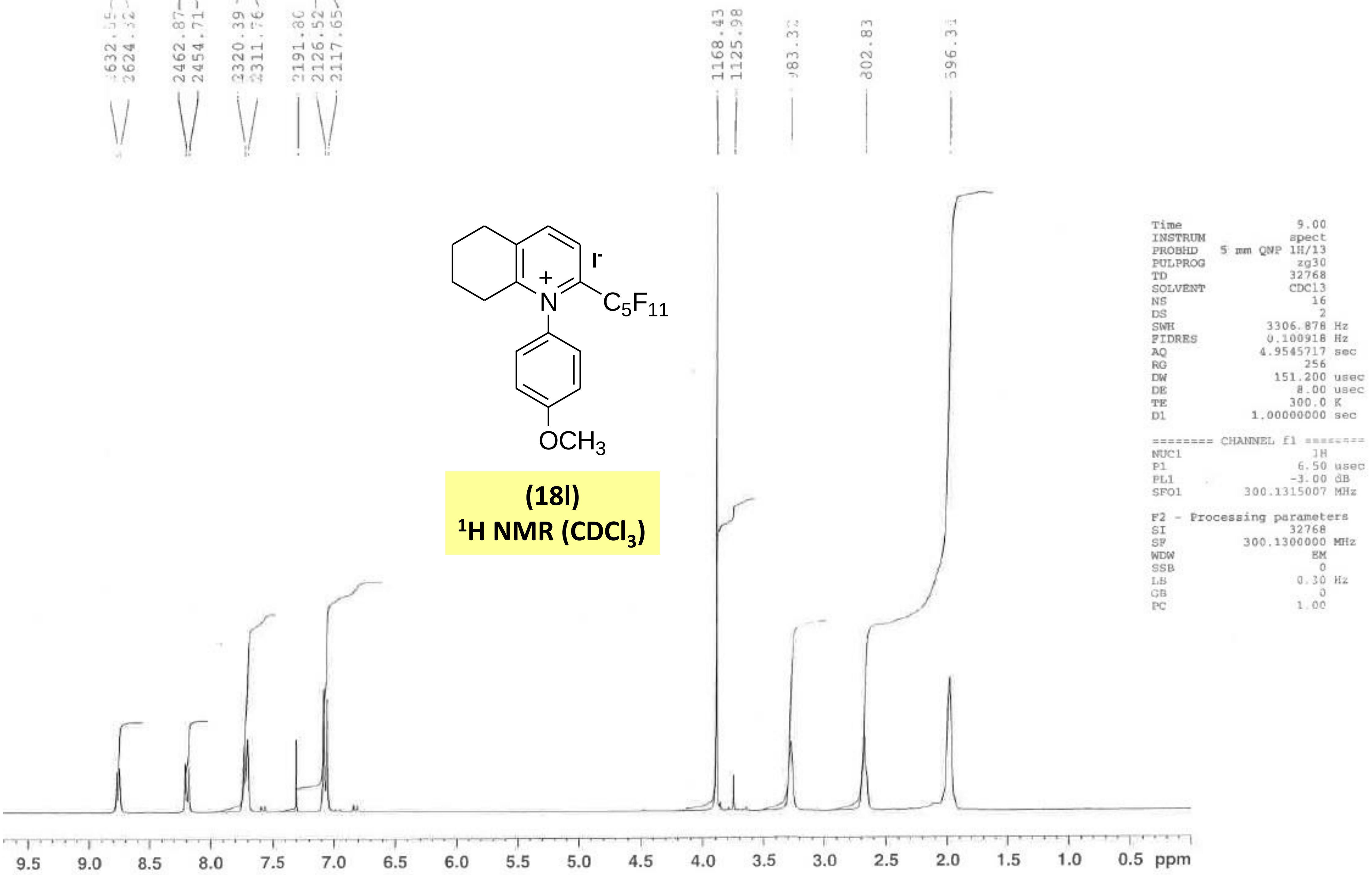

(18l)
^{1}H NMR ($CDCl_3$)

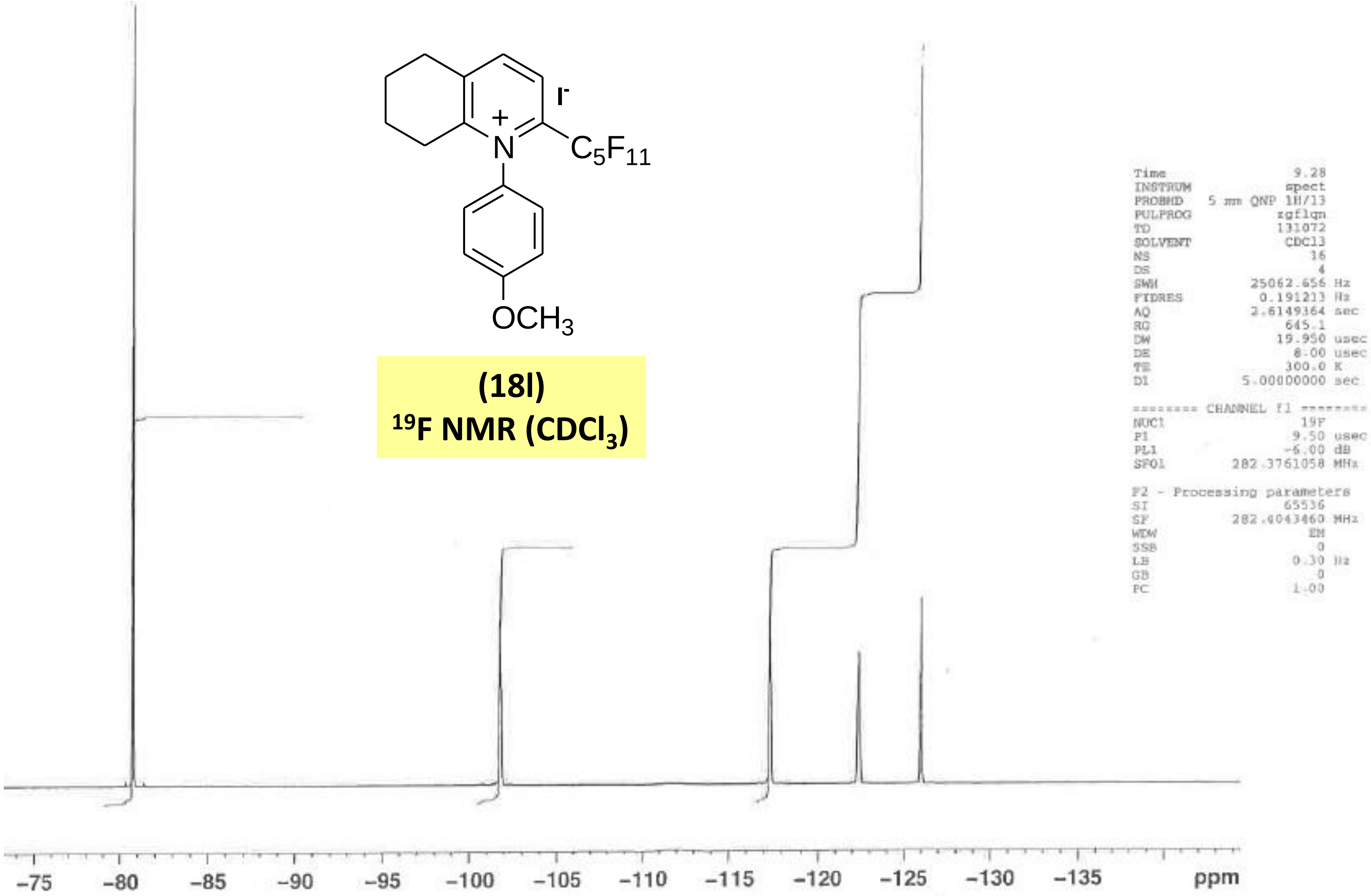
I-
N
C5F11
OCH3
(18l)
19F NMR (CDCl3)
Time 9.28
INSTRUM spect
PROBHD 5 mm QNP 1H/13
PULPROG zgflqn
TD 131072
SOLVENT CDCl3
NS 16
DS 4
SWH 25062.656 Hz
FIDRES 0.191213 Hz
AQ 2.6149364 sec
RG 645.1
DW 19.950 usec
DE 8.00 usec
TE 300.0 K
D1 5.00000000 sec
======== CHANNEL f1 ========
NUC1 19F
P1 9.50 usec
PL1 -6.00 dB
SFO1 282.3761058 MHz
F2 - Processing parameters
SI 65536
SF 282.4043460 MHz
WDW EM
SSB 0
LB 0.30 Hz
GB 0
PC 1.00
-75
-80
-85
-90
-95
-100
-105
-110
-115
-120
-125
-130
-135
ppm

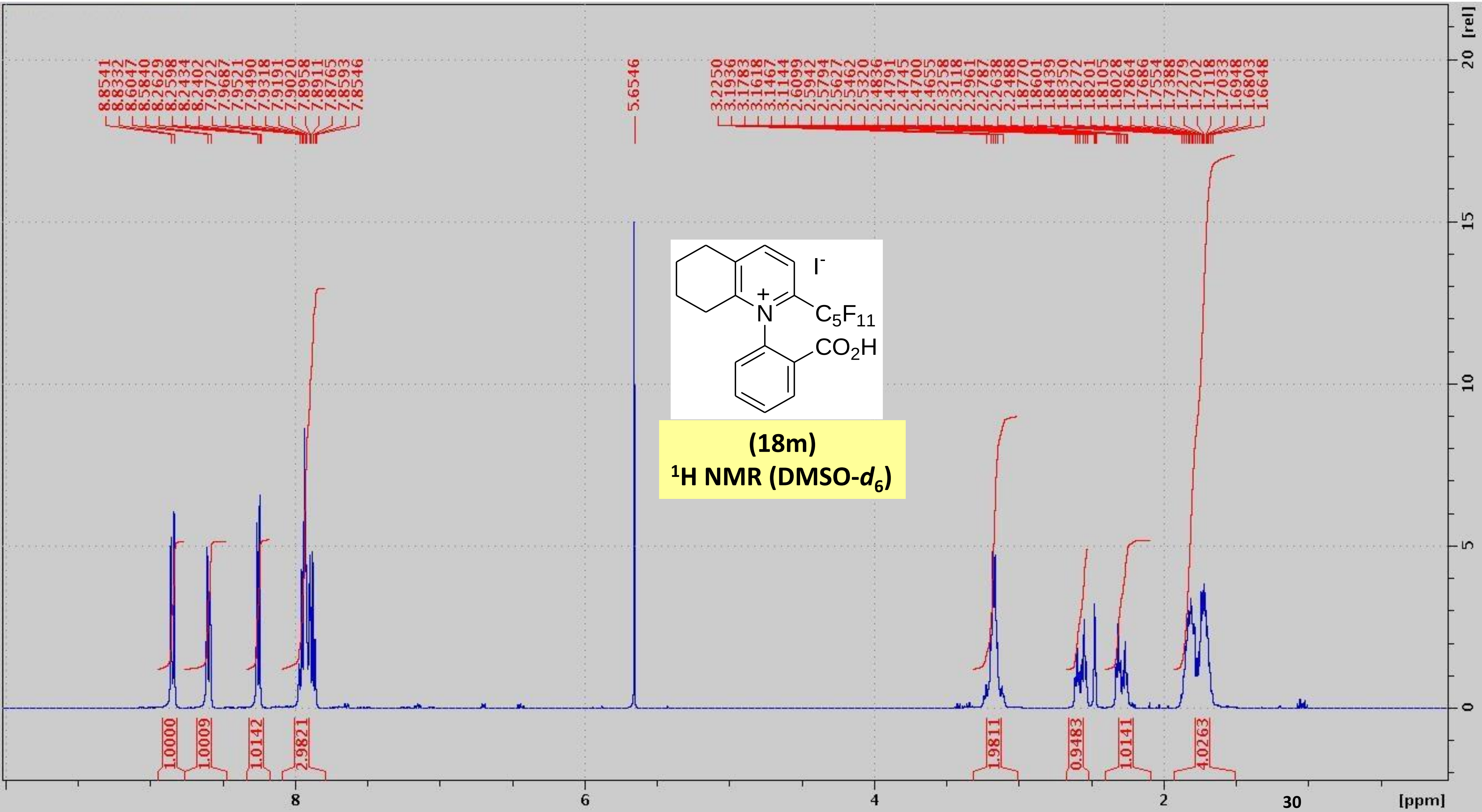
I-
N+
C5F11
CO2H
(18m)
1H NMR (DMSO-d6)
[ppm]
[rel]

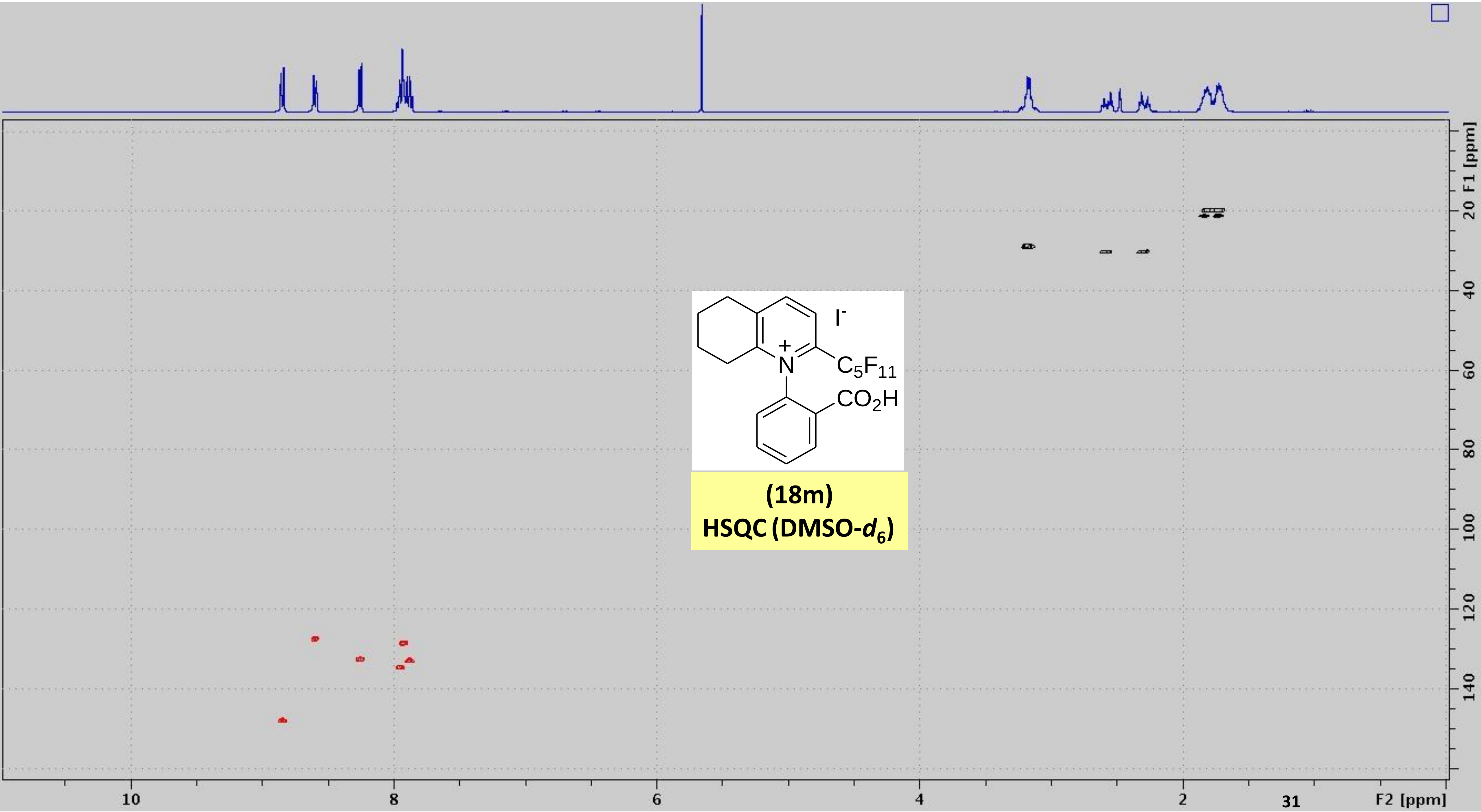

(18m)
HSQC (DMSO-d_6)

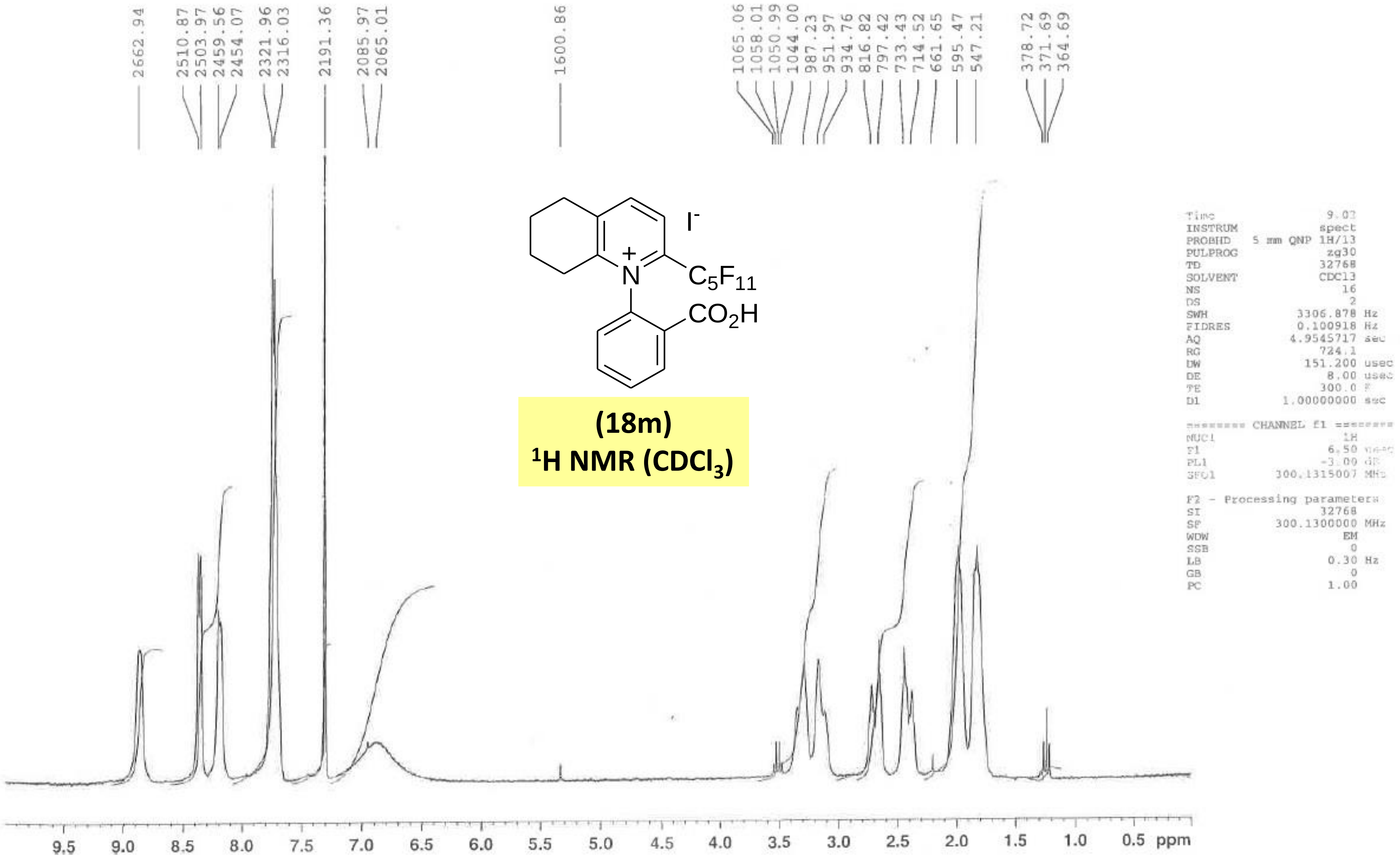

(18m)
^{1}H NMR ($CDCl_3$)

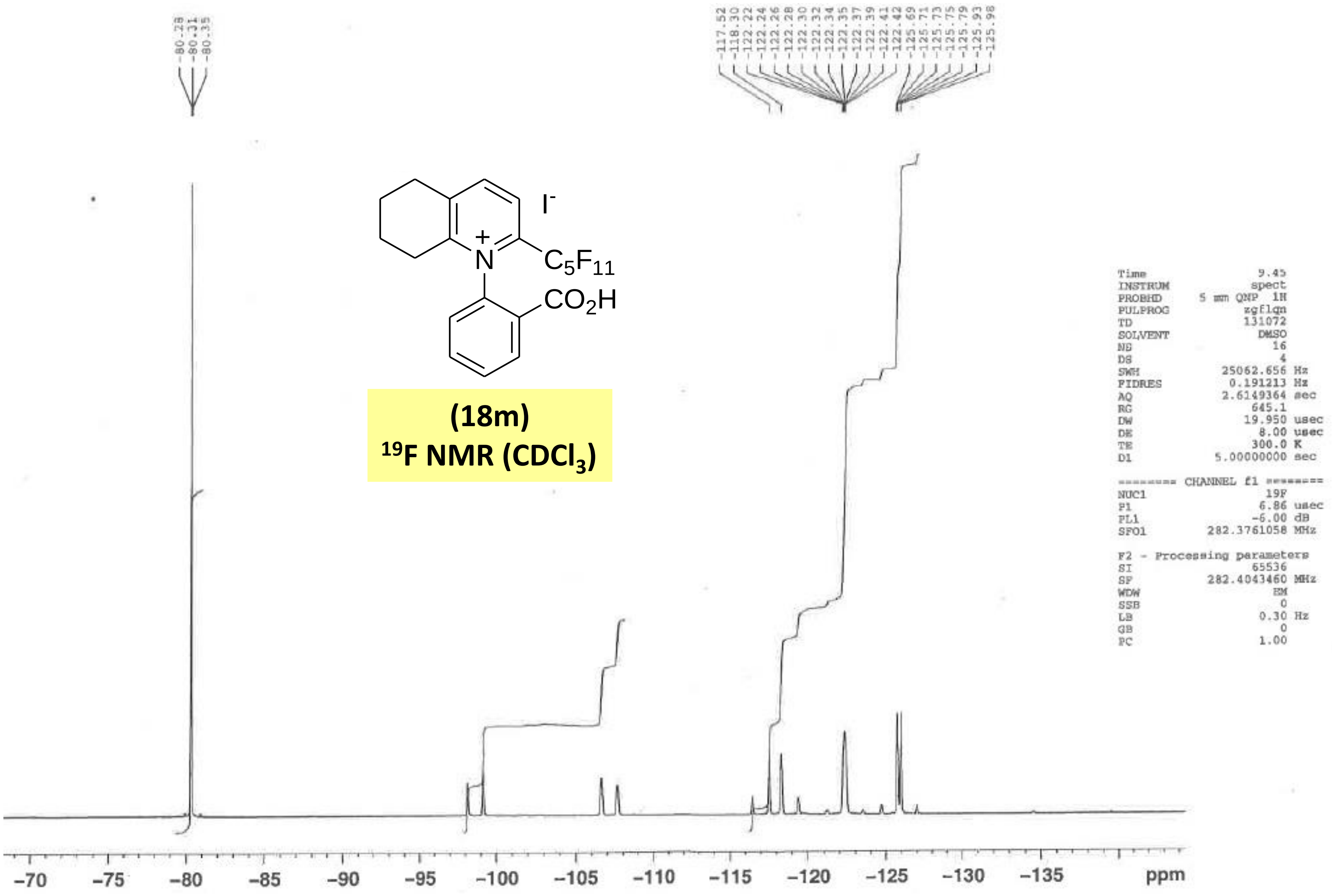

(18m)
^{19}F NMR ($CDCl_3$)

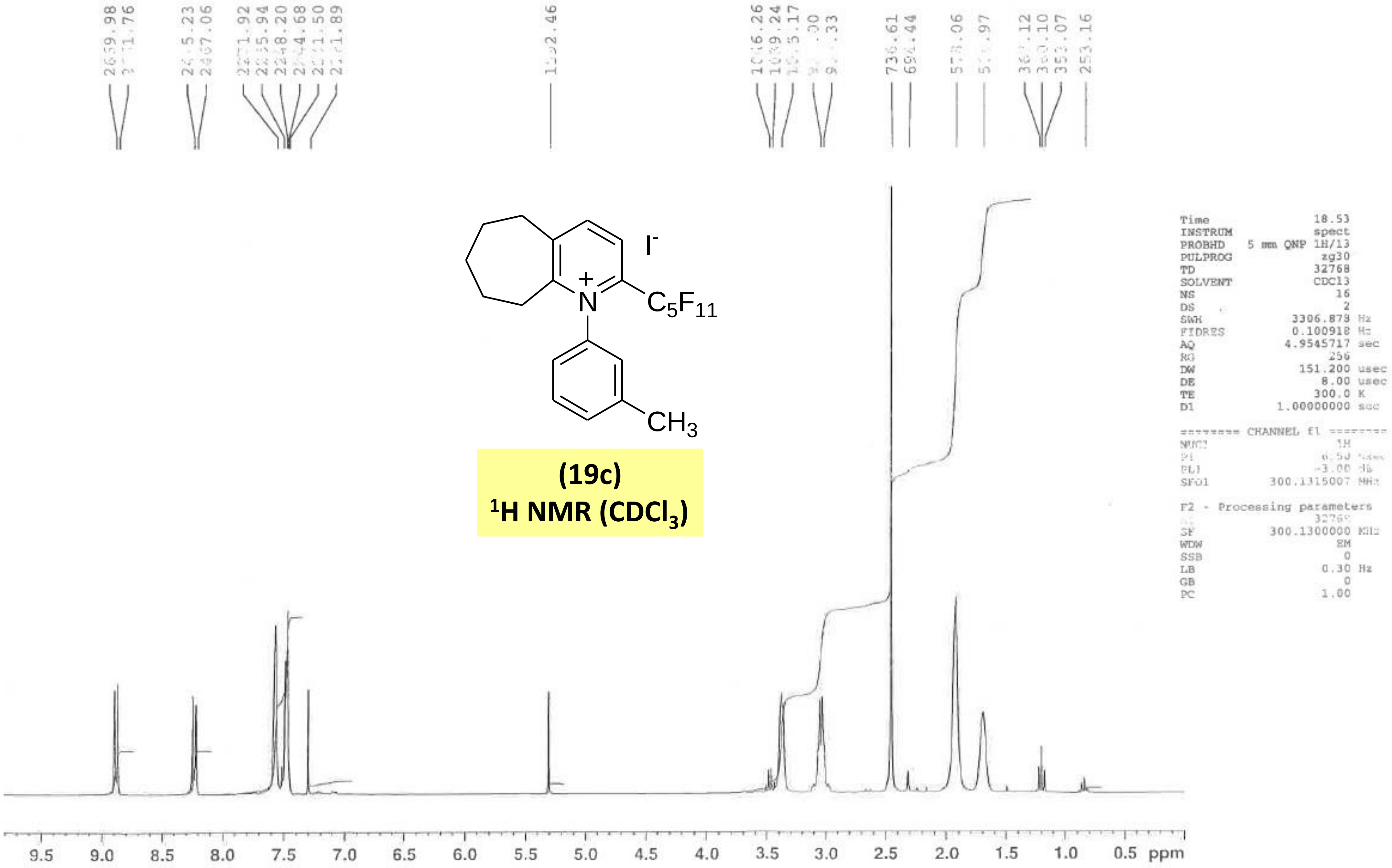

(19c)
^{1}H NMR ($CDCl_3$)

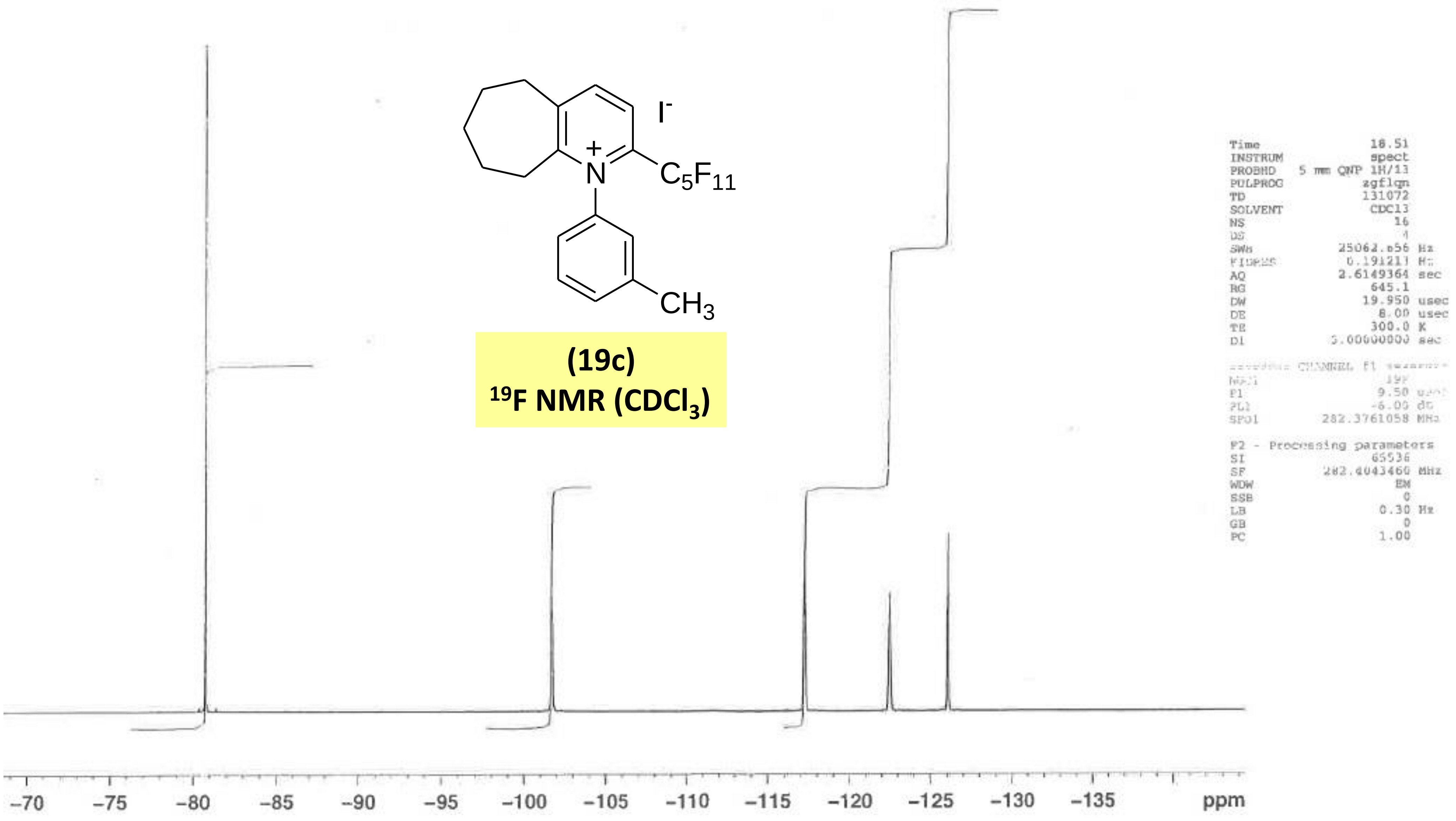

(19c)

^{19}F NMR ($CDCl_3$)

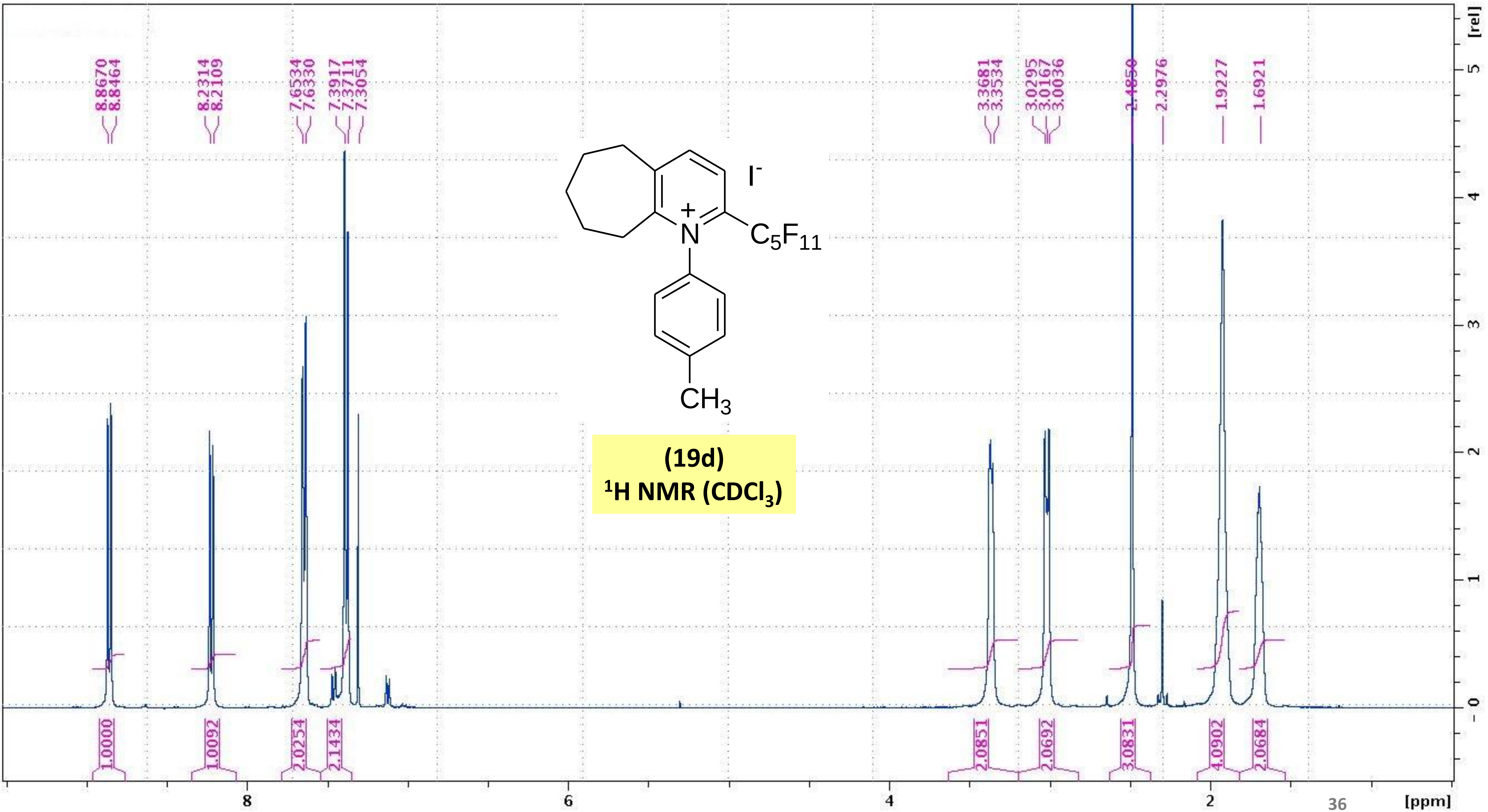

8.8670
8.8464
8.2314
8.2109
7.6534
7.6330
7.3917
7.3711
7.3054
3.3681
3.3534
3.0295
3.0167
3.0036
2.4850
2.2976
1.9227
1.6921
I⁻
+
N
C5F11
CH3
(19d)
1H NMR (CDCl3)
1.0000
1.0092
2.0254
2.1434
2.0851
2.0692
3.0831
4.0902
2.0684
8
6
4
2
[ppm]
[rel]

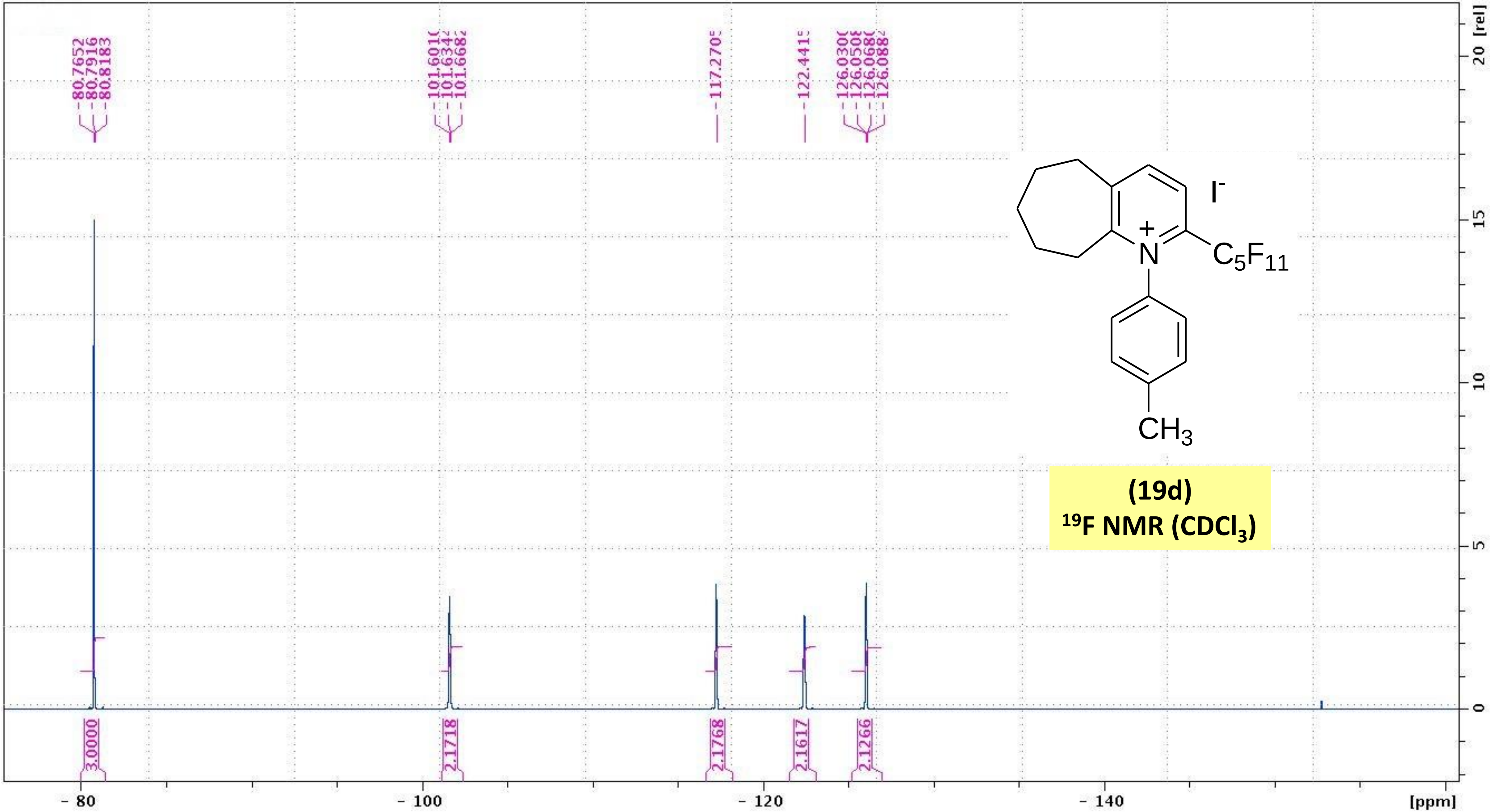

(19d)

^{19}F NMR ($CDCl_3$)

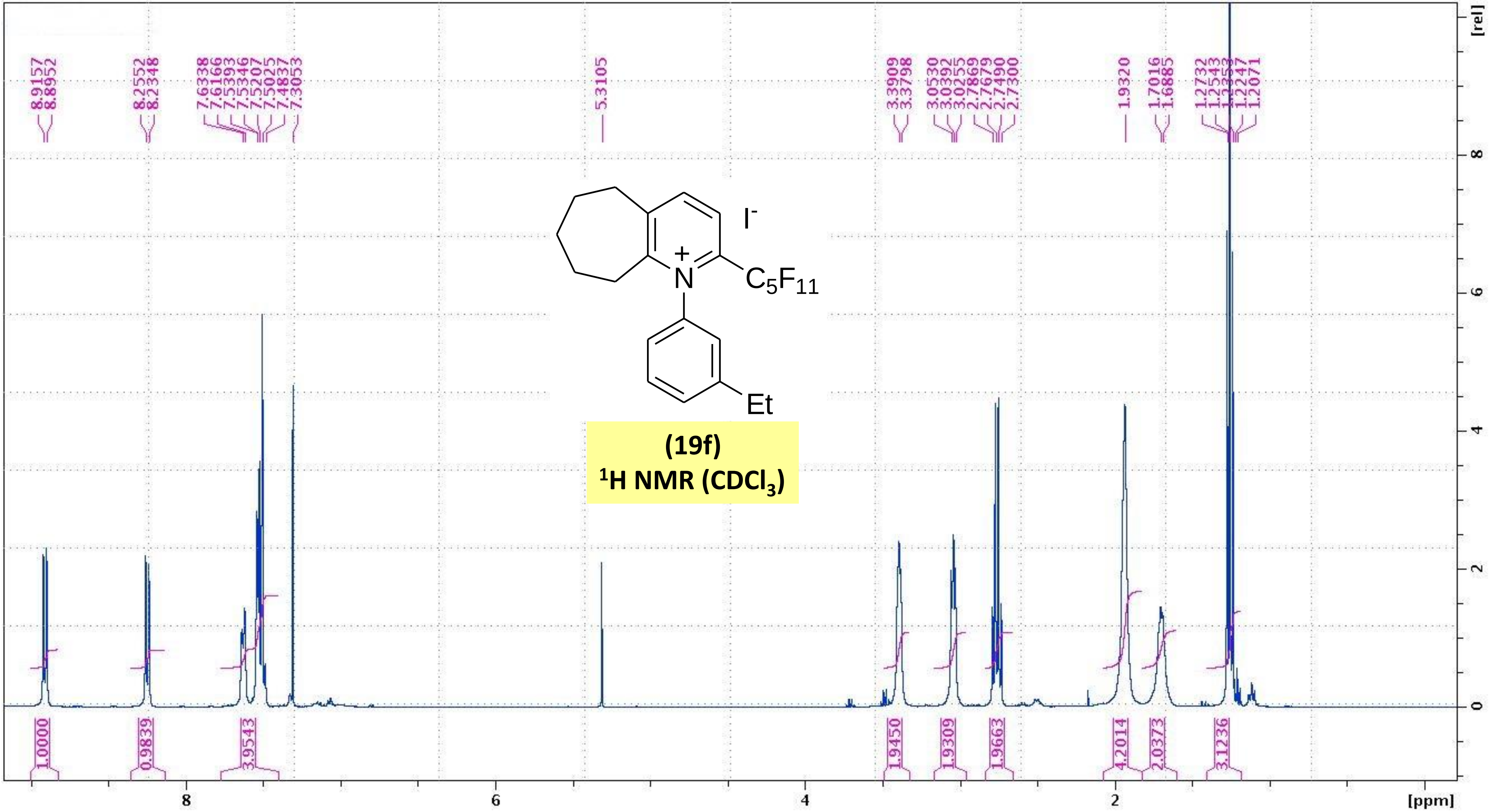
8.9157
8.8952
8.2552
8.2348
7.6338
7.6166
7.5393
7.5346
7.5207
7.5025
7.4837
7.3053
5.3105
3.3909
3.3798
3.0530
3.0392
3.0255
2.7869
2.7679
2.7490
2.7300
1.9320
1.7016
1.6885
1.2732
1.2543
1.2247
1.2071
I-
N
+
C5F11
Et
(19f)
1H NMR (CDCl3)
1.0000
0.9839
3.9543
1.9450
1.9309
1.9663
4.2014
2.0373
3.1236
8
6
4
2
[ppm]
[rel]

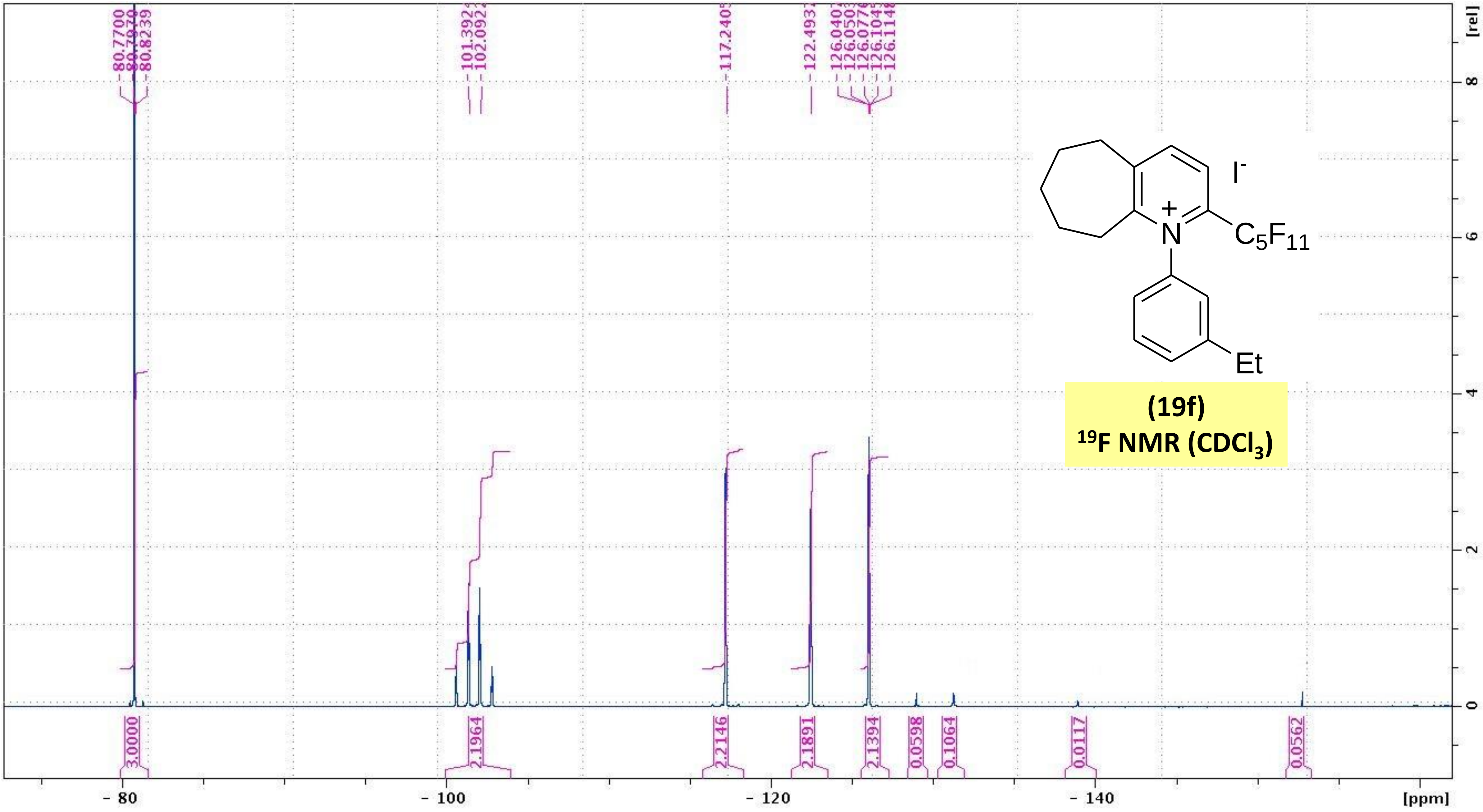

(19f)

^{19}**F NMR (CDCl$_3$)**

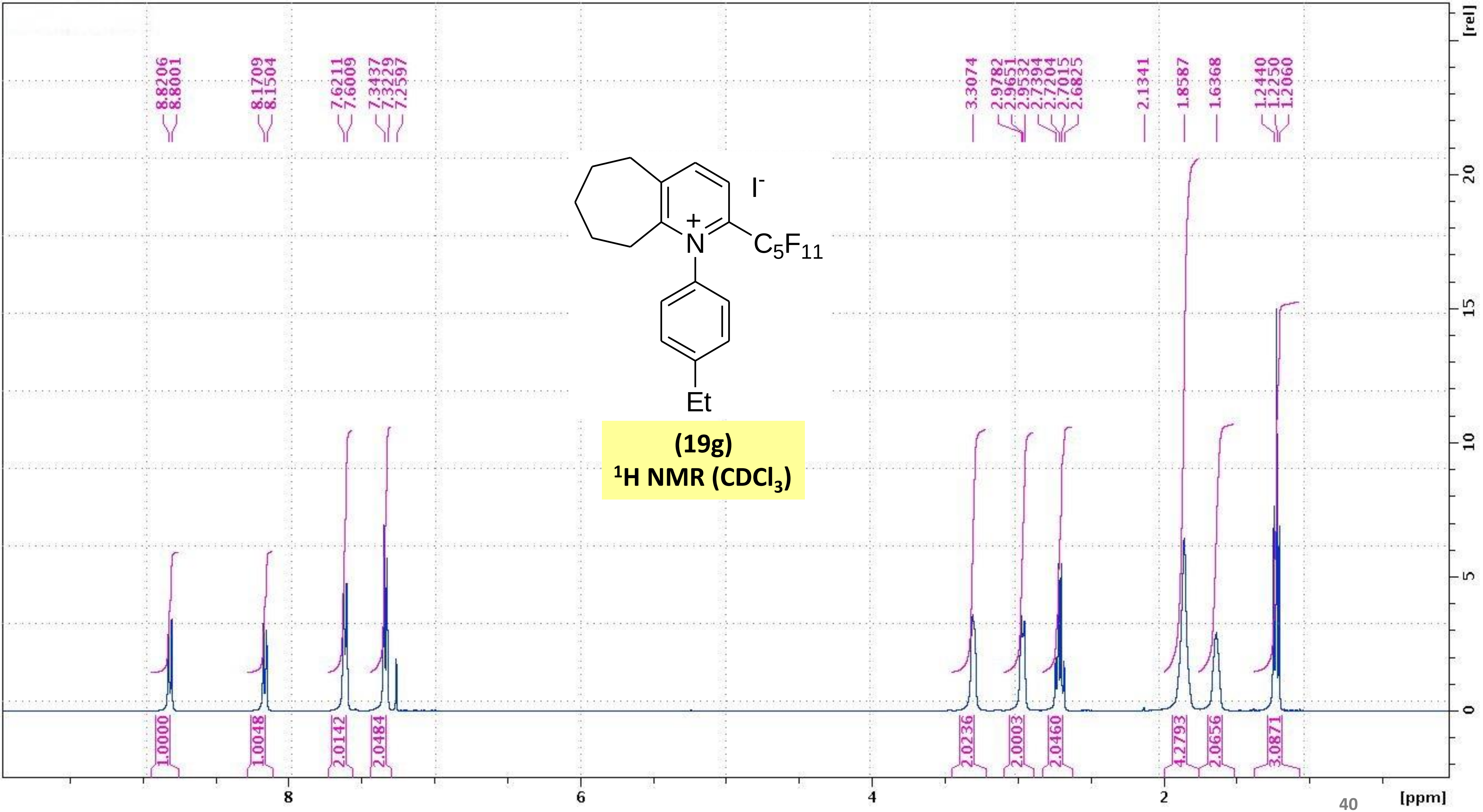

(19g)
¹H NMR (CDCl₃)
I⁻
C5F11
N+
Et
8.8206
8.8001
8.1709
8.1504
7.6211
7.6009
7.3437
7.3229
7.2597
3.3074
2.9782
2.9651
2.9532
2.7394
2.7204
2.7015
2.6825
2.1341
1.8587
1.6368
1.2440
1.2250
1.2060
1.0000
1.0048
2.0142
2.0484
2.0236
2.0003
2.0460
4.2793
2.0656
3.0871
[rel]
[ppm]

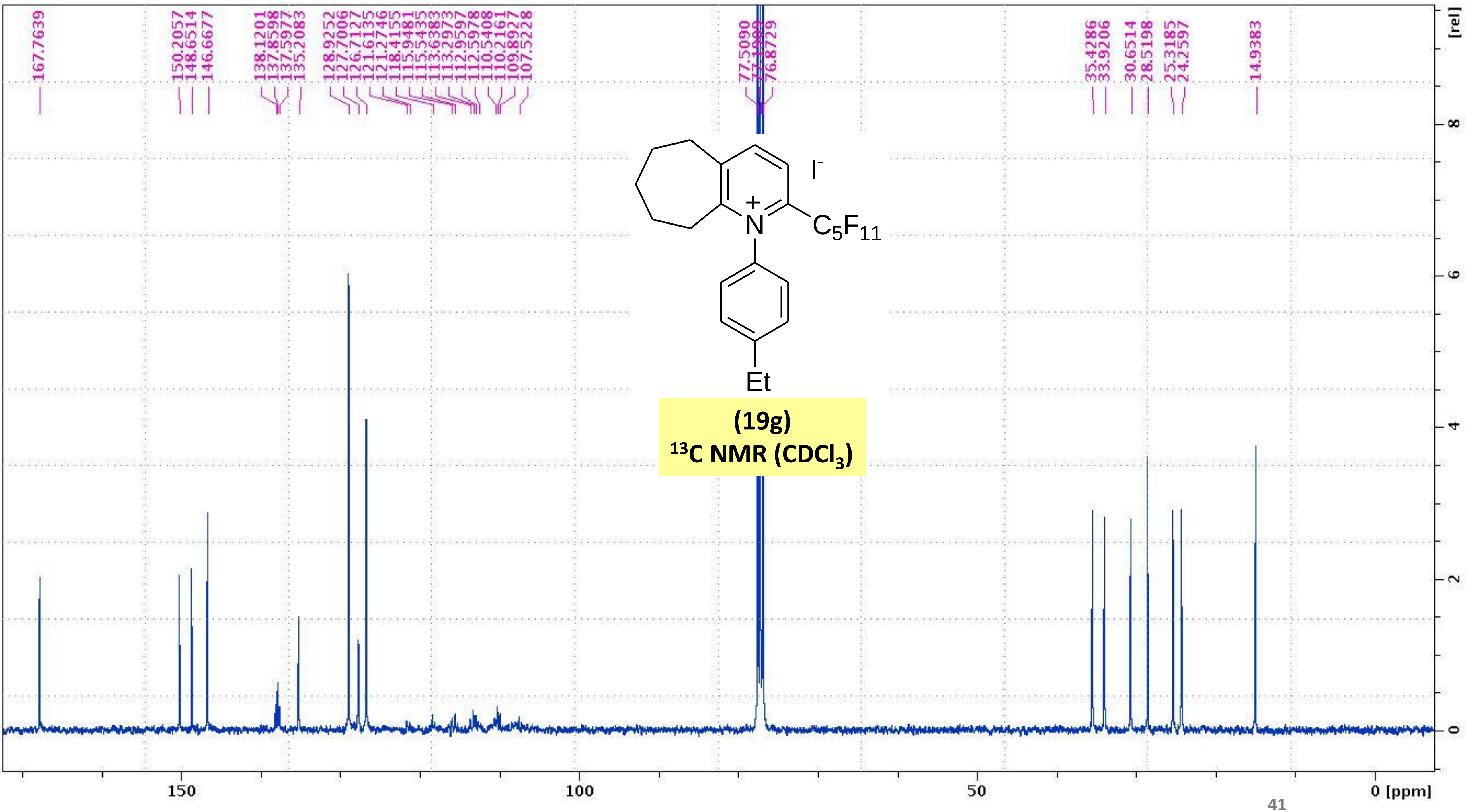
167.7639
150.2057
148.6514
146.6677
138.1201
137.8598
137.5977
135.2083
128.9252
127.7006
126.7127
121.6135
121.2746
118.4155
115.9481
115.5435
113.6383
113.2973
112.9597
112.5978
110.5408
110.2161
109.8927
107.5228
77.5090
76.8729
35.4286
33.9206
30.6514
28.5198
25.3185
24.2597
14.9383
I-
N
+
C5F11
Et
(19g)
13C NMR (CDCl3)
150
100
50
0 [ppm]
[rel]
8
6
4
2
0

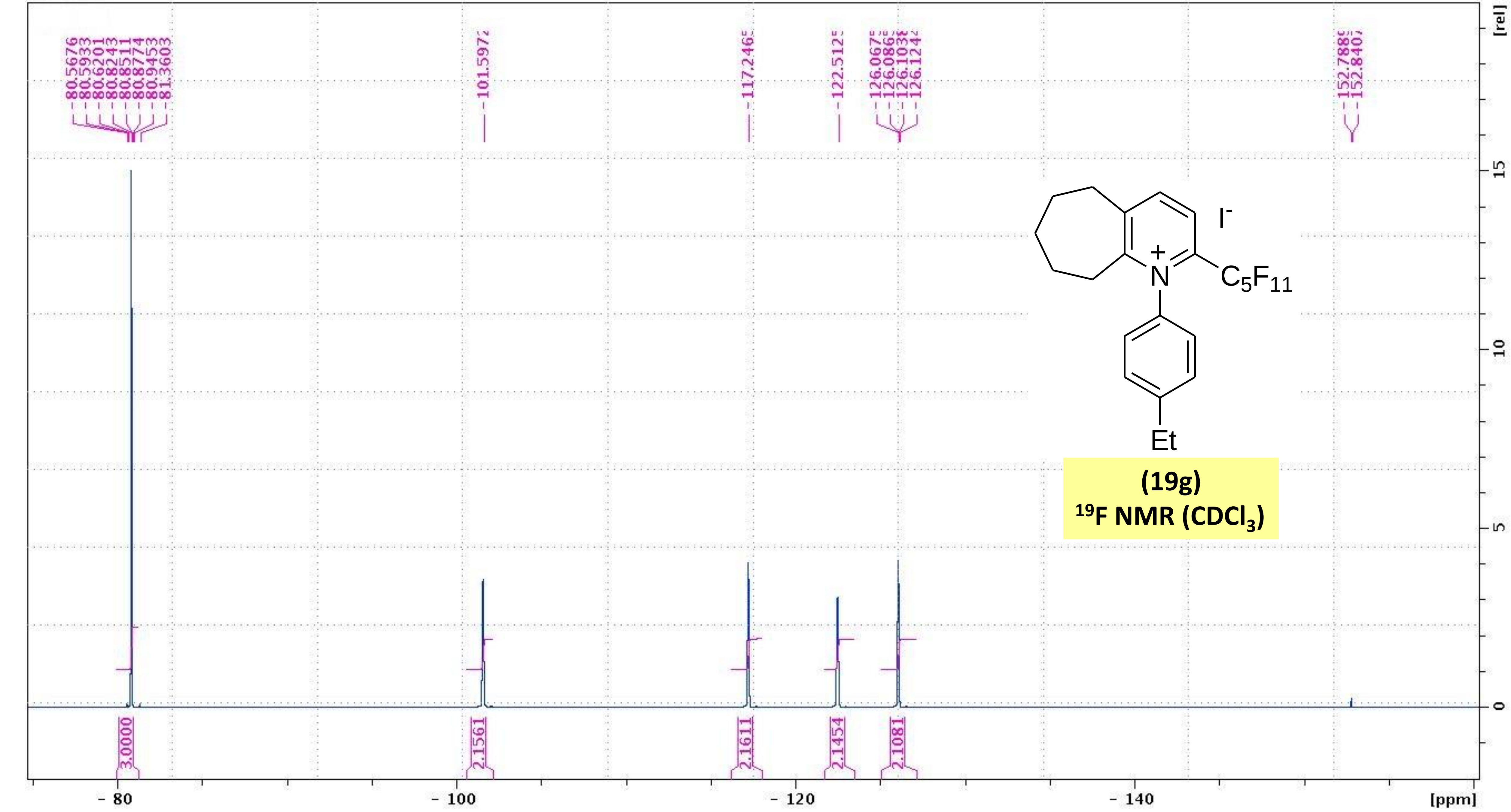

(19g)
^{19}F NMR ($CDCl_3$)

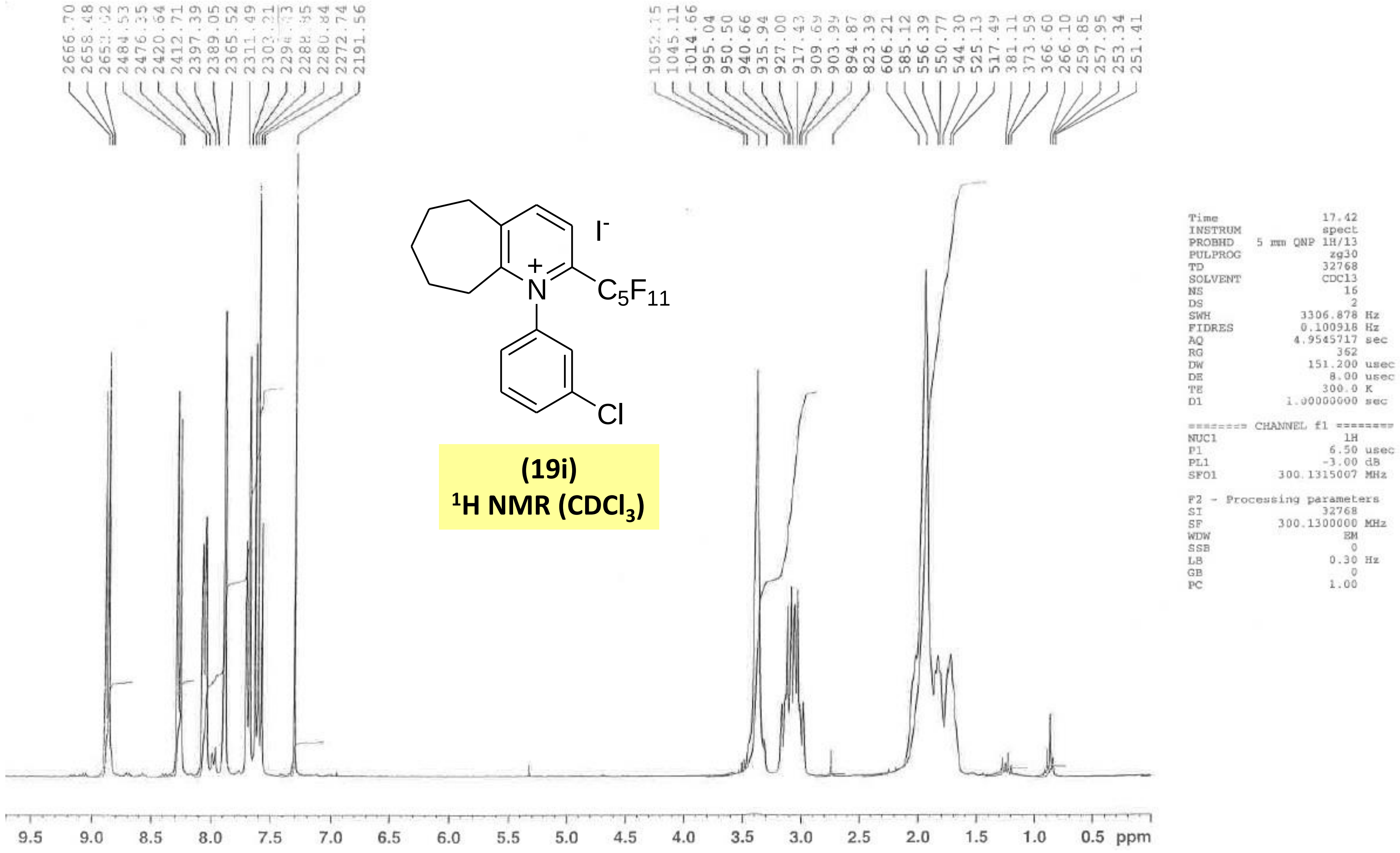

(19i)
^{1}H NMR ($CDCl_3$)

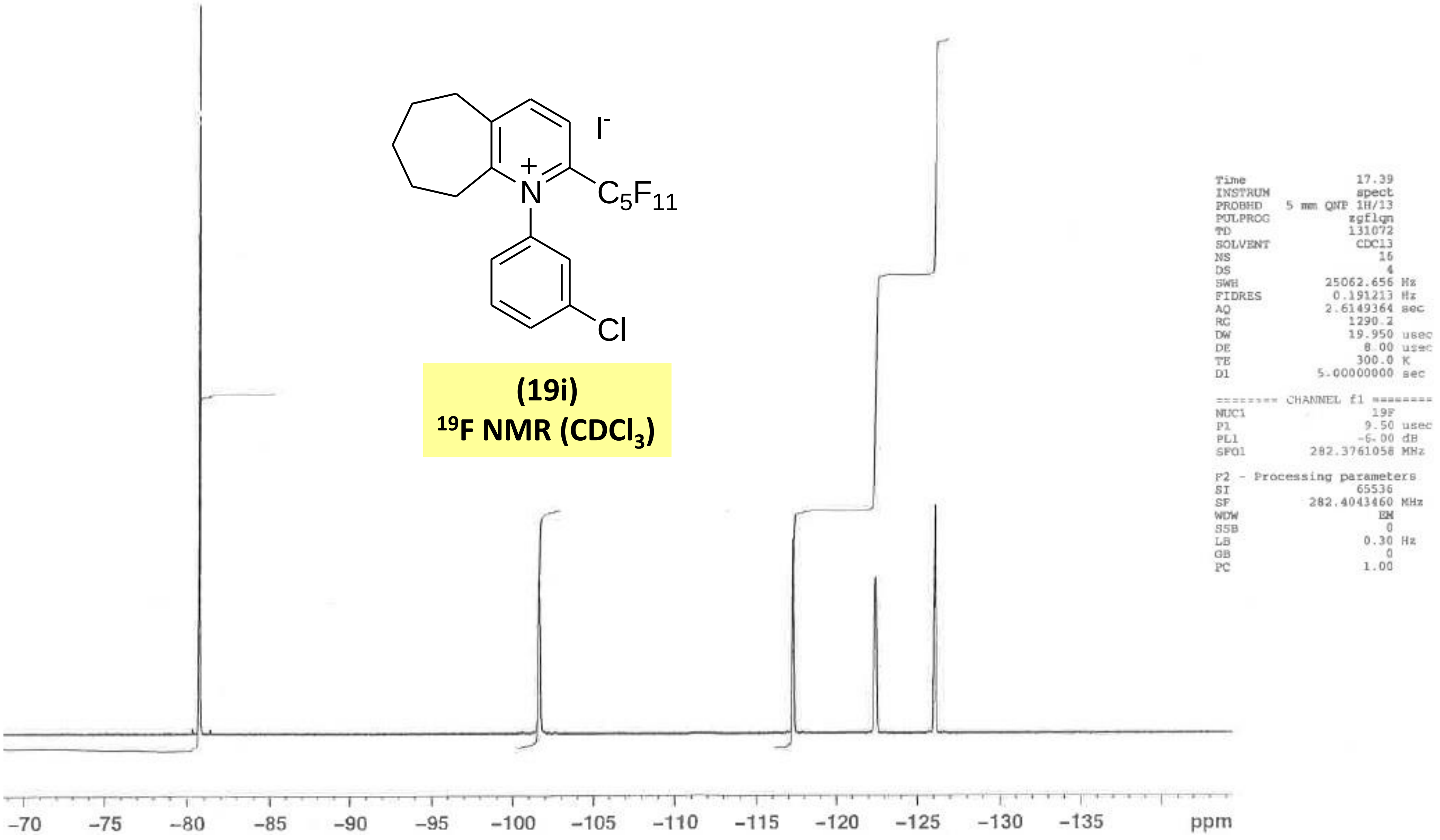

(19i)
^{19}F NMR ($CDCl_3$)

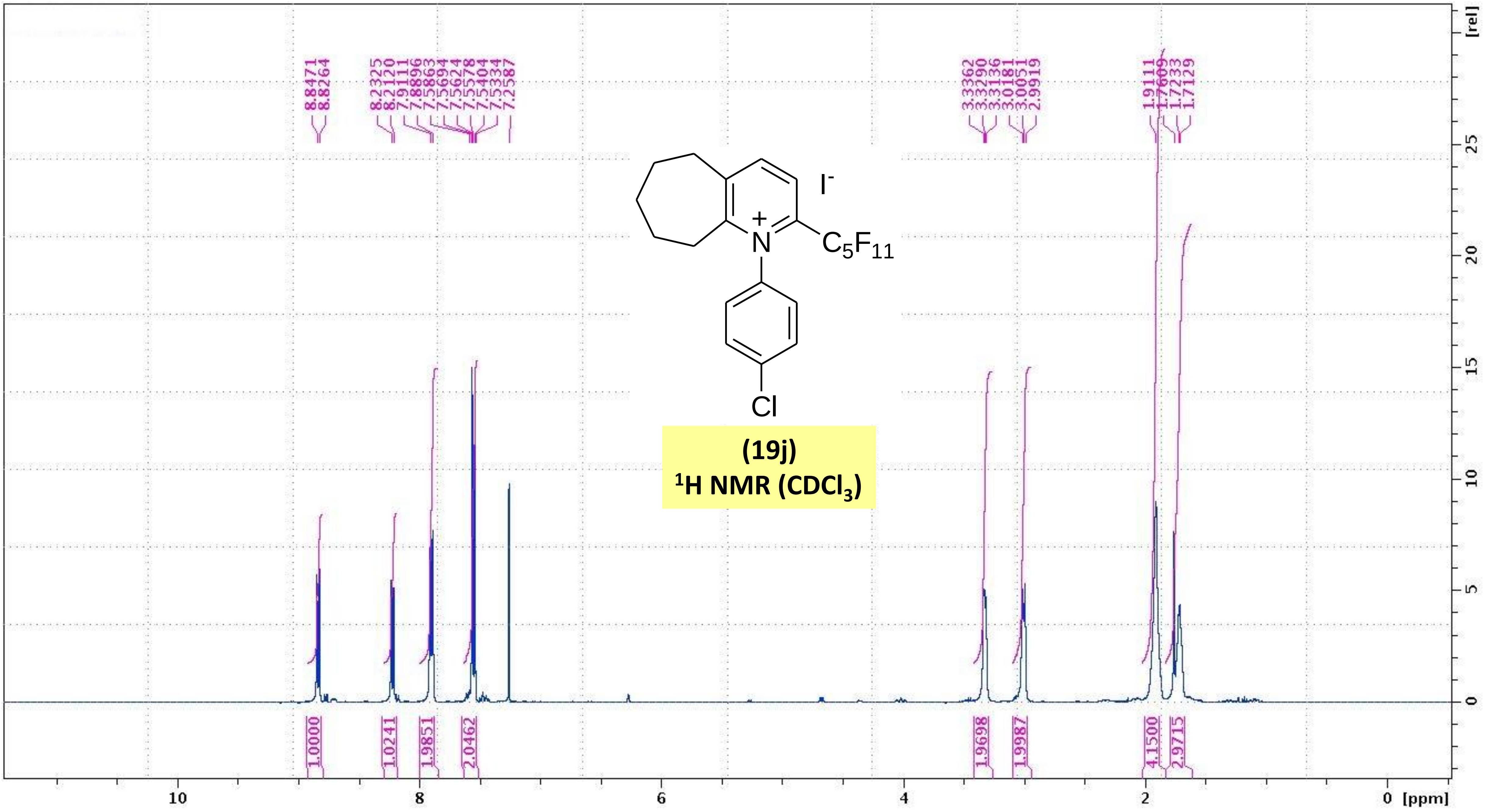
8.8471
8.8264
8.2325
8.2120
7.9111
7.8896
7.5863
7.5694
7.5624
7.5578
7.5404
7.5334
7.2587
3.3362
3.3290
3.3136
3.0181
3.0051
2.9919
1.9111
1.7609
1.7233
1.7129
I-
N+
C5F11
Cl
(19j)
1H NMR (CDCl3)
1.0000
1.0241
1.9851
2.0462
1.9698
1.9987
4.1500
2.9715
10
8
6
4
2
0 [ppm]
[rel]
0
5
10
15
20
25

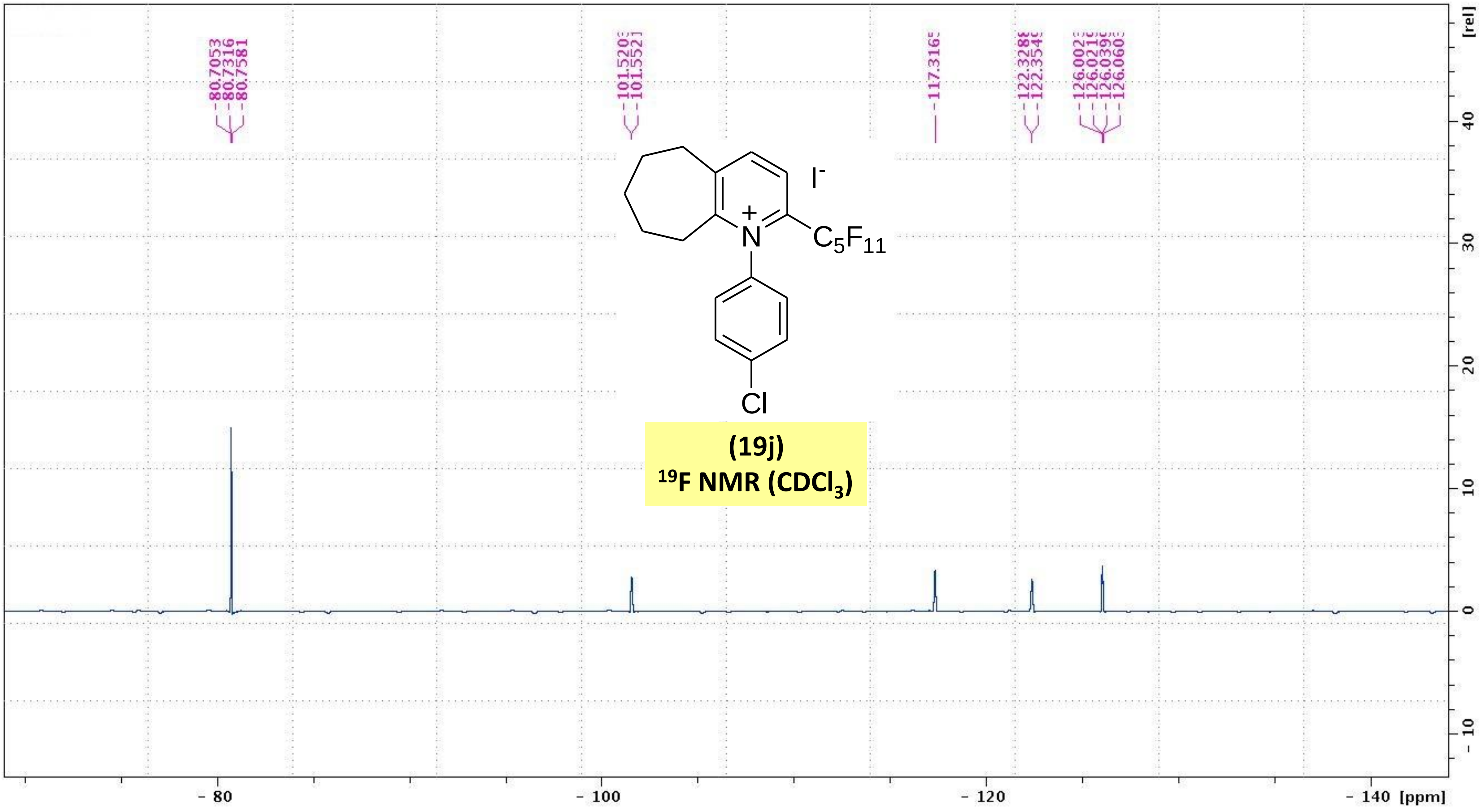

-80.7053
-80.7316
-80.7581
-101.520
-101.552
-117.316
-122.328
-122.354
-126.002
-126.021
-126.039
-126.060
I-
N+
C5F11
Cl
(19j)
19F NMR (CDCl3)
[rel]
[ppm]

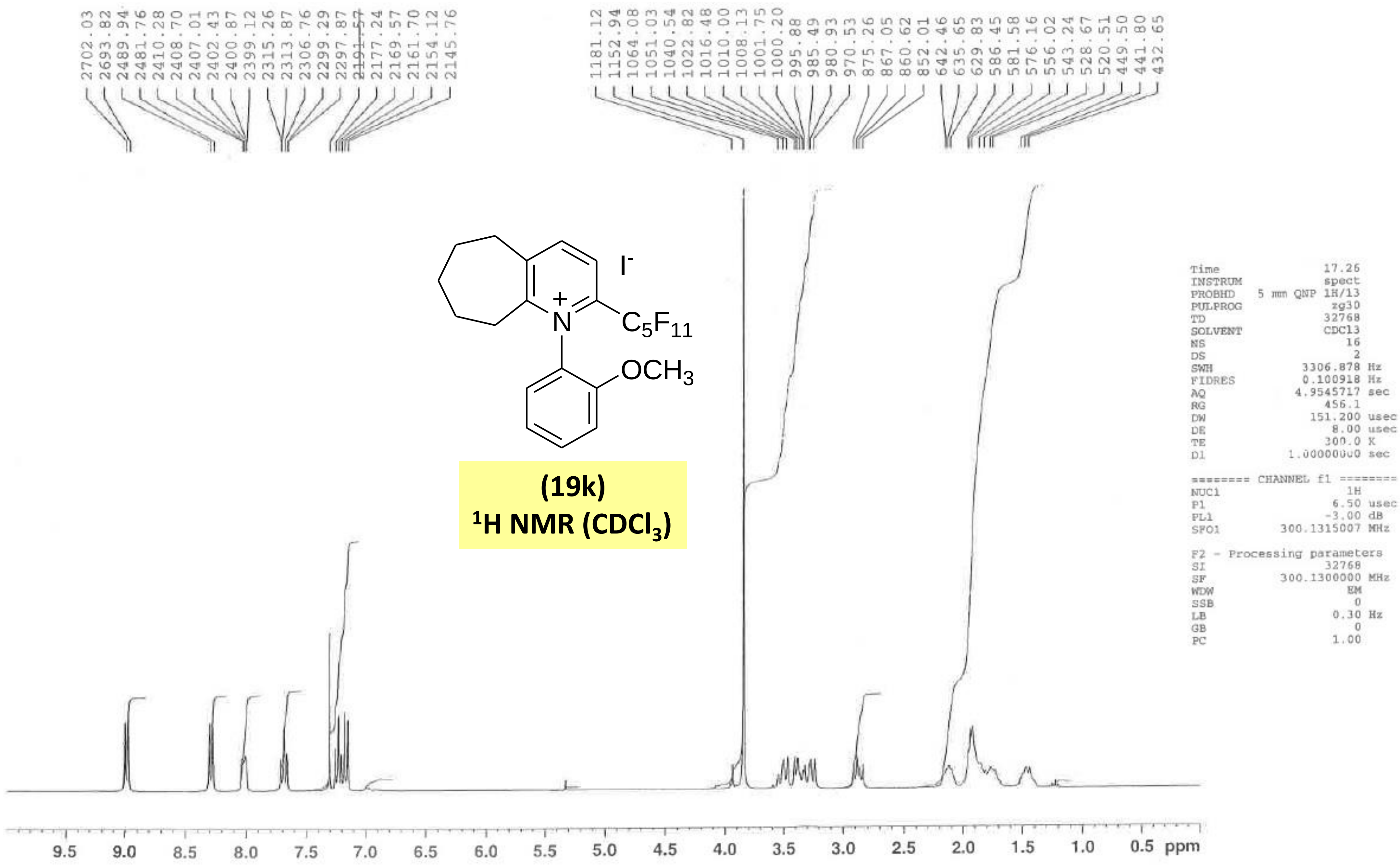

2702.03
2693.82
2489.94
2481.76
2410.28
2408.70
2407.01
2402.43
2400.87
2399.12
2315.26
2313.87
2306.76
2299.29
2297.87
2191.57
2177.24
2169.57
2161.70
2154.12
2145.76
1181.12
1152.94
1064.08
1051.03
1040.54
1022.82
1016.48
1010.00
1008.13
1001.75
1000.20
995.88
985.49
980.93
970.53
875.26
867.05
860.62
852.01
642.46
635.65
629.83
586.45
581.58
576.16
556.02
543.24
528.67
520.51
449.50
441.80
432.65
I-
N+
C5F11
OCH3
(19k)
1H NMR (CDCl3)
Time 17.26
INSTRUM spect
PROBHD 5 mm QNP 1H/13
PULPROG zg30
TD 32768
SOLVENT CDCl3
NS 16
DS 2
SWH 3306.878 Hz
FIDRES 0.100918 Hz
AQ 4.9545717 sec
RG 456.1
DW 151.200 usec
DE 8.00 usec
TE 300.0 K
D1 1.00000000 sec
======== CHANNEL f1 ========
NUC1 1H
P1 6.50 usec
PL1 -3.00 dB
SFO1 300.1315007 MHz
F2 - Processing parameters
SI 32768
SF 300.1300000 MHz
WDW EM
SSB 0
LB 0.30 Hz
GB 0
PC 1.00
9.5 9.0 8.5 8.0 7.5 7.0 6.5 6.0 5.5 5.0 4.5 4.0 3.5 3.0 2.5 2.0 1.5 1.0 0.5 ppm

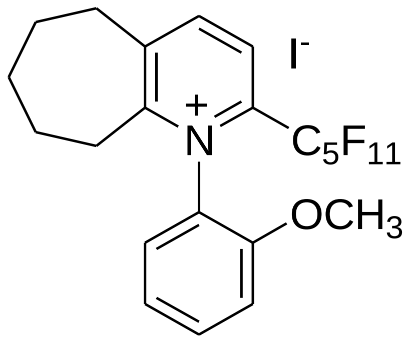

(19k)
^{19}F NMR ($CDCl_3$)

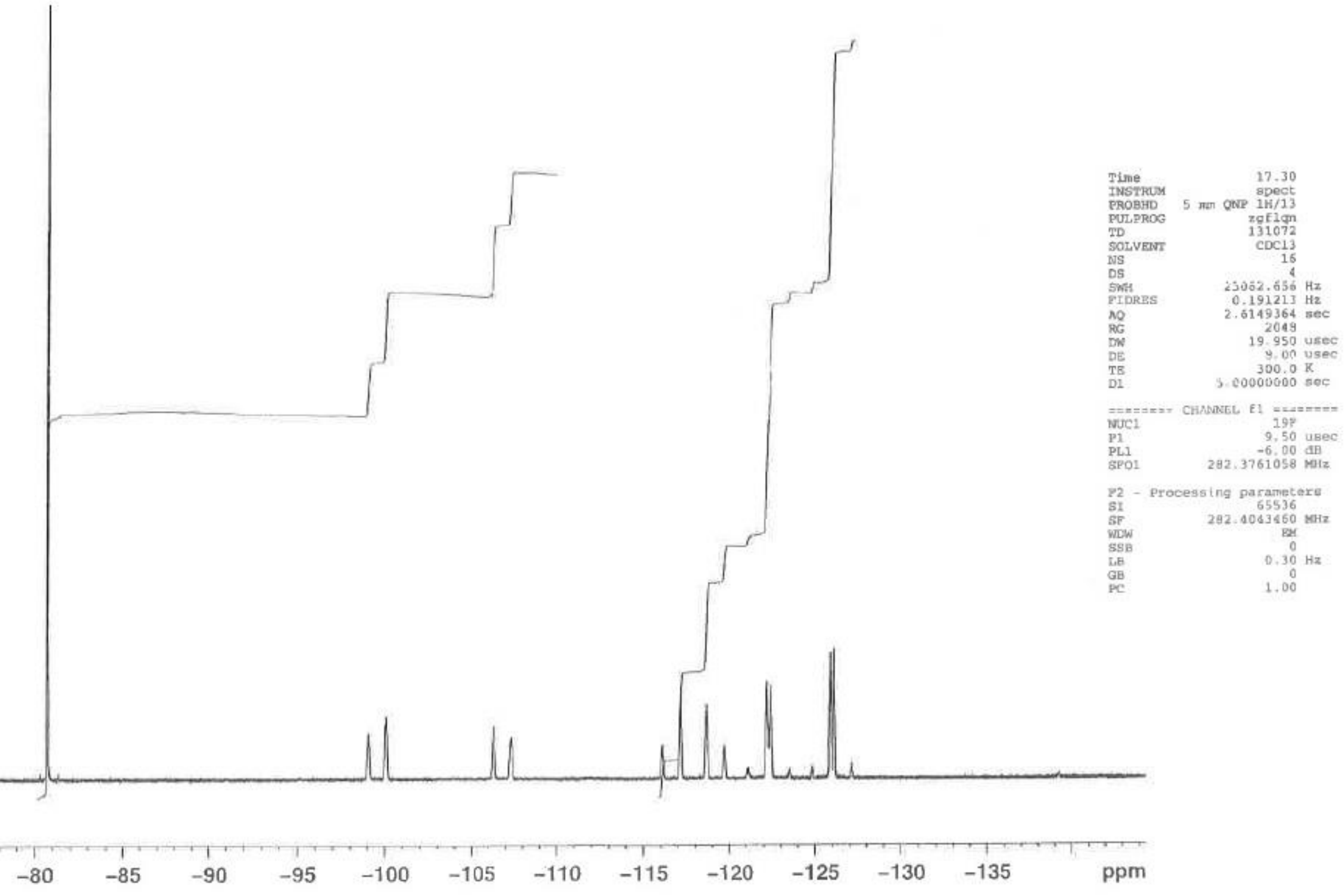

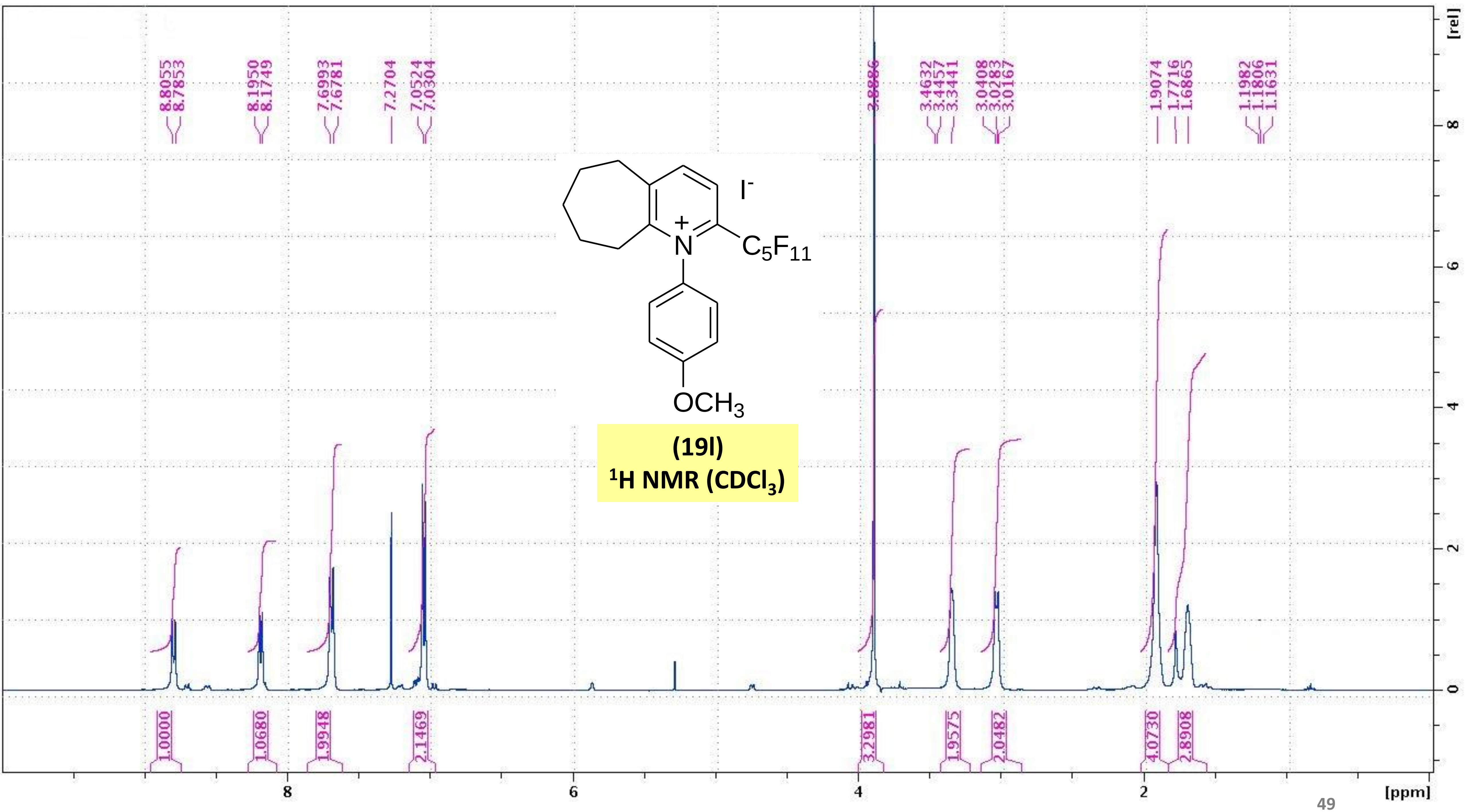
8.8055
8.7853
8.1950
8.1749
7.6993
7.6781
7.2704
7.0524
7.0304
3.8886
3.4632
3.4457
3.3441
3.0408
3.0283
3.0167
1.9074
1.7716
1.6865
1.1982
1.1806
1.1631
I-
N+
C5F11
OCH3
(19l)
1H NMR (CDCl3)
1.0000
1.0680
1.9948
2.1469
3.2981
1.9575
2.0482
4.0730
2.8908
8
6
4
2
[ppm]
[rel]

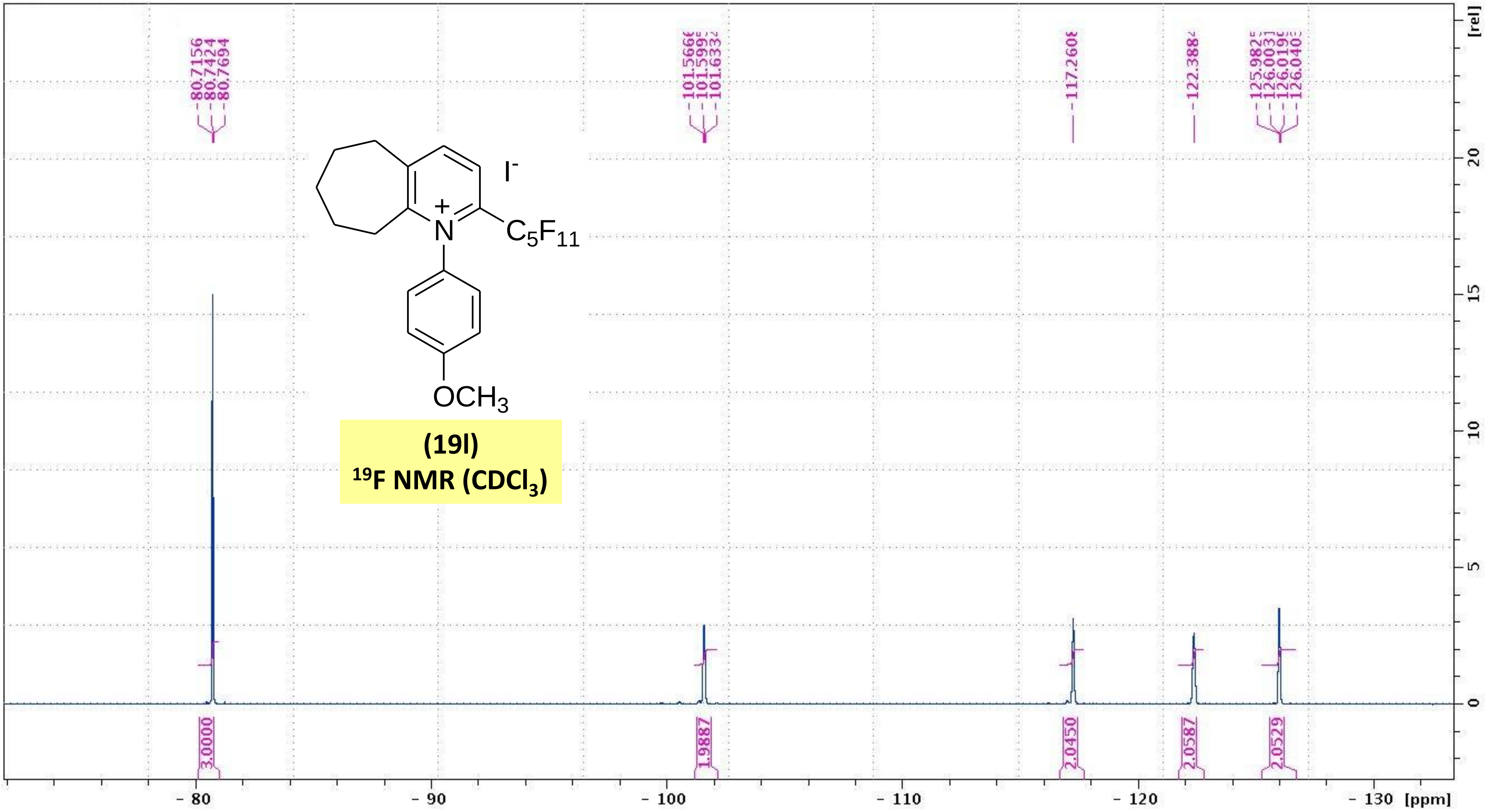

(19l)
^{19}F NMR ($CDCl_3$)

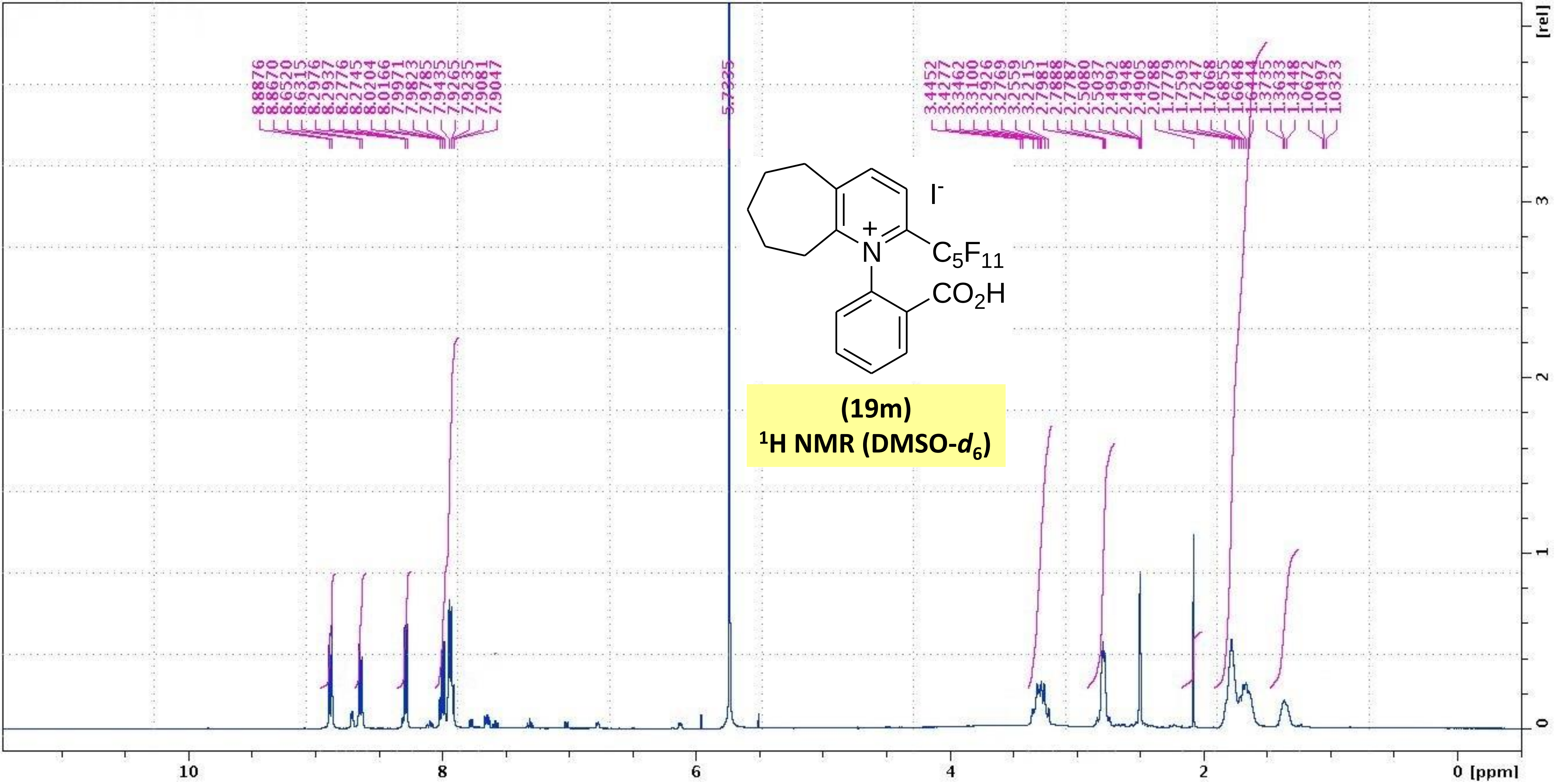

(19m)

^{1}H NMR (DMSO-d_6)

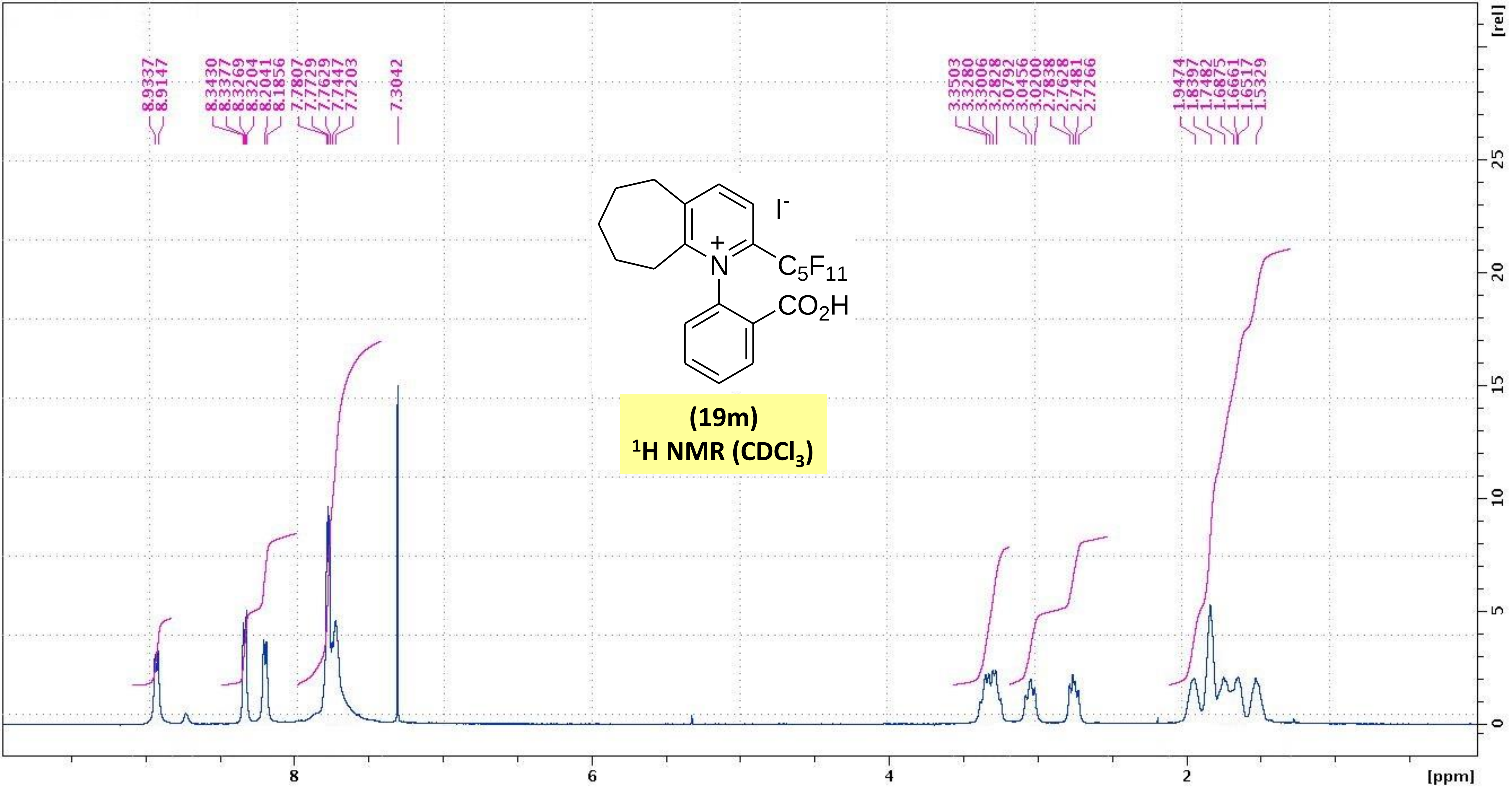
8.9337
8.9147
8.3430
8.3377
8.3269
8.3204
8.2041
8.1856
7.7807
7.7729
7.7629
7.7447
7.7203
7.3042
3.3503
3.3280
3.3006
3.2828
3.0792
3.0456
3.0200
2.7838
2.7628
2.7481
2.7266
1.9474
1.8397
1.7482
1.6875
1.6661
1.6517
1.5329
I⁻
N
C5F11
CO2H
(19m)
1H NMR (CDCl3)
[rel]
[ppm]

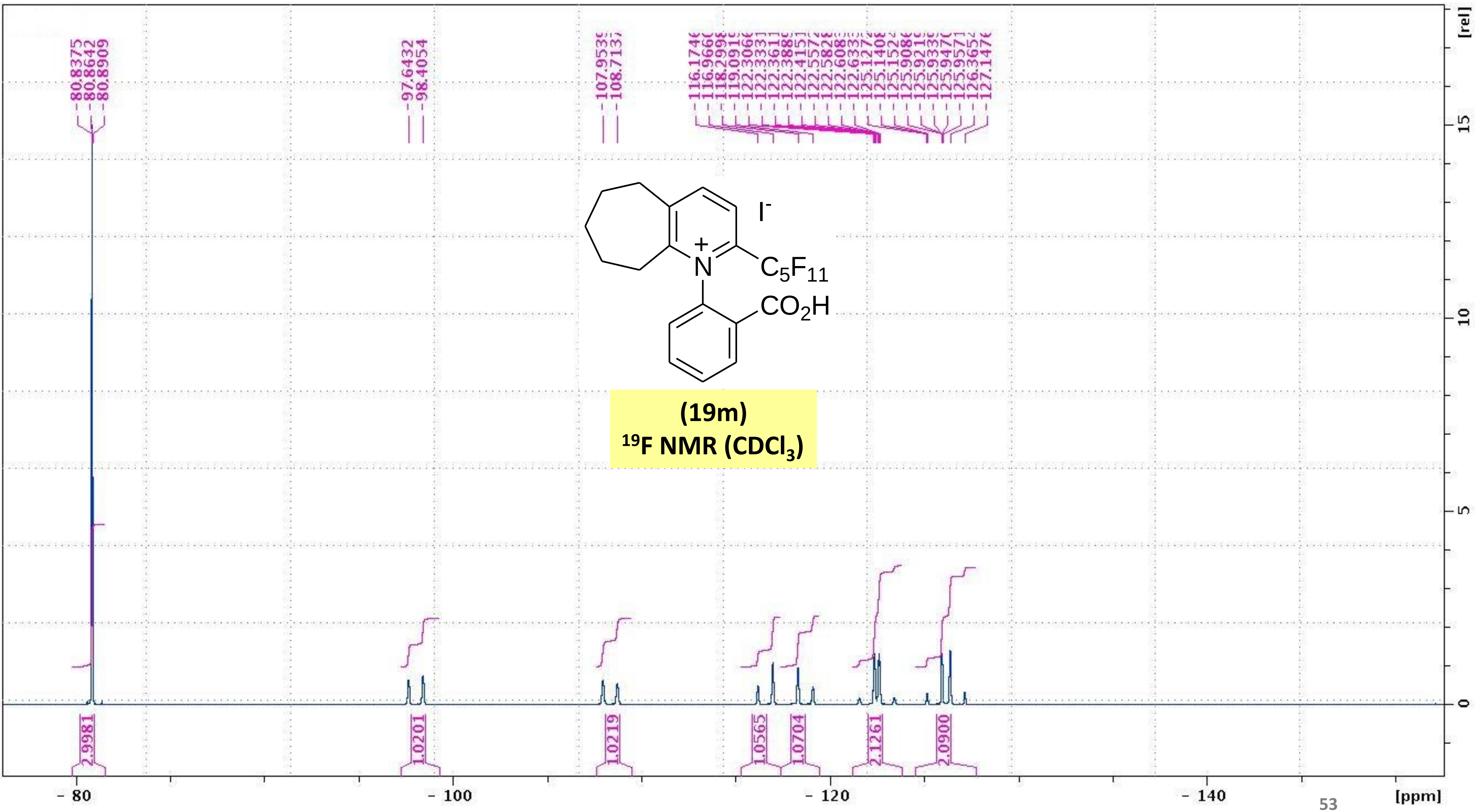

(19m)
^{19}F NMR ($CDCl_3$)
